# Synthesis, Biological Evaluation, and Docking Studies of Antagonistic Hydroxylated Arecaidine Esters Targeting mAChRs

**DOI:** 10.3390/molecules27103173

**Published:** 2022-05-16

**Authors:** Jonas Kilian, Marlon Millard, Marius Ozenil, Dominik Krause, Khadija Ghaderi, Wolfgang Holzer, Ernst Urban, Helmut Spreitzer, Wolfgang Wadsak, Marcus Hacker, Thierry Langer, Verena Pichler

**Affiliations:** 1 Department of Biomedical Imaging and Image-Guided Therapy, Division of Nuclear Medicine, Medical University of Vienna, 1090 Vienna, Austria; jonas.kilian@meduniwien.ac.at (J.K.); marius.ozenil@meduniwien.ac.at (M.O.); wolfgang.wadsak@meduniwien.ac.at (W.W.); marcus.hacker@meduniwien.ac.at (M.H.); 2 Vienna Doctoral School of Pharmaceutical, Nutritional and Sport Sciences, University of Vienna, 1090 Vienna, Austria; marlon.millard@univie.ac.at; 3 Department of Pharmaceutical Sciences, Division of Pharmaceutical Chemistry, Faculty of Life Sciences, University of Vienna, 1090 Vienna, Austria; a01449286@unet.univie.ac.at (D.K.); a01448605@unet.univie.ac.at (K.G.); wolfgang.holzer@univie.ac.at (W.H.); ernst.urban@univie.ac.at (E.U.); helmut.spreitzer@univie.ac.at (H.S.); thierry.langer@univie.ac.at (T.L.); 4 CBmed GmbH—Center for Biomarker Research in Medicine, 8036 Graz, Austria

**Keywords:** muscarinic acetylcholine receptors, drug development, molecular docking

## Abstract

The muscarinic acetylcholine receptor family is a highly sought-after target in drug and molecular imaging discovery efforts aimed at neurological disorders. Hampered by the structural similarity of the five subtypes’ orthosteric binding pockets, these efforts largely failed to deliver subtype-selective ligands. Building on our recent successes with arecaidine-derived ligands targeting M_1_, herein we report the synthesis of a related series of 11 hydroxylated arecaidine esters. Their physicochemical property profiles, expressed in terms of their computationally calculated CNS MPO scores and HPLC-logD values, point towards blood–brain barrier permeability. By means of a competitive radioligand binding assay, the binding affinity values towards each of the individual human mAChR subtypes *h*M_1_–*h*M_5_ were determined. The most promising compound of this series **17b** was shown to have a binding constant towards *h*M_1_ in the single-digit nanomolar region (5.5 nM). Similar to our previously reported arecaidine-derived esters, the entire series was shown to act as *h*M1R antagonists in a calcium flux assay. Overall, this study greatly expanded our understanding of this recurring scaffolds’ structure–activity relationship and will guide the development towards highly selective mAChRs ligands.

## 1. Introduction

Muscarinic acetylcholine receptor (mAChR) M_1_, as the most prevalent subtype and in accordance with its abundant expression in all major forebrain areas, has been implicated in the pathophysiology of various neurological diseases such as Alzheimer’s disease, Parkinson’s disease, and schizophrenia [1]. Hence, the muscarinic receptor subtype M_1_ has emerged as an attractive drug target for the treatment, among others, for the aforementioned disorders [2,3,4]. In spite of the spurred interest in this receptor, success stories of the development of therapeutic or diagnostic agents targeting M_1_ are lacking. One major hurdle to overcome is the high degree of sequence homology of the orthosteric binding site among the five subtypes (M_1_–M_5_) of mAChRs [5], rendering subtype-specific ligand design a challenging task. As a result, candidates entering the clinical stage are commonly plagued by dose-limiting side effects due to poor subtype selectivity profile [6]. Despite the vast body of research addressing muscarinic signaling in the central nervous system (CNS), our current understanding is still limited [7]. Positron emission tomography (PET), a molecular imaging technique allowing extensive, noninvasive studies in humans, has tremendous potential to not only advance this understanding but also to guide the development of novel brain-penetrable subtype-selective therapeutics targeting mAChRs with the possibility to quantify target engagement and occupancy [8,9].

**Figure 1:**
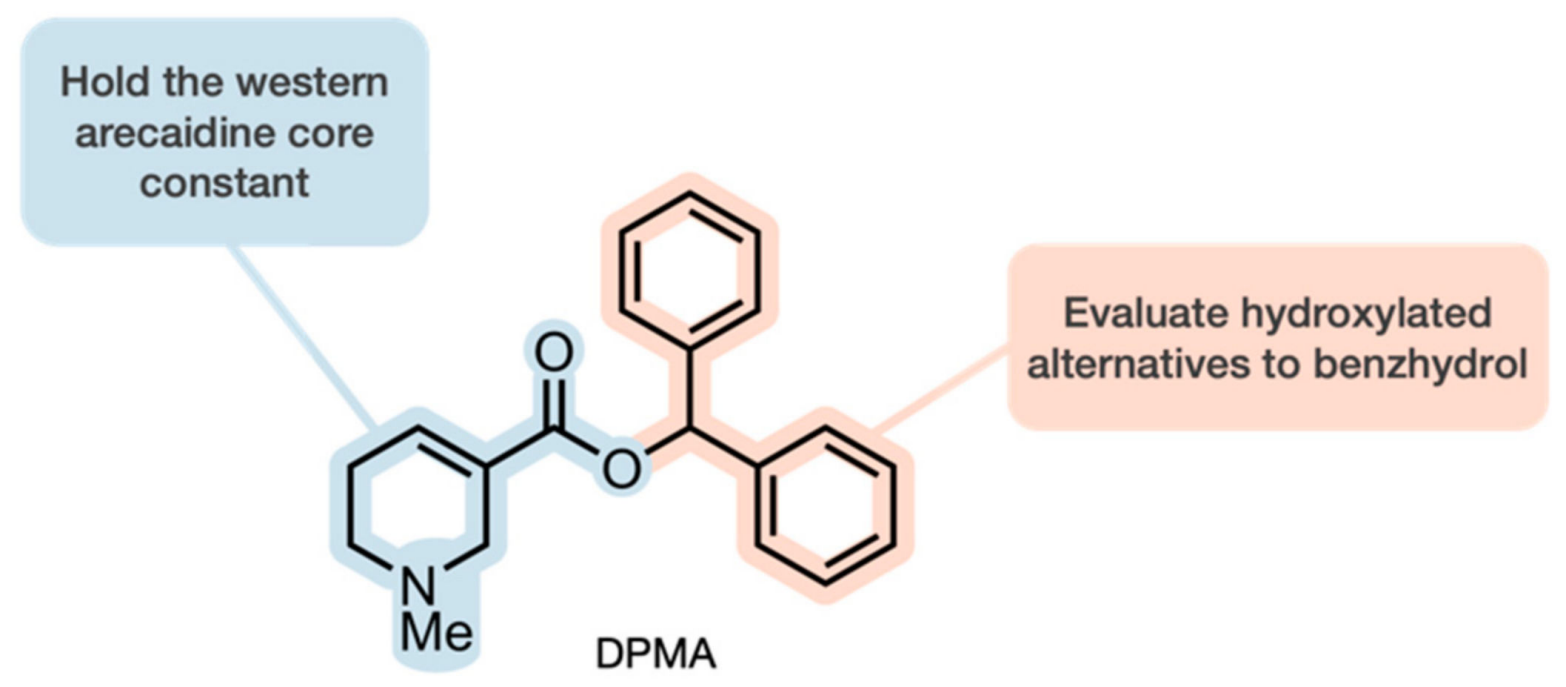
General structural modification strategy of parent compound DPMA [10].

As a continuation of our group’s research efforts towards the development of subtype selective CNS PET radiotracers for the mAChRs, we recently have shown the benzhydrol ester of arecaidine benzhydryl 1-methyl-1,2,5,6-tetrahydropyridine-3-carboxylate (DPMA, Figure 1) to display an affinity and subtype selectivity profile towards M_1_ theoretically suited for such applications [10]. Unfortunately, the excessive extent of nondisplaceable binding (NDB) renders DPMA unsuitable for PET applications. To potentially overcome this hurdle, we replaced the benzhydrol motif with a hydrobenzoin substituent, leading to slightly inferior binding properties but markedly lower lipophilicity [11]. Building on this prior research, herein we report our most recent efforts to refine our structure–activity relationship (SAR) understanding of arecaidine-derived esters. Spurred by the promising hydrobenzoin esters, we sought to synthesize and biologically evaluate a range of systematically hydroxylated arecaidine esters and subsequently study their physicochemical and thermodynamic binding properties towards the five subtypes of mAChRs. In a last step, we sought to computationally evaluate selected ligands from this series in a set of docking studies to identify whether the hydroxylated motifs were able to capture additional hydrogen bonding interactions in the orthosteric binding site.

## 2. Results and Discussion

### 2.1. Chemistry

**Scheme 1:**
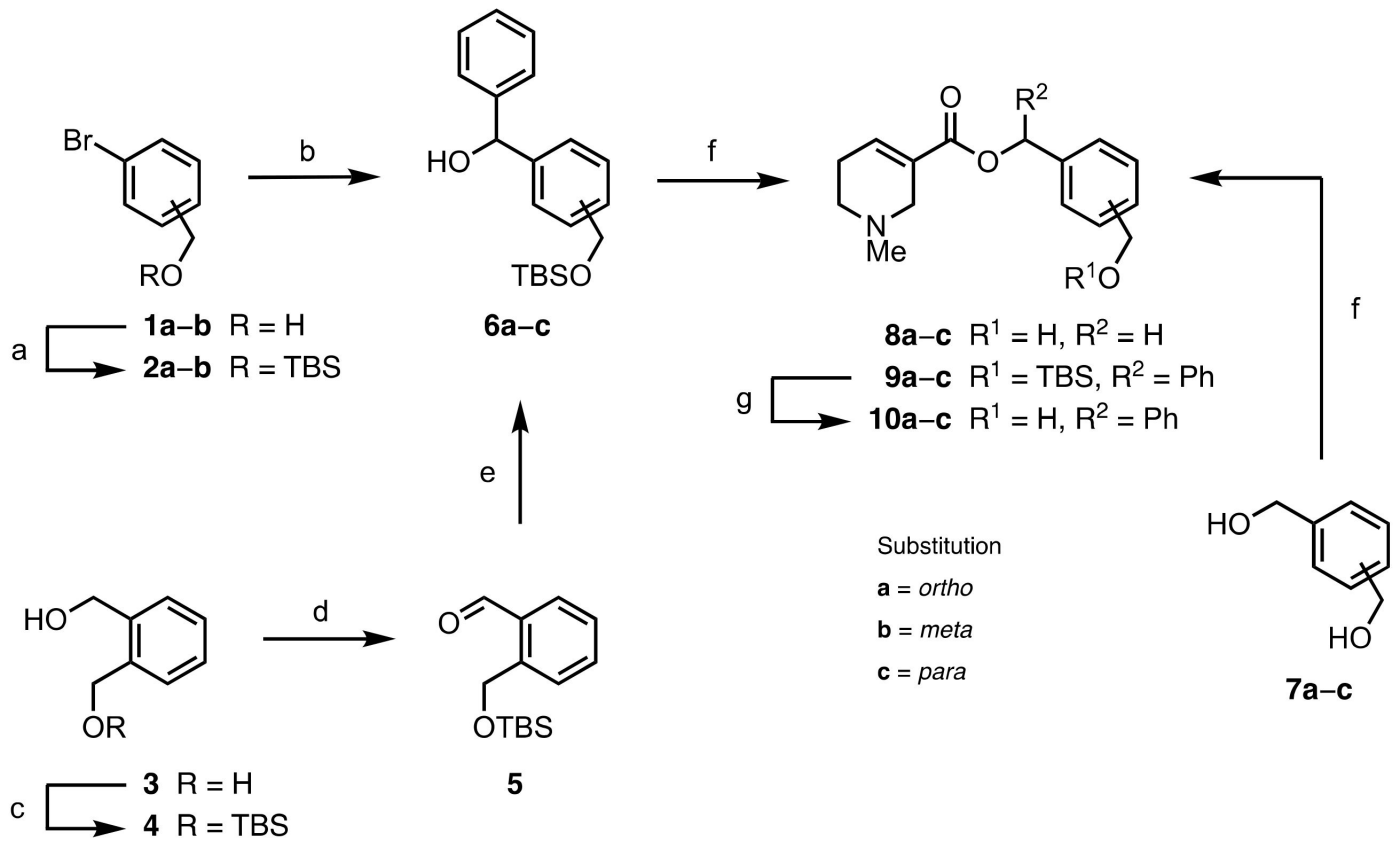
Reagents and conditions: (a) TBSCl, imidazole, DMF, 0 °C; (b) PhCHO, *n*-BuLi, THF, −78 °C → rt; (c) TBSCl, NaH, THF, 0 °C → rt; (d) MnO_2_, *n*-hexane, 10 °C; (e) PhMgBr, THF, 0 °C → rt; (f) arecaidine, EDC-HCl, 4-DMAP, CH_2_Cl_2_, rt; (g) TBAF, THF, 0 °C.

**Scheme 2:**
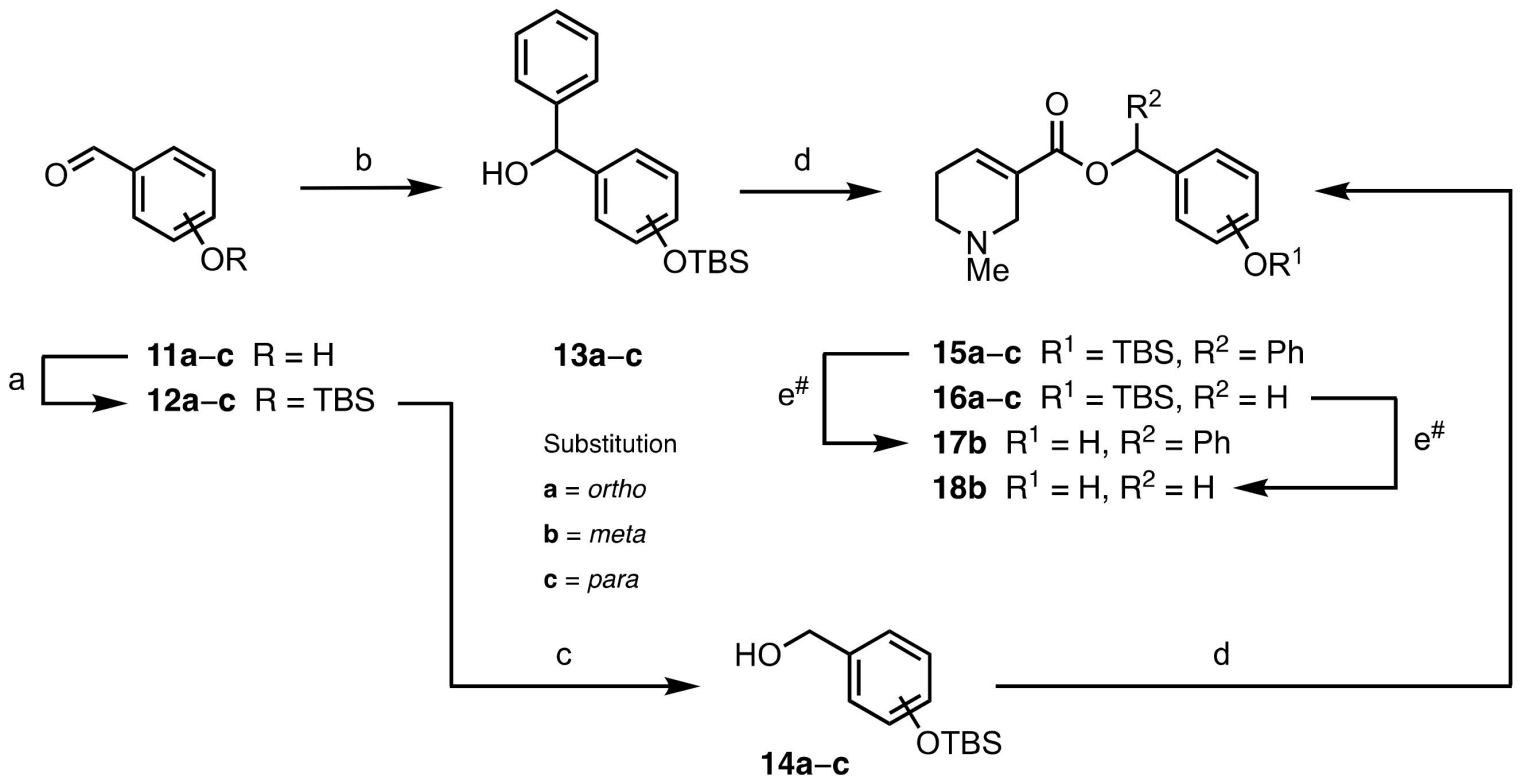
Reagents and conditions: (a) TBSCl, imidazole, DMF, 0 °C; (b) PhMgBr, THF, 0 °C → rt; (c) NaBH_4_, MeOH, 0 °C; (d) arecaidine, EDC-HCl, 4-DMAP, CH_2_Cl_2_, rt; (e) TBAF, THF, 0 °C; ^#^**15a**, **15c**, **16a**, and **16c** decomposed under these conditions.

**Scheme 3:**
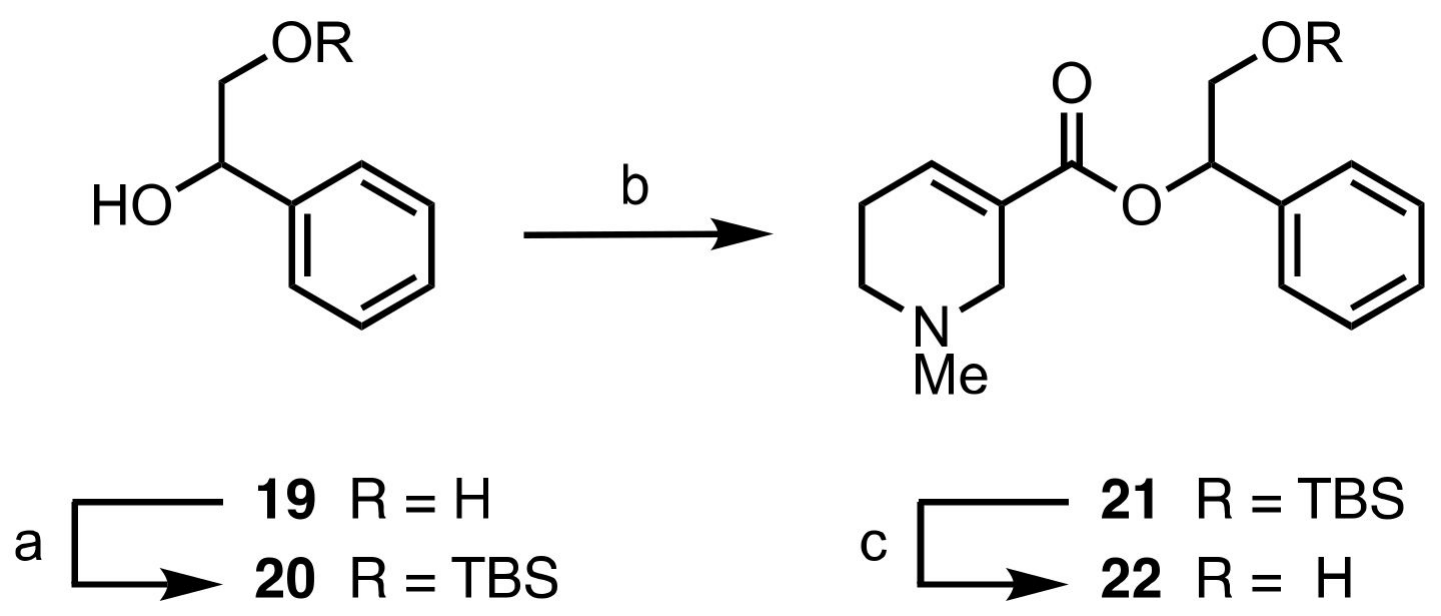
Reagents and conditions: (a) TBSCl, imidazole, CH_2_Cl_2_, 0 °C; (b) arecaidine, EDC-HCl, 4-DMAP, CH_2_Cl_2_, rt; (c) TBAF, THF, 0 °C.

**Scheme 4:**
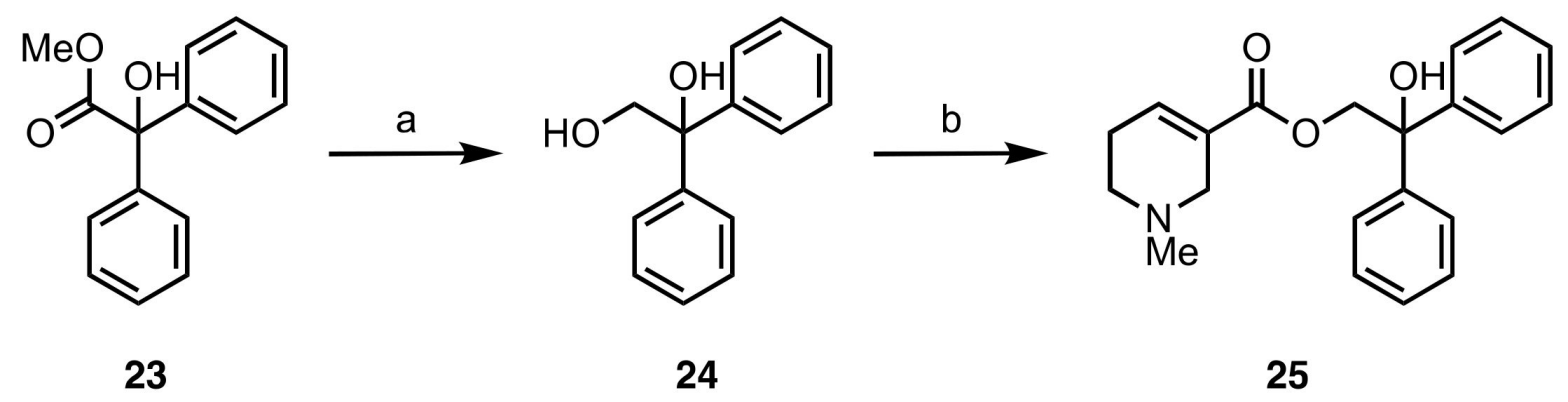
Reagents and conditions: (a) NaBH_4_, 1,4-dioxane/water 1:5, 70 °C; (b) arecaidine, EDC-HCl, 4-DMAP, CH_2_Cl_2_, rt.

**Scheme 5:**
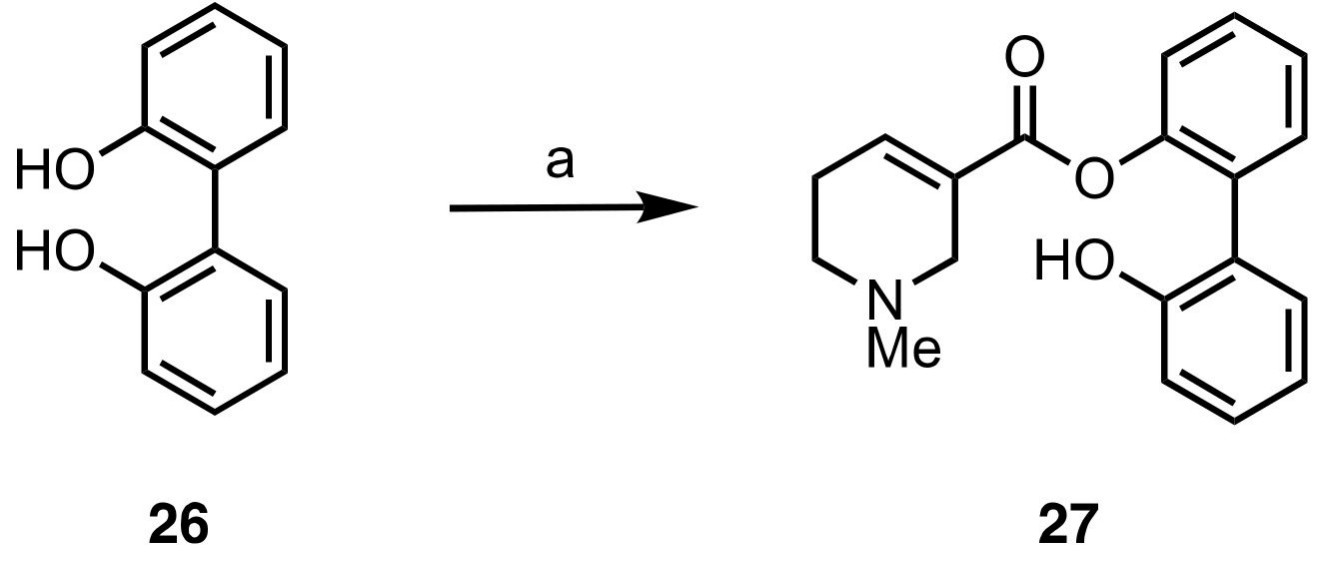
Reagents and conditions: (a) arecaidine, EDC-HCl, 4-DMAP, CH_2_Cl_2_, rt.

Before diving into the specifics of the followed synthetic sequences, the esterification step shall be put into the spotlight. Previous studies from our lab have shown CDI to be a suitable coupling agent for the esterification of arecaidine with bulky alcohols such as benzhydrol or hydrobenzoin albeit suffering from rather poor yields [10,11]. Hence, we sought to address this issue. Gratifyingly, there was no need for lengthy optimization efforts, since we found that treating a mixture of arecaidine and 4,4′-difluorobenzhydrol under Steglich conditions using EDC-HCl furnished 4-FBA with a 74% yield compared to the 9% yield using the CDI-mediated procedure [10]. Equipped with a promising esterification protocol, we focused our attention on the synthesis of the desired hydroxylated arecaidine esters. The reaction sequences followed for their synthesis are outlined in Scheme 1, Scheme 2, Scheme 3, Scheme 4 and Scheme 5. For some of the compounds, the presence of a hydroxy or hydroxymethyl leads to the loss of a plane of symmetry in comparison to 4-FBA, and thus induces chirality. In this work, however, only racemates were synthesized to speed up subsequent preliminary biological testing.

The hydroxymethyl group-bearing benzyl esters **8a**–**c** were derived in good yields (73–83%) from arecaidine and their commercially available benzenedimethanol precursors following the above-mentioned Steglich esterification using EDC-HCl as coupling reagent and 4-DMAP as nucleophilic catalyst (Scheme 1). The synthesis of the benzhydrol esters **10a**–**c** followed a slightly longer synthetic sequence. Following quantitative TBS protection, 3- and 4-bromobenzyl alcohol (**1b** and **1c**) underwent lithium–halogen exchange upon treatment with *n*-BuLi. Quenching the lithium species with benzaldehyde furnished the desired hydroxymethyl-substituted benzhydrols **6b** and **6c** in excellent yields (95 and 97%). The *ortho*-substituted benzhydrol **6a**, however, could not be obtained via this route. Treating **2a** with *n*-BuLi led to a complex mixture containing a newly formed silicon species. While we were not successful in characterizing this species, we presume some kind of retro-Brook rearrangement to take place upon lithiating **2a**. Thus, aldehyde **5** was prepared from 1,2-benzenedimethanol (**3**) via TBS protection and subsequent benzylic oxidation with activated manganese dioxide (87% over two steps) [12]. Reaction with PhMgBr as a nucleophilic phenyl source then gave the desired benzhydrol **6a** with a 94% yield. A Steglich esterification followed by a TBAF-mediated TBS removal provided the sought for hydroxylated arecaidine esters **10a**–**c** (64–68% over two steps). In case of the *ortho* hydroxymethyl-substituted ester **10a**, multiple purification attempts, including preparative HPLC and the attempt at forming a hydrochloride salt, were insufficient to increase the compound’s purity above 95%. Hence, this compound was excluded from further biological evaluations.

The TBS-protected hydroxybenzhydrols **13a**–**c** were synthesized in a similar fashion as **6a** (Scheme 2), starting with the silylation of the corresponding hydroxybenzaldehydes **11a**–**c** (97–99%) followed by phenylation with PhMgBr (93–96%). The silyl-ether-protected hydroxybenzaldehydes **12a**–**c** were additionally reduced with sodium borohydride to produce the TBS-protected hydroxybenzyl alcohols **14a**–**c** with a 96–98% yield. The esterification of **13a**–**c** as well as **14a**–**c** employing the previously highlighted conditions smoothly furnished the arecaidine esters **15a**–**c** (78–86%) and **16a**–**c** (80–89%), respectively. While subsequent desilylation of *meta*-hydroxylated **15b** and **16b** led to the desired free alcohols **17b** (90%) and **18b** (82%), respectively, the *ortho*- and *para*-substituted derivatives decomposed upon treatment with TBAF. In accordance with our findings, Fang et al. have reported the 4-OTBS benzyl group to be a fluoride-labile protective group for carboxylic acids [13]. That the constitutional isomer 2-OTBS benzyl as well as the structurally similar 2- and 4-OTBS benzhydrol groups share the same fluoride lability is mechanistically plausible, since all of them meet the electronic requirements for a 1,4- or 1,6-elimination under liberation of an intermediary *ortho*- or *para*-quinone methide. Different attempts at synthesizing **17a**, **17c**, **18a**, and **18c**, such as buffering TBAF with acetic acid, switching to other fluoride sources, using acid-labile protective groups or foregoing the use of a protective group altogether, were not fruitful (see Scheme S1 for details).

Compound **22** was conveniently synthesized starting from commercially available 1-phenylethane-1,2-diol (**19**) in a three-step protection–esterification–deprotection sequence (42% over three steps; Scheme 3).

Compound **25** was synthesized from diol **24**, which itself was prepared from methyl benzilate (**23**) via a hydride reduction using NaBH_4_ (67% over three steps; Scheme 4). Despite being an unsymmetrical diol, **24** did not need protection for the esterification step since the steric hindrance of the benzhydrolic hydroxy group rendered it largely unreactive as compared to the primary alcohol; hence, only the desired ester was isolated.

Biphenyl ester **27** was readily obtained via Steglich esterification from commercially available [1,1′-biphenyl]-2,2′-diol (**26**) and arecaidine with a 78% yield (Scheme 5).

### 2.2. Physicochemical Properties

Considering the underlying rationale for the synthesis of the present set of hydroxylated arecaidine esters—the development of brain-penetrable mAChRs ligands for the potential application as PET probes—we decided to investigate the important physicochemical properties governing blood–brain barrier (BBB) permeability. On top of its significant role in mediating brain penetration, lipophilicity, frequently approximated by logD measurements, is an important metric in the context of PET tracer development as it allows for the preliminary assessment of the extent of NDB to be expected. While the correlation of logD and NDB is weak at best [14,15], certain hard lipophilicity cutoffs, e.g., shake-flask logD < 3.5, continue to be successfully employed in PET tracer development [16]. However, the design of brain-penetrable PET tracers displaying low NDB is a multiparameter optimization effort: relying too much on individual physicochemical parameters may be misleading since it does not give a full picture. To address this multifariousness, which is equally important in the context of classical drug design, a group of medicinal chemists around Travis Wager from Pfizer developed the CNS MPO (multiparameter optimization) score [17]. This algorithm takes into account six fundamental physicochemical properties (clogP, clogD, MW (molecular weight), tPSA (topological polar surface area), HBD (hydrogen bond donor count), and pKa) and gives a numeric output between 0 and 6.0 while avoiding hard cutoffs. The validity of the CNS MPO score was underpinned by a study correlating increasing scores with increasing experimentally determined unbound fractions in the brain [18]. Hence, we chose to use this score to assess the present compound series and compare the newly designed compounds with the parent molecule DPMA (Table 1).

Overall, the CNS MPO scores of all hydroxylated arecaidine esters fall within a narrow range of 4.83–5.75 skewed towards the upper end of the scale. According to a recent study, compounds with CNS MPO scores >3 have a lower probability of high NDB, which as such is a first indication for potential PET suitability [20]. While the parent molecule DPMA met this criterion as well and still exhibited excessive NDB in autoradiography studies, the entirety of the present compounds, in fact, had a higher score than DPMA.

Since we did not want to solely rely on the computational realm for the analysis of the synthesized compounds’ physicochemical property profiles, we experimentally approximated their lipophilicities. For this, logD values were estimated in an HPLC-based high-throughput assay employing an octadecyl-poly(vinyl alcohol) stationary phase [21]. The absence of silanol groups renders this type of column especially suited in case of analytes with hydrogen bond donor capabilities as it avoids overestimating their lipophilicity [22,23]. Notably, all compounds of this series showed a distinct decrease in HPLC-logD in comparison to its parent compound DPMA [10], with the majority falling within a range (1.2–3.1) used as a rule of thumb to develop centrally acting drugs [24]. Compounds **8a**–**c** and **22**, on the other hand, were borderline cases with HPLC-logD values of roughly one or slightly below.

Taken together, the computationally predicted CNS MPO score as well as the experimentally determined HPLC-logD render the physicochemical property profile of the hydroxylated arecaidine esters potentially suitable as imaging probes in PET applications.

### 2.3. Biological Evaluation

The arecaidine esters’ affinities towards the human mAChR subtypes *h*M_1_–*h*M_5_ were studied in a competitive radioligand binding assay using CHO-K1 cell membrane homogenates expressing a single mAChR subtype and tritiated *N*-methyl scopolamine methyl chloride ([^3^H]NMS). To speed up our affinity testing regime, we subjected all test substances to preliminary single-concentration displacement assays, as described previously [25]. Compounds with percent displacements below 75% at any of the subtypes were dropped from further investigations (Table S1). For compounds meeting the percent displacement threshold (**8c**, **10b**, **10c**, **17b**, **18b**, and **25**), inhibition constants (*K*_i_) were assessed in dose-dependent displacement experiments (Table 2).

The three benzhydrolic esters **10b**, **10c**, and **17b** displayed the highest affinities of the present set of arecaidine esters towards *h*M_1_R with *K*_i_ values in the single- and double-digit nanomolar range. Attaching a hydroxy group to one of DPMA’s phenyl rings, however, did not increase the affinity towards any of the muscarinic subtypes. Compound **17b**, the mono *meta*-hydroxylated version of DPMA, almost matched DPMA’s affinity towards *h*M_1_R but the selectivity over the other subtypes was inferior to varying degrees. While its selectivity against *h*M_2_R was, being over 30-fold, just slightly below that of DPMA, the added hydroxy group led to an almost fivefold decreased selectivity against *h*M_3_R. Compound **10c** followed the selectivity trend observed for **17b**: decreasing affinities in the order *h*M_1_R > *h*M_5_R > *h*M_4_R > *h*M_3_R > *h*M_2_R. However, the actual affinity values of the *para*-hydroxymethyl bearing compound **10c** were roughly 4–13× higher, leading to lower selectivity levels. Its *meta*-hydroxymethyl-substituted isomer **10b** followed a slightly different selectivity trend, displaying its second highest affinity towards *h*M_4_R instead of *h*M_5_R. Aligning with our previously published studies of structurally related ligands targeting mAChRs, all of the herein-evaluated compounds exhibited the highest binding constant for *h*M_2_R. The selectivity trend of **18b** was especially noteworthy—mentally detaching one phenyl substituent from **17b** and arriving at **18b** leads to a unique to this series, significant *h*M_5_-preferrring tendency which considerably differs from **17b**’s selectivity profile.

**Figure 2:**
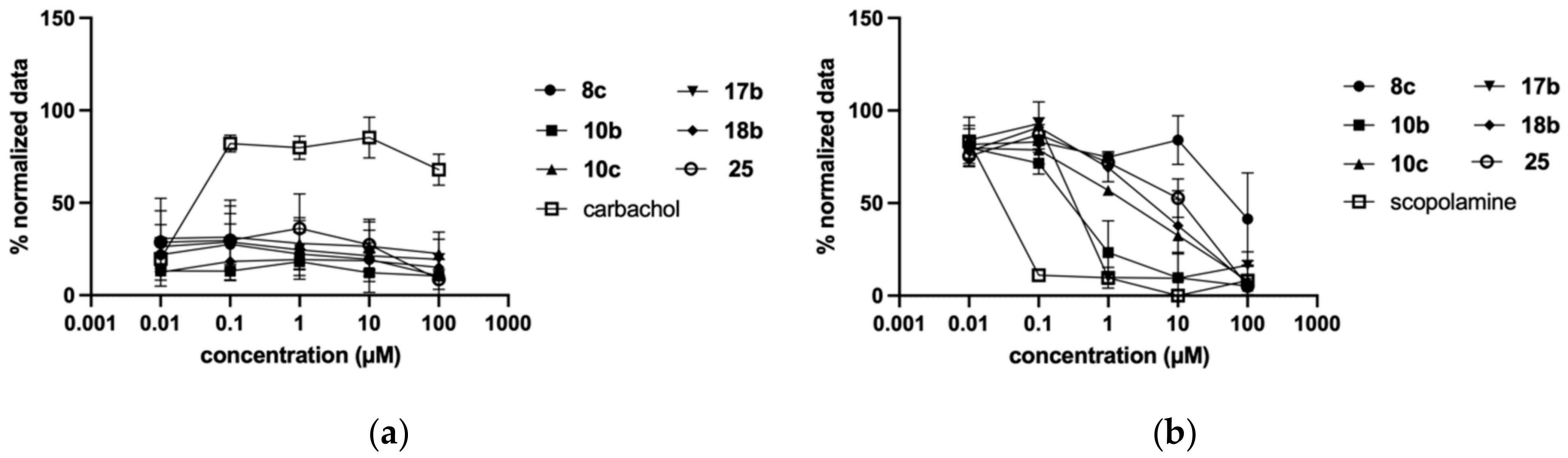
Dose-dependent Ca^2+^ flux induced by a set of hydroxylated arecaidine esters in CHO-*h*M_1_ cells. (**a**) Agonist dose–response experiment; (**b**) antagonist dose–response experiment with the reference agonist carbachol added at a final concentration of 20 µM.

As a prerequisite for our planned functionality testing efforts of the herein-presented hydroxylated arecaidine esters, we assayed the compounds’ cell viability in living CHO-*h*M_1_ cells using MTT. Gratifyingly, we found cytotoxicity to be of no concern for our purposes with the corresponding IC_50_ values being in the double-digit micromolar range and above (Figure S1). Having ruled out any interference by the esters’ hypothetical cytotoxicity, we sought to explore the functionality of the synthesized arecaidine esters towards the muscarinic receptor subtype M_1_ by discriminating between agonistic and antagonistic behavior. As reported previously [11], by using Fluo-4, the calcium mobilization in CHO-*h*M_1_ cells was assayed in response to the stimulation with the hydroxylated arecaidine esters. At none of the assayed concentrations did any of the tested esters induce a significant calcium flux, as can be observed from known agonist carbachol (Figure 2a and Figure S2a). Yet, all of them were able to inhibit a carbachol-induced flux in a similar fashion as the known antagonist scopolamine (Figure 2b and Figure S2b), thereby indicating the antagonistic binding of the entire set of assayed compounds.

### 2.4. Molecular Docking Studies

**Figure 3:**
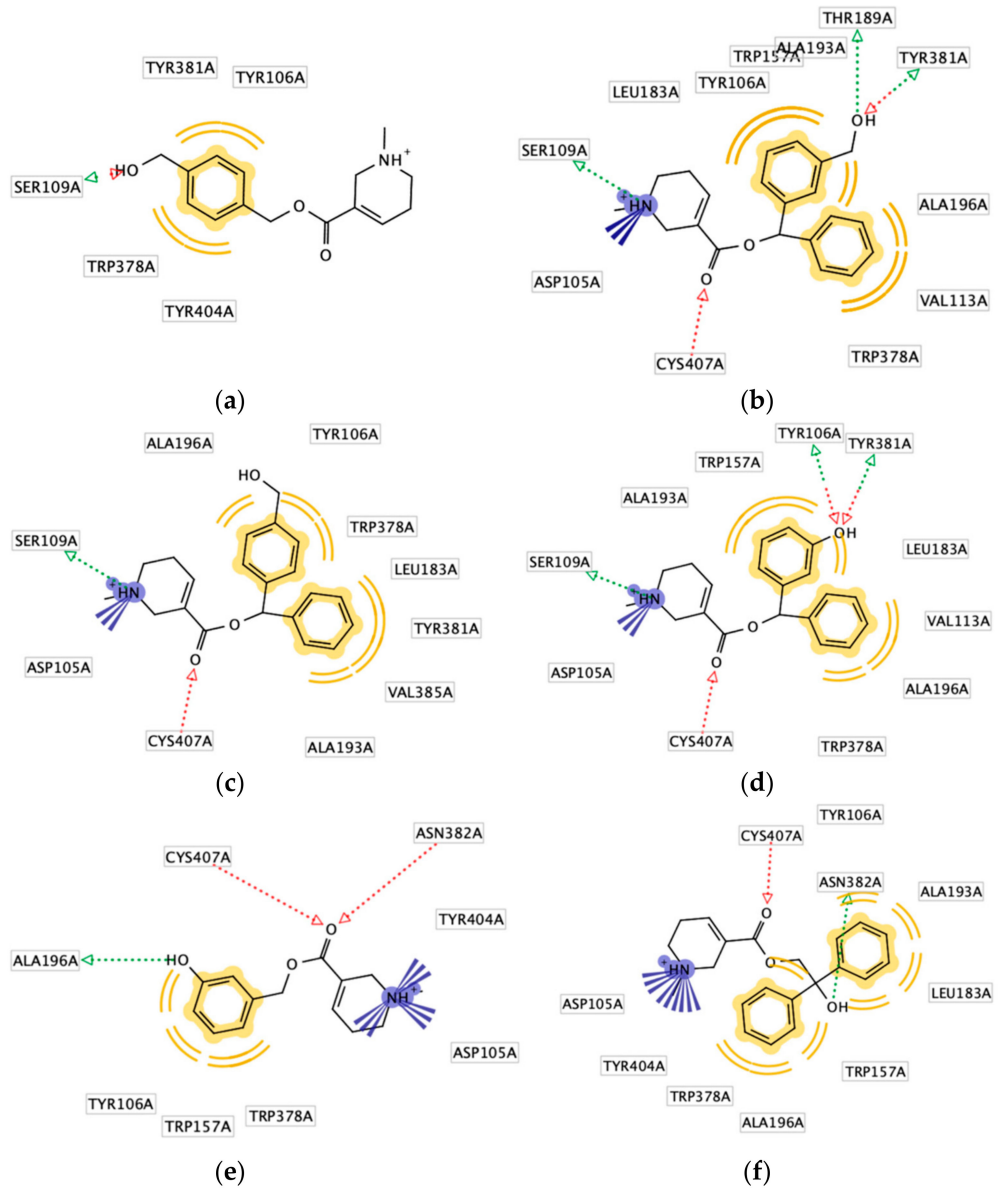
Two-dimensional pharmacophores for selected hydroxylated arecaidine esters in the orthosteric binding site of M_1_ (PDB 5CXV) with interacting amino acid residues and key interactions highlighted: (**a**) 2D pharmacophore of **8c**; (**b**) 2D pharmacophore of (*R*)-**10b**; (**c**) 2D pharmacophore of (*R*)-**10c**; (**d**) 2D pharmacophore of (*R*)-**17b**; (**e**) 2D pharmacophore of **18b**; (**f**) 2D pharmacophore of **25**.

We performed molecular docking studies to better our understanding of the observed binding affinities in atomic detail. Due to the high degree of sequence homology between the muscarinic subtypes, docking alone frequently does not meet the requirements to predict/explain subtype selectivity, hence the herein-presented docking studies are focused on giving qualitative insights into the compounds’ binding mode against the M_1_ muscarinic receptor structure (PDB 5CXV). As a first step, for each of the docked ligands a representative pose was chosen among the nine modes returned. Hence, following our previously presented approach, in each case we selected the highest ranked pose featuring a salt bridge between the positively charged *N*-methyl amine and the—among aminergic GPCRs-conserved—aspartic acid residue Asp105^3.32^ (superscript numerals refer to the Ballesteros–Weinstein numbering scheme for GPCRs [26,27]). Since none of the predicted modes of ester **8c** showed the aforementioned interaction, simply the highest ranked pose was chosen. The 2D pharmacophores corresponding to the six fully, biologically evaluated ligands **8c**, **10b**, **10c**, **17b**, **18b**, and **25** are highlighted in Figure 3, while those corresponding to the ligands **10a**, **17a**, **17c**, **18a**, and **18c** whose syntheses were not successful are shown in Figure S3.

**Figure 4:**
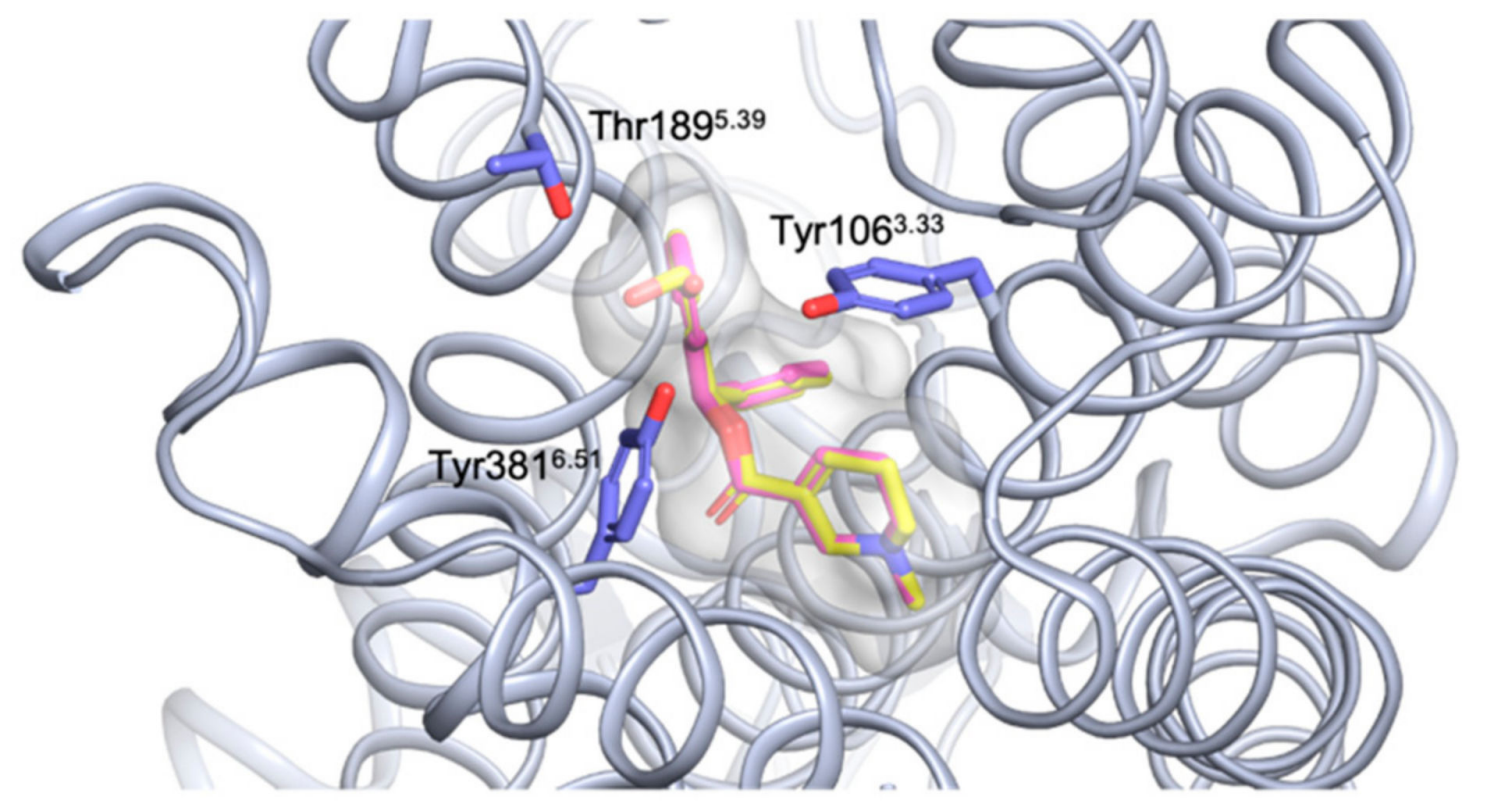
Overlay of the representative docking poses of (*R*)-**10b** (carbons in yellow) and (*R*)-**17b** (carbons in magenta) in the orthosteric binding site of M_1_ (PDB 5CXV). Key amino acid residues which engage in hydrogen bonding with the hydroxy group of the depicted ligands are highlighted.

Unlike benzylic ester **8c**, which is predicted to adopt a pose merely engaging in hydrogen bonding with Ser109^3.36^ paired with phenyl-based hydrophobic interactions (Figure 3a), the cationic head of its closely structurally related ester **18b** is anchored in proximity to Asp105^3.22^ (Figure 3e). However, the positively charged group also captures interactions with Tyr404^7.39^ and Trp378^6.48^—a feature which is shared with compound **25** (Figure 3f). In addition, the carbonyl oxygen of **18b** serves as a hydrogen bond acceptor for both Cys407^7.42^ and Asn382^6.52^. Noteworthy, with the exception of **8b**, the hydrogen bonding interaction with Cys407^7.42^ is shared among all docked compounds, and has been observed in previous studies with structurally similar compounds [10,11,25]. Taken together, the sum of these pharmacophoric features explains **18b**’s higher affinity towards *h*M_1_R compared to **8c**. Compound **25** also engages in hydrogen bonding with Asn382^6.52^, however, it does so via its hydroxy group, which serves as a hydrogen bond donor (Figure 3f). The benzhydrolic esters **10b**, **10c**, and **17b** are of particular interest since they showed the highest affinities of all compounds of this study towards *h*M_1_R. Importantly, these compounds bear a chiral center, and for the sake of simplicity only the (*R*)-enantiomers’ 2D pharmacophores are depicted in Figure 3b–d; for a depiction of the (*S*)-enantiomers see Figure S4). Evidently, the interactions with the orthosteric binding site for the arecaidine-part of the molecules are predicted to be identical, which holds true for the (*S*)-enantiomers as well—with the exception that the ammonium groups of (*S*)-**10b** and (*S*)-**17b** not only interact with Asp105^3.32^ but also with Tyr404^7.39^ (Figure S5). The two phenyl rings fill two lipophilic pockets, hydrophobically engaging with similar amino acid residues as are characteristic for the receptor’s cocrystallized inverse agonist tiotropium [28]. Neither the (*R*)- nor the (*S*)-enantiomer of **10c** exhibits noteworthy interactions between its hydroxy groups and the receptor, which might explain the somewhat lower binding affinity towards *h*M_1_R compared to **10b** and **17b**. The phenolic hydroxy group of **17b** and the benzylic hydroxy group of **10b**, however, act both as a hydrogen bond acceptor and donor towards different amino acid residues (Figure 3b,d). Overlaying the poses of these two compounds reveals their very close resemblance (Figure 4). In fact, merely the two hydroxy groups adapt slightly different positions and as a result, they both feature a mutual hydrogen bonding interaction with Tyr381^6.51^ as well as separate interactions with Tyr106^3.33^ and Thr189^5.39^, respectively. The hydroxy groups of the corresponding (*S*)-enantiomers point in the opposite lipophilic pocket. While the polarized hydrogen of the hydroxymethyl group of (*S*)-**10b** reaches the backbone carbonyl oxygen of Tyr106^3.33^ and subsequently undergoes a hydrogen bond, (*S*)-**17b**’s phenolic hydroxy group falls short of capturing any meaningful interactions.

**Figure 5:**
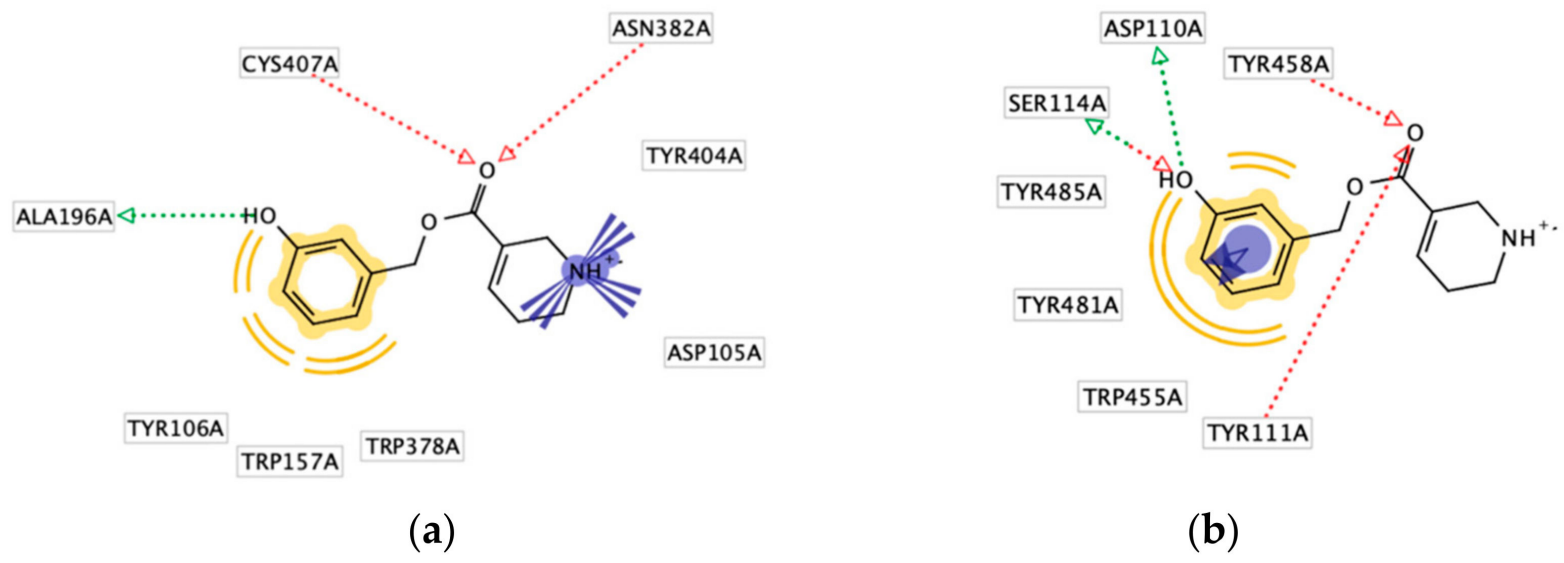
Comparison of the 2D pharmacophores of **18b** in the orthosteric binding site of M_1_ (PDB 5CXV) and M_5_ (PDB 6OL9) with interacting amino acid residues and key interactions highlighted: (**a**) 2D pharmacophore of **18b** in the ortho-steric binding site of M_1_; (**b**) 2D pharmacophore of **18b** in the orthosteric binding site of M_5_.

Due to the fact that **18b** was the only compound of this series showing a significant preference for the *h*M_5_R in affinity evaluations, we felt compelled to compare this ligand’s interactions with the orthosteric binding site of M_1_ and M_5_. A side-by-side comparison of the 2D representations of the key interactions corresponding to the highest ranked docking pose in each case is shown in Figure 5. The interactions with the binding site of M_1_ (Figure 5a) have mostly been discussed above—with the difference that **18b** is only able to fill one of the binding site’s lipophilic pockets and its hydrogen-bond-donating role towards Ala196^5.46^, its binding mode shows close resemblance with that of tiotropium. As opposed to this, none of the nine sampled binding modes of **18b** feature an ionic interaction between the ester’s cationic head and the aspartic acid residue Asp110^3.32^ in the binding site of M_5_. Instead, the highest ranked pose is predicted to adopt a conformation in which its phenyl ring is placed such that the phenolic hydroxy group engages in hydrogen-bonding with Asp110^3.32^ and Ser114^3.36^. The tetrahydropyridine-part of the molecule, on the other hand, does not seem to capture any meaningful interactions with the receptor. While this docking experiment gives first insights into the differing interactions in the M_1_ and M_5_ orthosteric binding site, it does not explain the almost threefold preference of **18b** for *h*M_5_R over *h*M_1_R in in vitro experiments. For this, more sophisticated computational studies will be necessary.

## 3. Materials and Methods

### 3.1. Chemistry

#### 3.1.1. General Considerations

Unless otherwise stated, all reagents were purchased from commercial suppliers and used as received without further purification. All reactions were conducted under an inert atmosphere of argon, and commercially available anhydrous solvents were used. Flash column chromatography was performed on a Biotage^®^ Selekt Flash Chromatography System equipped with Biotage^®^ Sfär HC cartridges using either HPLC-grade or reagent-grade solvents. Reactions were monitored by TLC on precoated aluminum sheets (Polygram SIL G/UV254, 0.2 mm, with fluorescent indicator; Macherey-Nagel, Düren, Germany); the spots were visualized under UV light (λ = 254 nm) and/or KMnO_4_ stain. ^1^H and ^13^C NMR spectra were recorded in deuterated chloroform (CDCl_3_) at 298 K on a Bruker Avance III 400 or Bruker Avance III 500 spectrometer and are reported as follows: chemical shift δ in ppm (multiplicity, coupling constant *J* in Hz, number of protons, assignment) for ^1^H NMR spectra and chemical shift δ in ppm (assignment) for ^13^C spectra. For ^1^H and ^13^C NMR spectra, the residual solvent peaks of CDCl_3_ (δ_H_ = 7.26 ppm, δ_C_ = 77.00 ppm) were used as internal reference. The chemical shifts of all signals are reported as the center of the resonance range. Unless stated otherwise, a full and unambiguous assignment of all resonances was performed by a combination of standard NMR techniques, such as APT, HSQC, HMBC, COSY, and NOESY experiments. IR spectra were recorded on a Bruker Alpha II FTIR spectrometer. Samples were prepared as a film by evaporation of a solution in CH_2_Cl_2_ or CDCl_3_ and selected absorption bands are reported in wavenumbers (cm^−1^). HRMS spectra were recorded on a Bruker maXis 4G instrument (ESI-TOF). Melting points were measured with an Electrothermal IA9200 melting point apparatus in open glass capillaries and are uncorrected. All biologically tested compounds exhibited ≥95% purity under the HPLC conditions reported hereafter. HPLC analyses were performed using a Shimadzu HPLC system consisting of a degassing unit (DGU-20A3R), a liquid chromatograph (LC-20ADXR), an autosampler (SIL-20ACHT), a diode array detector (SPD-M20A), a column oven (CTO-20AC) and a communication bus module (CBM-20A). An Eclipse Plus column (4.6 × 100 mm, 3.5 µm, Agilent, Santa Clara, CA, USA) and a gradient consisting of the following components were used: solvent A: 0.1% TFA in double distilled water; solvent B: 0.1% TFA in acetonitrile. Purities were measured with a gradient run by increasing solvent B from 10 to 100% within 9.4 min with a flow rate of 1.5 mL/min as well as with an isocratic run (Figures S6–S15). Intermediates **2a**−**b** [29,30], **4** [12], **5** [12], **6b** [31], **12a**−**c** [32,33,34], **13a** [32], **13c** [35], **14a**−**c** [32,34,36], **20** [37], and **24** [38] have been described previously, and were synthesized following the steps and conditions outlined in Scheme 1, Scheme 2, Scheme 3 and Scheme 4. Arecaidine was synthesized according to a previously reported procedure [39].

#### 3.1.2. Synthetic Procedures for Precursors (**6a**, **6c**, **13b**)

(2-(((*tert*-butyldimethylsilyl)oxy)methyl)phenyl)(phenyl)methanol (**6a**). To an ice-cooled solution of aldehyde **5** (300 mg, 1.20 mmol, 1.0 equiv) in anhydrous THF (6 mL) was added dropwise a solution of PhMgBr (1.0 M in THF, 1.3 mL, 1.1 equiv) over a period of 10 min. It was allowed to warm slowly to ambient temperature and was stirred overnight. Then, the mixture was quenched by the addition of water and was extracted with EtOAc (3×). The combined organic layers were dried (Na_2_SO_4_) and concentrated under reduced pressure. Purification by flash column chromatography (0–20% EtOAc in *n*-heptane) afforded the title compound **6a** (368 mg, 94%) as a colorless oil. ^1^H NMR (500 MHz, CDCl_3_) δ 7.39 (m, 2H, Ph H-2′,6′), 7.34 (m, 3H, Ph H-5, Ph H-3′,5′), 7.31 (m, 1H, Ph H-3), 7.28 (m, 1H, Ph H-4), 7.27 (m, 1H, Ph H-4′), 7.26 (m, 1H, Ph H-6), 6.03 (s, 1H, CHPh_2_), 4.74 (d, *J* = 12.2 Hz, 1H, CH_2_), 4.53 (d, *J* = 12.2 Hz, 1H, CH_2_), 0.91 (s, 9H, C(CH_3_)_3_), 0.09 (s, 3H, Si(CH_3_)_2_), 0.08 (s, 3H, Si(CH_3_)_2_). ^13^C NMR (125 MHz, CDCl_3_) δ 142.8 (Ph C-1′), 142.7 (Ph C-2), 138.0 (Ph C-1), 129.2 (Ph C-3), 128.7 (Ph C-6), 128.2 (Ph C-5, Ph C-3′,5′), 127.8 (Ph C-4), 127.1 (Ph C-4′), 126.5 (Ph C-2′,6′), 74.0 (CHPh_2_), 64.5 (CH_2_), 25.9 (C(CH_3_)_3_), 18.3 (C(CH_3_)_3_), −5.28 (Si(CH_3_)_2_), −5.31 (Si(CH_3_)_2_). IR (film) *ν*_max_ 2929, 1454, 1255, 1114, 1035, 836, 777, 699. HRMS (ESI) (m/z) calcd for C_20_H_28_NaO_2_Si [M + Na]^+^: 351.1751; found 351.1750.

(4-(((*tert*-butyldimethylsilyl)oxy)methyl)phenyl)(phenyl)methanol (**6c**). To a solution of aryl bromide **2b** (362 mg, 1.20 mmol, 1.0 equiv) in anhydrous THF (6 mL) was added dropwise a solution of *n*-BuLi (2.5 M in hexanes, 0.48 mL, 1.0 equiv) over a period of 10 min at −78 °C. The reaction mixture was stirred for 30 min at this temperature. After dropwise addition of benzaldehyde (191 mg, 1.80 mmol, 1.5 equiv) the resulting solution was allowed to warm to ambient temperature and stirred for 6 h. Then, the reaction was quenched by the addition of sat. aq. NH_4_Cl and was extracted with EtOAc (3×). The combined organic layers were dried (Na_2_SO_4_) and concentrated under reduced pressure. Purification by flash column chromatography (0–20% EtOAc in *n*-heptane) afforded the title compound **6c** (360 mg, 92%) as a colorless oil. ^1^H NMR (500 MHz, CDCl_3_) δ 7.29 (m, 2H, Ph H-2,6), 7.25 (m, 2H, Ph H-3′,5′), 7.24 (m, 2H, Ph H-2′,6′), 7.21 (m, 2H, Ph H-3,5), 7.17 (m, 1H, Ph H-4′), 5.74 (s, 1H, CHPh_2_), 4.63 (s, 2H, CH_2_), 0.85 (s, 9H, C(CH_3_)_3_), 0.0 (s, 6H, Si(CH_3_)_2_). ^13^C NMR (125 MHz, CDCl_3_) δ 143.8 (Ph C-1′), 142.5 (Ph C-1), 140.8 (Ph C-4), 128.4 (Ph C-3′,5′), 127.5 (Ph C-4′), 126.48 (Ph C-2′,6′), 126.44 (Ph C-2,6), 126.2 (Ph C-3,5), 74.7 (CHPh_2_), 64.7 (CH_2_), 25.9 (C(CH_3_)_3_), 18.4 (C(CH_3_)_3_), −5.3 (Si(CH_3_)_2_). IR (film) *ν*_max_ 2929, 1255, 1080, 837, 776, 699. HRMS (ESI) (m/z) calcd for C_20_H_28_NaO_2_Si [M + Na]^+^: 351.1751; found 351.1751.

(3-((*tert*-butyldimethylsilyl)oxy)phenyl)(phenyl)methanol (**13b**). To an ice-cooled solution of aldehyde **12b** (284 mg, 1.20 mmol, 1.0 equiv) in anhydrous THF (6 mL) was added dropwise a solution of PhMgBr (1.0 M in THF, 1.3 mL, 1.1 equiv) over a period of 10 min. It was allowed to warm slowly to ambient temperature and was stirred overnight. Then, the mixture was quenched by the addition of water and was extracted with EtOAc (3×). The combined organic layers were dried (Na_2_SO_4_) and concentrated under reduced pressure. Purification by flash column chromatography (0–20% EtOAc in *n*-heptane) afforded the title compound **13b** (360 mg, 96%) as a colorless oil. ^1^H NMR (500 MHz, CDCl_3_) δ 7.38 (m, 2H, Ph H-2′,6′), 7.34 (m, 2H, Ph H-3′,5′), 7.28 (m, 1H, Ph H-4′), 7.19 (m, 1H, Ph H-5), 6.95 (m, 1H, Ph H-6), 6.87 (m, 1H, Ph H-2), 6.74 (m, 1H, Ph H-4), 5.79 (s, 1H, CHPh_2_), 2.19 (br s, 1H, OH), 0.96 (s, 9H, C(CH_3_)_3_), 0.16 (s, 6H, Si(CH_3_)_2_). ^13^C NMR (125 MHz, CDCl_3_) δ 155.8 (Ph-3), 145.4 (Ph C-1), 143.7 (Ph C-1′), 129.4 (Ph C-5), 128.4 (Ph C-3′,5′), 127.5 (Ph C-4′), 126.5 (Ph C-2′,6′), 119.4 (Ph C-6), 119.1 (Ph C-4), 76.0 (CHPh_2_), 25.7 (C(CH_3_)_3_), 18.2 (C(CH_3_)_3_), −4.5 (Si(CH_3_)_2_). IR (film) *ν*_max_ 2929, 2858, 1600, 1485, 1254, 1149, 1003, 963, 838, 781, 699. HRMS (ESI) (m/z) calcd for C_19_H_26_NaO_2_Si [M + Na]^+^: 337.1600; found 337.1597.

#### 3.1.3. General Procedure for the EDC-Mediated Esterification of Arecaidine (**8a**−**c**, **9a**−**c**, **15a**−**c**, **16a**−**c**, **21**, **25**, **27**)

To a heterogeneous mixture of arecaidine (1.0 equiv) and alcohol (1.5 equiv) in anhydrous CH_2_Cl_2_ (0.5 M) were added EDC-HCl (2.0 equiv) and 4-DMAP (0.5 equiv). The resulting mixture was vigorously stirred for 2 d. Then, the turbid mixture was poured into sat. aq. NaHCO_3_ and extracted with CH_2_Cl_2_ (3×). The combined organic layers were dried (Na_2_SO_4_) and concentrated under reduced pressure. The crude residue was purified by flash column chromatography to give the desired ester.

2-(hydroxymethyl)benzyl 1-methyl-1,2,5,6-tetrahydropyridine-3-carboxylate (**8a**). Following the general procedure on a 0.25 mmol scale, arecaidine was esterified using 1,2-benzenedimethanol (**7a**). Purification by flash column chromatography (0–12% MeOH in CH_2_Cl_2_) afforded the title compound **8a** (51 mg, 78%) as a pale orange solid. mp 75–77 °C. ^1^H NMR (400 MHz, CDCl_3_) δ 7.42 (m, 1H, Ph H-3), 7.37 (m, 1H, Ph H-6), 7.33 (m, 1H, Ph H-4), 7.29 (m, 1H, Ph H-5), 7.01 (m, 1H, H-4) 5.27 (s, 2H, (CO)OCH_2_), 4.72 (s, 2H, CH_2_OH), 3.3–2.9 (br s, 1H, OH), 3.12 (m, 2H, H-2), 2.48 (t, *J* = 5.6 Hz, 3H, H-6), 2.37 (s, 3H, NCH_3_), 2.35 (m, 2H, H-5). ^13^C NMR (100 MHz, CDCl_3_) δ 165.4 (C=O), 139.4 (Ph C-2), 138.2 (C-4), 133.7 (Ph C-1), 129.4 (Ph C-6), 128.7 (Ph C-34), 128.4 (Ph C-3), 128.6 (C-3), 127.9 (Ph C-5), 63.8 ((CO)OCH_2_), 62.6 (CH_2_OH), 53.0 (C-2), 50.6 (C-6), 45.5 (NCH_3_), 26.5 (C-5). IR (film) *ν*_max_ 2943, 1709, 1454, 1398, 1290, 1263, 1139, 1087, 1046, 1026, 759. HRMS (ESI) (m/z) calcd for C_15_H_20_NO_3_ [M + H]^+^: 262.1438; found 262.1445.

3-(hydroxymethyl)benzyl 1-methyl-1,2,5,6-tetrahydropyridine-3-carboxylate (**8b**). Following the general procedure on a 0.25 mmol scale, arecaidine was esterified using 1,3-benzenedimethanol (**7b**). Purification by flash column chromatography (0–12% MeOH in CH_2_Cl_2_) afforded the title compound **8b** (54 mg, 83%) as a yellow oil. ^1^H NMR (400 MHz, CDCl_3_) δ 7.32 (m, 1H, Ph H-2), 7.31 (m, 1H, Ph H-5), 7.29 (m, 1H, Ph H-4), 7.24 (m, 1H, Ph H-6), 7.01 (m, 1H, H-4), 5.14 (s, 2H, (CO)OCH_2_), 4.64 (s, 3H, CH_2_OH), 3.26 (br s, 1H, OH), 3.12 (m, 2H, H-2), 2.47 (t, *J* = 5.6 Hz, 3H, H-6), 2.36 (s, 3H, NCH_3_), 2.34 (m, 2H, H-5). ^13^C NMR (100 MHz, CDCl_3_) δ 165.4 (C=O), 141.7 (Ph C-3), 138.0 (C-4), 136.2 (Ph C-1), 128.6 (Ph C-5), 128.5 (C-3), 127.0 (Ph C-6), 126.6 (Ph C-4), 126.4 (Ph C-2), 66.0 ((CO)OCH_2_), 64.7 (CH_2_OH), 52.9 (C-2), 50.6 (C-6), 45.5 (NCH_3_), 26.3 (C-5). ^1^IR (film) *ν*_max_ 1706, 1450, 1290, 1261, 1138, 1129, 1088, 1046, 1026, 999, 790, 719, 700. HRMS (ESI) (m/z) calcd for C_15_H_20_NO_3_ [M + H]^+^: 262.1438; found 262.1434.

4-(hydroxymethyl)benzyl 1-methyl-1,2,5,6-tetrahydropyridine-3-carboxylate (**8c**). Following the general procedure on a 0.25 mmol scale, arecaidine was esterified using 1,4-benzenedimethanol (**7c**). Purification by flash column chromatography (0–12% MeOH in CH_2_Cl_2_) afforded the title compound **8c** (48 mg, 73%) as a pale orange semi-solid. ^1^H NMR (400 MHz, CDCl_3_) δ 7.32 (m, 2H, Ph H-3,5), 7.31 (m, 2H, Ph H-2,6), 7.01 (m, 1H, H-4), 5.14 (s, 2H, (CO)OCH_2_), 4.64 (s, 2H, CH_2_OH), 3.26 (br s, 1H, OH), 3.12 (m, 2H, H-2), 2.47 (t, *J* = 5.7 Hz, 3H, H-6), 2.36 (s, 3H, NCH_3_), 2.34 (m, 2H, H-5). ^13^C NMR (100 MHz, CDCl_3_) δ 165.4 (C=O), 141.3 (Ph C-4), 138.0 (C-4), 135.1 (Ph C-1), 128.6 (C-3), 128.2 (Ph-2,6), 126.9 (Ph C-3,5), 65.9 ((CO)OCH_2_), 64.6 (CH_2_OH), 52.9 (C-2), 50.6 (C-6), 45.5 (NCH_3_), 26.3 (C-5). IR (film) *ν*_max_ 1705, 1654, 1459, 1398, 1259, 1128, 1087, 1018, 790, 717. HRMS (ESI) (m/z) calcd for C_15_H_20_NO_3_ [M + H]^+^: 262.1438; found 262.1435.

(2-(((*tert*-butyldimethylsilyl)oxy)methyl)phenyl)(phenyl)methyl 1-methyl-1,2,5,6-tetrahydropyridine-3-carboxylate (**9a**). Following the general procedure on a 0.25 mmol scale, arecaidine was esterified using **6a**. Purification by flash column chromatography (0–6% MeOH in CH_2_Cl_2_) afforded the title compound **9a** (82 mg, 73%) as a yellow oil. ^1^H NMR (400 MHz, CDCl_3_) δ 7.51 (m, 1H, Ph H-3), 7.41 (m, 1H, Ph H-6), 7.33 (m, 1H, Ph H-4), 7.30 (m, 5H, Ph H-2′,3′,4′,5′,6′), 7.29 (m, 1H, Ph H-5), 7.17 (s, 1H, CHPh_2_), 7.14 (m, 1H, H-4), 4.80 (d, *J* = 13.3 Hz, 1H, CH_2_OSi), 4.73 (d, *J* = 13.3 Hz, 1H, CH_2_OSi), 3.30 (m, 2H, H-2), 2.61 (m, 2H, H-6), 2.49 (s, 3H, NCH_3_), 2.47 (m, 2H, H-5), 0.90 (s, 9H, C(CH_3_)_3_), −0.03 (s, 3H, SiCH_3_), −0.04 (s, 3H, SiCH_3_). ^13^C NMR (100 MHz, CDCl_3_) δ 164.5 (C=O), 139.6 (Ph C-1′), 138.9 (Ph C-2), 138.2 (C-4), 137.0 (Ph C-1), 128.9 (C-3), 128.3 (Ph C-3′,5′), 128.1 (Ph C-4), 127.8 (Ph C-4′), 127.4 (Ph C-6), 127.3 (Ph C-5; Ph C-2′,6′), 127.0 (Ph C-3), 73.5 (CHPh_2_), 62.7 (CH_2_OSi), 53.2 (C-2), 50.8 (C-6), 45.7 (NCH_3_), 26.7 (C-5), 25.9 (C(CH_3_)_3_), 18.4 (C(CH_3_)_3_), −5.38 (SiCH_3_), −5.40 (SiCH_3_). IR (film) *ν*_max_ 2928, 1710, 1462, 1251, 1189, 1140, 1115, 1075, 1024, 998, 970, 834, 775, 757, 718, 697. HRMS (ESI) (m/z) calcd for C_27_H_38_NO_3_Si [M + H]^+^: 452.2615; found 452.2621.

(3-(((*tert*-butyldimethylsilyl)oxy)methyl)phenyl)(phenyl)methyl 1-methyl-1,2,5,6-tetrahydropyridine-3-carboxylate (**9b**). Following the general procedure on a 0.25 mmol scale, arecaidine was esterified using **6b**. Purification by flash column chromatography (0–6% MeOH in CH_2_Cl_2_) afforded the title compound **9b** (89 mg, 79%) as a yellow oil. ^1^H NMR (400 MHz, CDCl_3_) δ 7.35 (m, 2H, Ph H-2′,6′), 7.33 (m, 2H, Ph H-3′,5′), 7.32 (m, 1H, Ph H-2), 7.29 (m, 1H, Ph H-5), 7.27 (m, 1H, Ph H-4′), 7.24 (m, 2H, Ph H-4,6), 7.16 (m, 1H, H-4), 6.94 (s, 1H, CHPh_2_), 4.72 (s, 2H, CH_2_OSi), 3.29 (m, 2H, H-2), 2.60 (m, 2H, H-6), 2.48 (s, 3H, NCH_3_), 2.46 (m, 2H, H-5), 0.90 (s, 9H, C(CH_3_)_3_), 0.06 (s, 6H, Si(CH_3_)_2_). ^13^C NMR (100 MHz, CDCl_3_) δ 164.6 (C=O), 141.8 (Ph C-3), 140.4 (Ph C-1), 140.3 (Ph C-1′), 138.2 (C-4), 129.0 (C-3), 128.5 (Ph C-3′,5′), 128.4 (Ph C-5), 127.8 (Ph C-4′), 127.1 (Ph C-2′,6′), 125.7 (Ph C-4,6), 124.6 (Ph C-2), 76.7 (CHPh_2_), 64.8 (CH_2_OSi), 53.2 (C-2), 50.8 (C-6), 45.7 (NCH_3_), 26.7 (C-5), 26.0 (C(CH_3_)_3_), 18.4 (C(CH_3_)_3_), −5.3 (Si(CH_3_)_2_). IR (film) *ν*_max_ 2928, 1711, 1252, 1140, 1081, 1025, 836, 777, 700. HRMS (ESI) (m/z) calcd for C_27_H_38_NO_3_Si [M + H]^+^: 452.2615; found 452.2624.

(4-(((*tert*-butyldimethylsilyl)oxy)methyl)phenyl)(phenyl)methyl 1-methyl-1,2,5,6-tetrahydropyridine-3-carboxylate (**9c**). Following the general procedure on a 0.25 mmol scale, arecaidine was esterified using **6c**. Purification by flash column chromatography (0–6% MeOH in CH_2_Cl_2_) afforded the title compound **9c** (79 mg, 70%) as a yellow oil. ^1^H NMR (400 MHz, CDCl_3_) δ 7.34 (m, 2H, Ph H-2′,6′), 7.33 (m, 2H, Ph H-3′,5′), 7.32 (m, 2H, Ph H-2,6), 7.31 (m, 2H, Ph H-3,5), 7.27 (m, 1H, Ph H-4′), 7.18 (m, 1H, H-4), 6.93 (s, 1H, CHPh_2_), 4.72 (s, 2H, CH_2_OSi), 3.34 (m, 2H, H-2), 2.64 (m, 2H, H-6), 2.51 (s, 3H, NCH_3_), 2.48 (m, 2H, H-5), 0.93 (s, 9H, C(CH_3_)_3_), 0.09 (s, 6H, Si(CH_3_)_2_). ^13^C NMR (100 MHz, CDCl_3_) δ 164.7 (C=O), 141.2 (Ph C-4), 140.4 (Ph C-1′), 138.9 (Ph C-1), 138.2 (C-4), 129.0 (C-3), 128.5 (Ph C-3′,5′), 127.8 (Ph C-4′), 127.0 (Ph C-2,6; Ph C-2′,6′), 126.1 (Ph C-3,5), 76.5 (CHPh_2_), 64.6 (CH_2_OSi), 53.2 (C-2), 50.8 (C-6), 45.7 (NCH_3_), 26.7 (C-5), 26.0 (C(CH_3_)_3_), 18.4 (C(CH_3_)_3_), −5.3 (Si(CH_3_)_2_). IR (film) *ν*_max_ 2928, 1711, 1252, 1140, 1084, 1024, 837, 776, 698. HRMS (ESI) (m/z) calcd for C_27_H_38_NO_3_Si [M + H]^+^: 452.2615; found 452.2625.

(2-((*tert*-butyldimethylsilyl)oxy)phenyl)(phenyl)methyl 1-methyl-1,2,5,6-tetrahydropyridine-3-carboxylate (**15a**). Following the general procedure on a 0.25 mmol scale, arecaidine was esterified using **13a**. Purification by flash column chromatography (0–6% MeOH in CH_2_Cl_2_) afforded the title compound **15a** (85 mg, 78%) as a yellow oil. ^1^H NMR (400 MHz, CDCl_3_) δ 7.38 (dd, *J* = 7.7, 1.8 Hz, 1H, Ph H-6), 7.34 (s, 1H, CHPh_2_), 7.32 (m, 2H, Ph H-2′,6′), 7.30 (m, 2H, Ph H-3′,5′), 7.24 (m, 1H, Ph H-4′), 7.18 (m, 1H, Ph H-4), 7.14 (m, 1H, H-4), 6.96 (m, 1H, Ph H-5), 6.84 (dd, *J* = 8.1, 1.1 Hz, 1H, Ph H-3), 3.22 (m, 2H, H-2), 2.50 (m, 2H, H-6), 2.41 (s, 3H, NCH_3_), 2.38 (m, 2H, H-5), 0.97 (s, 9H, C(CH_3_)_3_), 0.29 (s, 3H, SiCH_3_), 0.21 (s, 3H, SiCH_3_). ^13^C NMR (100 MHz, CDCl_3_) δ 164.4 (C=O), 152.7 (Ph C-2), 140.2 (Ph C-1′), 137.7 (C-4), 130.7 (Ph C-1), 129.0 (C-3), 128.6 (Ph C-4), 128.2 (Ph C-3′,5′), 127.7 (Ph C-6), 127.5 (Ph C-4′), 127.0 (Ph C-2′,6′), 121.0 (Ph C-5), 118.4 (Ph C-3), 71.4 (CHPh_2_), 53.1 (C-2), 50.7 (C-6), 45.6 (NCH_3_), 26.5 (C-5), 25.7 (C(CH_3_)_3_), 18.2 (C(CH_3_)_3_), −4.13 (SiCH_3_), −4.18 (SiCH_3_). IR (film) *ν*_max_ 2930, 1713, 1489, 1454, 1254, 1141, 1025, 921, 839, 783, 697. HRMS (ESI) (m/z) calcd for C_26_H_36_NO_3_Si [M + H]^+^: 438.2459; found 438.2461.

(3-((*tert*-butyldimethylsilyl)oxy)phenyl)(phenyl)methyl 1-methyl-1,2,5,6-tetrahydropyridine-3-carboxylate (**15b**). Following the general procedure on a 0.25 mmol scale, arecaidine was esterified using **13b**. Purification by flash column chromatography (0–6% MeOH in CH_2_Cl_2_) afforded the title compound **15b** (92 mg, 84%) as a yellow oil. ^1^H NMR (400 MHz, CDCl_3_) δ 7.34 (m, 2H, Ph H-2′,6′), 7.33 (m, 2H, Ph H-3′,5′), 7.27 (m, 1H, Ph H-4′), 7.19 (m, 1H, Ph H-5), 7.15 (m, 1H, H-4), 6.93 (m, 1H, Ph H-6), 6.89 (s, 1H, CHPh_2_), 6.84 (m, 1H, Ph H-2), 6.76 (m, 1H, Ph H-4), 3.22 (m, 2H, H-2), 2.51 (m, 2H, H-6), 2.43 (s, 3H, NCH_3_), 2.40 (m, 2H, H-5), 0.97 (s, 9H, C(CH_3_)_3_), 0.17 (s, 6H, Si(CH_3_)_2_). ^13^C NMR (100 MHz, CDCl_3_) δ 164.5 (C=O), 155.7 (Ph C-3), 141.7 (Ph C-1), 140.2 (Ph C-1′), 138.2 (C-4), 129.4 (Ph C-5), 129.0 (C-3), 128.4 (Ph C-3′,5′), 127.8 (Ph C-4′), 127.0 (Ph C-2′,6′), 119.9 (Ph C-6), 119.5 (Ph C-4), 118.7 (Ph C-2), 76.4 (CHPh_2_), 53.2 (C-2), 50.7 (C-6), 45.7 (NCH_3_), 26.6 (C-5), 25.5 (C(CH_3_)_3_), 18.2 (C(CH_3_)_3_), −4.47 (SiCH_3_), −4.49 (SiCH_3_). IR (film) *ν*_max_ 2930, 1714, 1602, 1486, 1253, 1141, 1084, 1026, 838, 784, 699. HRMS (ESI) (m/z) calcd for C_26_H_36_NO_3_Si [M + H]^+^: 438.2459; found 438.2464.

(4-((*tert*-butyldimethylsilyl)oxy)phenyl)(phenyl)methyl 1-methyl-1,2,5,6-tetrahydropyridine-3-carboxylate (**15c**). Following the general procedure on a 0.25 mmol scale, arecaidine was esterified using **13c**. Purification by flash column chromatography (0–6% MeOH in CH_2_Cl_2_) afforded the title compound **15c** (94 mg, 86%) as a yellow oil. ^1^H NMR (400 MHz, CDCl_3_) δ 7.33 (m, 4H, Ph H-2′,3′,5′,6′), 7.27 (m, 1H, Ph H-4′), 7.21 (m, 2H, Ph H-2,6), 7.14 (m, 1H, H-4), 6.93 (s, 1H, CHPh_2_), 6.80 (m, 2H, Ph H-3,5), 3.22 (m, 2H, H-2), 2.51 (m, 2H, H-6), 2.42 (s, 3H, NCH_3_), 2.39 (m, 2H, H-5), 0.98 (s, 9H, C(CH_3_)_3_), 0.20 (s, 6H, Si(CH_3_)_2_). ^13^C NMR (100 MHz, CDCl_3_) δ 164.6 (C=O), 155.3 (Ph C-4), 140.5 (Ph C-1′), 138.0 (C-4), 132.9 (Ph C-1), 129.0 (C-3), 128.4 (Ph C-2,6), 128.3 (Ph C-3′,5′), 127.6 (Ph C-4′), 126.8 (Ph C-2′,6′), 119.8 (Ph C-3,5), 76.3 (CHPh_2_), 53.1 (C-2), 50.7 (C-6), 45.6 (NCH_3_), 26.6 (C-5), 25.6 (C(CH_3_)_3_), 18.0 (C(CH_3_)_3_), −4.5 (Si(CH_3_)_2_). IR (film) *ν*_max_ 1711, 1509, 1252, 1189, 1140, 1083, 1025, 913, 839, 781, 699. HRMS (ESI) (m/z) calcd for C_26_H_36_NO_3_Si [M + H]^+^: 438.2459; found 438.2461.

2-((*tert*-butyldimethylsilyl)oxy)benzyl 1-methyl-1,2,5,6-tetrahydropyridine-3-carboxylate (**16a**). Following the general procedure on a 0.25 mmol scale, arecaidine was esterified using **14a**. Purification by flash column chromatography (0–6% MeOH in CH_2_Cl_2_) afforded the title compound **16a** (72 mg, 80%) as a yellow oil. ^1^H NMR (400 MHz, CDCl_3_) δ 7.31 (m, 1H, Ph H-6), 7.19 (m, 1H, Ph H-4), 7.02 (m, 1H, H-4), 6.93 (m, 1H, Ph H-5), 6.81 (m, 1H, Ph H-3), 5.20 (s, 2H, OCH_2_), 3.16 (m, 2H, H-2), 2.48 (m, 2H, H-6), 2.39 (s, 3H, NCH_3_), 2.35 (m, 2H, H-5), 1.00 (s, 9H, C(CH_3_)_3_), 0.24 (s, 6H, Si(CH_3_)_2_). ^13^C NMR (100 MHz, CDCl_3_) δ 165.6 (C=O), 153.8 (Ph C-2), 137.5 (C-4), 129.9 (Ph C-6), 129.2 (Ph C-4), 129.0 (C-3), 126.7 (Ph C-1), 121.0 (Ph C-5), 118.4 (Ph C-3), 61.9 (OCH_2_), 53.2 (C-2), 50.7 (C-6), 45.6 (NCH_3_), 26.5 (C-5), 25.6 (C(CH_3_)_3_), −4.3 (Si(CH_3_)_2_). IR (film) *ν*_max_ 2931, 1712, 1492, 1456, 1256, 1141, 1086, 1027, 921, 838, 782. HRMS (ESI) (m/z) calcd for C_20_H_32_NO_3_Si [M + H]^+^: 362.2146; found 362.2165.

3-((*tert*-butyldimethylsilyl)oxy)benzyl 1-methyl-1,2,5,6-tetrahydropyridine-3-carboxylate (**16b**). Following the general procedure on a 0.25 mmol scale, arecaidine was esterified using **14b**. Purification by flash column chromatography (0–6% MeOH in CH_2_Cl_2_) afforded the title compound **16b** (73 mg, 81%) as a yellow oil. ^1^H NMR (400 MHz, CDCl_3_) δ 7.20 (m, 1H, Ph H-5), 7.05 (m, 1H, H-4), 6.93 (m, 1H, Ph H-6), 6.83 (m, 1H, Ph H-2), 6.78 (m, 1H, Ph H-4), 5.12 (s, 2H, OCH_2_), 3.17 (m, 2H, H-2), 2.49 (m, 2H, H-6), 2.40 (s, 3H, NCH_3_), 2.36 (m, 2H, H-5), 0.98 (s, 9H, C(CH_3_)_3_), 0.19 (s, 6H, Si(CH_3_)_2_). ^13^C NMR (100 MHz, CDCl_3_) δ 165.4 (C=O), 155.7 (Ph C-3), 137.9 (C-4), 137.5 (Ph C-1), 129.4 (Ph C-5), 128.9 (C-3), 120.7 (Ph C-6), 119.7 (Ph C-4), 119.5 (Ph C-2), 65.8 (OCH_2_), 53.2 (C-2), 50.7 (C-6), 45.7 (NCH_3_), 26.6 (C-5), 25.6 (C(CH_3_)_3_), −4.5 (Si(CH_3_)_2_). IR (film) *ν*_max_ 2930, 1712, 1588, 1487, 1444, 1257, 1141, 1089, 1030, 959, 839, 782, 693. HRMS (ESI) (m/z) calcd for C_20_H_32_NO_3_Si [M + H]^+^: 362.2146; found 362.2166.

4-((*tert*-butyldimethylsilyl)oxy)benzyl 1-methyl-1,2,5,6-tetrahydropyridine-3-carboxylate (**16c**). Following the general procedure on a 0.25 mmol scale, arecaidine was esterified using **14c**. Purification by flash column chromatography (0–6% MeOH in CH_2_Cl_2_) afforded the title compound **16c** (80 mg, 89%) as a yellow oil. ^1^H NMR (400 MHz, CDCl_3_) δ 7.22 (m, 2H, Ph H-2,6), 7.02 (m, 1H, H-4), 6.80 (m, 2H, Ph H-3,5), 5.10 (s, 2H, OCH_2_), 3.15 (m, 2H, H-2), 2.47 (m, 2H, H-6), 2.39 (s, 3H, NCH_3_), 2.35 (m, 2H, H-5), 0.97 (s, 9H, C(CH_3_)_3_), 0.19 (s, 6H, Si(CH_3_)_2_). ^13^C NMR (100 MHz, CDCl_3_) δ 165.6 (C=O), 155.6 (Ph C-4), 137.7 (C-4), 129.7 (Ph C-2,6), 129.0 (C-3), 128.8 (Ph C-1), 120.0 (Ph C-3,5), 65.8 (OCH_2_), 53.1 (C-2), 50.7 (C-6), 45.7 (NCH_3_), 26.6 (C-5), 25.6 (C(CH_3_)_3_), −4.5 (Si(CH_3_)_2_). IR (film) *ν*_max_ 2931, 1712, 1512, 1254, 1141, 1086, 1028, 914, 839, 781. HRMS (ESI) (m/z) calcd for C_20_H_32_NO_3_Si [M + H]^+^: 362.2146; found 362.2166.

2-((tert-butyldimethylsilyl)oxy)-1-phenylethyl 1-methyl-1,2,5,6-tetrahydropyridine-3-carboxylate (**21**). Following the general procedure on a 0.25 mmol scale, arecaidine was esterified using **20**. Purification by flash column chromatography (0–6% MeOH in CH_2_Cl_2_) afforded the title compound **21** (71 mg, 76%) as a yellow oil. ^1^H NMR (400 MHz, CDCl_3_) δ 7.33 (m, 2H, Ph H-2,6), 7.32 (m, 2H, Ph H-3,5), 7.28 (m, 1H, Ph H-4), 7.08 (m, 1H, H-4), 5.89 (dd, *J* = 7.3, 4.6 Hz, 1H, CHCH_2_OH), 3.89 (dd, *J* = 10.9, 7.3 Hz, 1H, CHCH_2_OH), 3.80 (dd, *J* = 10.9, 4.6 Hz, 1H, CH_2_), 3.18 (m, 2H, H-2), 2.48 (m, 2H, H-6), 2.40 (s, 3H, NCH_3_), 2.37 (m, 2H, H-5), 0.84 (s, 9H, C(CH_3_)_3_), −0.02 (s, 6H, Si(CH_3_)_2_). ^13^C NMR (100 MHz, CDCl_3_) δ 164.8 (C=O), 137.9 (Ph C-1), 137.6 (C-4), 129.1 (C-3), 128.2 (Ph C-3,5), 127.9 (Ph C-4), 126.6 (Ph C-2,6), 76.2 (CHCH_2_OH), 66.1 (CHCH_2_OH), 5h3.2 (C-2), 50.7 (C-6), 45.6 (NCH_3_), 26.5 (C-5), 25.7 (C(CH_3_)_3_), 18.1 (C(CH_3_)_3_), –5.55 (SiCH_3_), −5.59 (SiCH_3_). IR (film) *ν*_max_ 2928, 1713, 1253, 1128, 1088, 1027, 835, 777, 699. HRMS (ESI) (m/z) calcd for C_21_H_34_NO_3_Si [M + H]^+^: 376.2302; found 376.2302.

2-hydroxy-2,2-diphenylethyl 1-methyl-1,2,5,6-tetrahydropyridine-3-carboxylate (**25**). Following the general procedure on a 0.25 mmol scale, arecaidine was esterified using **24**. Purification by flash column chromatography (0–10% MeOH in CH_2_Cl_2_) afforded the title compound **25** (58 mg, 69%) as a colorless solid. mp 150–153 °C. ^1^H NMR (400 MHz, CDCl_3_) δ 7.41 (m, 4H, Ph H-2,6), 7.32 (m, 4H, Ph H-3,5), 7.25 (m, 2H, Ph H-4), 6.85 (m, 1H, H-4), 4.71 (s, 2H, (CO)OCH_2_), 3.46 (br s, 1H, OH), 3.02 (m, 2H, H-2), 2.45 (m, 2H, H-6), 2.33 (s, 3H, NCH_3_), 2.29 (m, 2H, H-5). ^13^C NMR (100 MHz, CDCl_3_) δ 165.4 (C=O), 143.7 (Ph C-1), 138.4 (C-4), 128.3 (C-3), 128.2 (Ph C-3,5), 127.4 (Ph C-4), 126.4 (Ph C-2,6), 77.3 (CPh_2_), 70.0 ((CO)OCH_2_), 52.8 (C-2), 50.5 (C-6), 45.4 (NCH_3_), 26.4 (C-5). IR (film) *ν*_max_ 2797, 1707, 1449, 1262, 1140, 1094, 1028, 700. HRMS (ESI) (m/z) calcd for C_21_H_24_NO_3_ [M + H]^+^: 338.1751; found 338.1764.

2′-hydroxy-[1,1′-biphenyl]-2-yl 1-methyl-1,2,5,6-tetrahydropyridine-3-carboxylate (**27**). Following the general procedure on a 0.25 mmol scale, arecaidine was esterified using **26**. Purification by flash column chromatography (0–10% MeOH in CH_2_Cl_2_) afforded the title compound **27** (60 mg, 78%) as a pale orange solid. mp 142–144 °C. ^1^H NMR (400 MHz, CDCl_3_) δ 7.43 (m, 1H, Ph H-4), 7.39 (m, 1H, Ph H-6), 7.33 (m, 1H, Ph H-5), 7.24 (m, 1H, Ph H-3), 7.21 (m, 1H, Ph H-4′), 7.14 (dd, *J* = 7.6, 1.7 Hz, 1H, Ph H-6′), 7.02 (m, 1H, H-4), 6.92 (m, 1H, Ph H-5′), 6.80 (dd, *J* = 8.2, 1.0 Hz, 1H, Ph H-3′), 3.05 (m, 2H, H-2), 2.47 (t, *J* = 5.6 Hz, 2H, H-6) 2.35 (m, 2H, H-5), 2.33 (s, 3H, NCH_3_). ^13^C NMR (100 MHz, CDCl_3_) δ 164.0 (C=O), 153.6 (Ph C-2′), 148.5 (Ph C-2), 139.7 (C-4), 131.7 (Ph C-6), 130.9 (Ph C-6′), 130.7 (Ph C-1), 129.4 (Ph C-4′), 129.0 (Ph C-4), 127.9 (C-3), 126.3 (Ph C-5), 124.3 (Ph C-1′), 123.0 (Ph C-3), 120.1 (Ph C-5′), 115.9 (Ph C-3′), 52.7 (C-2), 50.5 (C-6), 45.4 (NCH_3_), 26.4 (C-5). IR (film) *ν*_max_ 1730, 1440, 1235, 1191, 1122, 1045, 1013, 755. HRMS (ESI) (m/z) calcd for C_19_H_20_NO_3_ [M + H]^+^: 310.1438; found 310.1436.

#### 3.1.4. General Procedure for the TBAF-Mediated TBS Deprotection of Arecaidine Esters (**10a**−**c**, **17b**, **18b**, **22**)

To an ice-cooled solution of TBS-protected alcohol in anhydrous THF (0.2 M) was added TBAF (1.0 M in THF, 1.0 equiv) and it was stirred for 20 min at the indicated temperature. Then, volatiles were removed under reduced pressure and the crude residue was purified by flash column chromatography to give the desired deprotected alcohol.

(2-(hydroxymethyl)phenyl)(phenyl)methyl 1-methyl-1,2,5,6-tetrahydropyridine-3-carboxylate (**10a**). Following the general procedure on a 0.10 mmol scale, **9a** was deprotected. Purification by flash column chromatography (0–10% MeOH in CH_2_Cl_2_) afforded the title compound **10a** (29 mg, 87%) as a yellow oil. ^1^H NMR (400 MHz, CDCl_3_) δ 7.41 (m, 1H, Ph H-3), 7.39 (m, 1H, Ph H-6), 7.30 (m, 4H, Ph H-2′,3′,5′,6′), 7.29 (m, 2H, Ph H-4,5), 7.27 (m, 1H, Ph H-4′), 7.20 (s, 1H, CHPh_2_), 7.13 (m, 1H, H-4), 4.84 (d, *J* = 12.7 Hz, 1H, CH_2_OH), 4.63 (d, *J* = 12.7 Hz, 1H, CH_2_OH), 3.32 (br s, 1H, OH), 3.18 (m, 2H, H-2), 2.50 (m, 2H, H-6), 2.39 (s, 3H, NCH_3_), 2.38 (m, 2H, H-5). ^13^C NMR (100 MHz, CDCl_3_) δ 164.8 (C=O), 139.6 (Ph C-1′), 138.6 (Ph C-2; C-4), 137.9 (Ph C-1), 128.9 (Ph C-3), 128.6 (C-3), 128.43 (Ph C-3′,5′), 128.37 (Ph C-4), 128.2 (Ph C-5), 127.8 (Ph C-4′), 127.7 (Ph C-6), 126.9 (Ph C-2′,6′), 73.5 (CHPh_2_), 62.7 (CH_2_OH), 52.9 (C-2), 50.6 (C-6), 45.5 (NCH_3_), 26.5 (C-5). IR (film) *ν*_max_ 1708, 1452, 1245, 1190, 1138, 1085, 1022, 758, 699. HRMS (ESI) (m/z) calcd for C_21_H_24_NO_3_ [M + H]^+^: 338.1751; found 338.1756.

(3-(hydroxymethyl)phenyl)(phenyl)methyl 1-methyl-1,2,5,6-tetrahydropyridine-3-carboxylate (**10b**). Following the general procedure on a 0.10 mmol scale, **9b** was deprotected. Purification by flash column chromatography (0–10% MeOH in CH_2_Cl_2_) afforded the title compound **10b** (29 mg, 86%) as a pale-yellow solid. mp 125–128 °C. ^1^H NMR (400 MHz, CDCl_3_) δ 7.33 (m, 2H, Ph H-2′,6′), 7.32 (m, 3H, Ph H-2; Ph H-3′,5′), 7.28 (m, 1H, Ph H-5), 7.27 (m, 1H, Ph H-4′), 7.25 (m, 2H, Ph H-4,6), 7.12 (m, 1H, H-4), 6.92 (s, 1H, CHPh_2_), 4.60 (s, 2H, CH_2_OH), 3.37 (br s, 1H, OH), 3.20 (m, 2H, H-2), 2.49 (m, 2H, H-6), 2.37 (s, 3H, NCH_3_), 2.37 (m, 2H, H-5). ^13^C NMR (100 MHz, CDCl_3_) δ 164.4 (C=O), 141.6 (Ph C-3), 140.5 (Ph C-1), 140.1 (Ph C-1′), 138.2 (C-4), 129.0 (C-3), 128.6 (Ph C-5), 128.5 (Ph C-3′,5′; C-3), 127.8 (Ph C-4′), 127.0 (Ph C-2′,6′), 126.4 (Ph C-4), 126.0 (Ph C-6), 125.4 (Ph C-2), 69.2 (CHPh_2_), 64.7 (CH_2_OH), 52.8 (C-2), 50.5 (C-6), 45.4 (NCH_3_), 26.3 (C-5). IR (film) *ν*_max_ 1707, 1452, 1396, 1243, 1190, 1137, 1085, 1022, 787, 733, 698, 607. HRMS (ESI) (m/z) calcd for C_21_H_24_NO_3_ [M + H]^+^: 338.1751; found 338.1766.

(4-(hydroxymethyl)phenyl)(phenyl)methyl 1-methyl-1,2,5,6-tetrahydropyridine-3-carboxylate (**10c**). Following the general procedure on a 0.10 mmol scale, **9c** was deprotected. Purification by flash column chromatography (0–10% MeOH in CH_2_Cl_2_) afforded the title compound **10c** (31 mg, 92%) as a yellow oil. ^1^H NMR (400 MHz, CDCl_3_) δ 7.32 (m, 4H, Ph H-2′,3′,5′,6′), 7.30 (m, 4H, Ph H-2,3,5,6), 7.27 (m, 1H, Ph H-4′), 7.13 (m, 1H, H-4), 6.91 (s, 1H, CHPh_2_), 4.61 (s, 2H, CH_2_OH), 3.38 (br s, 1H, OH), 3.22 (m, 2H, H-2), 2.53 (m, 2H, H-6), 2.40 (s, 3H, NCH_3_), 2.39 (m, 2H, H-5). ^13^C NMR (100 MHz, CDCl_3_) δ 164.4 (C=O), 140.9 (Ph C-4), 140.1 (Ph C-1′), 139.4 (Ph C-1), 138.2 (C-4), 128.5 (Ph C-3′,5′), 128.4 (C-3), 127.9 (Ph C-4′), 127.2 (Ph C-2,6), 127.0 (Ph C-3,5), 126.9 (Ph C-2′,6′), 76.7 (CHPh_2_), 64.6 (CH_2_OH), 52.8 (C-2), 50.5 (C-6), 45.4 (NCH_3_), 26.2 (C-5). IR (film) *ν*_max_ 1709, 1455, 1396, 1248, 1139, 1085, 1024, 699. HRMS (ESI) (m/z) calcd for C_21_H_24_NO_3_ [M + H]^+^: 338.1751; found 338.1765.

(3-hydroxyphenyl)(phenyl)methyl 1-methyl-1,2,5,6-tetrahydropyridine-3-carboxylate (**17b**). Following the general procedure on a 0.10 mmol scale, **15b** was deprotected. Purification by flash column chromatography (0–10% MeOH in CH_2_Cl_2_) afforded the title compound **17b** (29 mg, 90%) as an off-white solid. mp 143–144 °C. ^1^H NMR (400 MHz, CDCl_3_) δ 7.31 (m, 4H, Ph H-2′,3′,5′,6′), 7.26 (m, 1H, Ph H-4′), 7.11 (m, 1H, Ph H-5), 7.13 (m, 1H, H-4), 6.85 (s, 1H, CHPh_2_), 6.82 (m, 1H, Ph H-6), 6.74 (m, 1H, Ph H-2), 6.65 (m, 1H, Ph H-4), 3.28 (m, 2H, H-2), 2.56 (m, 2H, H-6), 2.41 (s, 3H, NCH_3_), 2.39 (m, 2H, H-5). ^13^C NMR (100 MHz, CDCl_3_) δ 164.4 (C=O), 156.3 (Ph C-3), 141.7 (Ph C-1), 140.0 (Ph C-1′), 138.3 (C-4), 129.7 (Ph C-5), 128.5 (Ph C-3′,5′), 128.2 (C-3), 127.9 (Ph C-4′), 127.0 (Ph C-2′,6′), 118.6 (Ph C-6), 115.1 (Ph C-4), 114.2 (Ph C-2), 76.8 (CHPh_2_), 52.7 (C-2), 50.4 (C-6), 45.2 (NCH_3_), 25.9 (C-5). IR (film) *ν*_max_ 2949, 1710, 1588, 1453, 1248, 1127, 1086, 1022, 735, 701. HRMS (ESI) (m/z) calcd for C_20_H_22_NO_3_ [M + H]^+^: 324.1594; found 324.1588.

3-hydroxybenzyl 1-methyl-1,2,5,6-tetrahydropyridine-3-carboxylate (**18b**). Following the general procedure on a 0.10 mmol scale, **16b** was deprotected. Purification by flash column chromatography (0–10% MeOH in CH_2_Cl_2_) afforded the title compound **18b** (20 mg, 82%) as an off-white solid. mp 115–119 °C. ^1^H NMR (400 MHz, CDCl_3_) δ 8.84 (br s, 1H, OH), 7.15 (m, 1H, Ph H-5), 6.82 (m, 1H, H-6), 6.72 (m, 1H, Ph H-2), 6.71 (m, 1H, Ph H-4), 5.07 (s, 2H, OCH_2_), 3.28 (m, 2H, H-2), 2.59 (m, 2H, H-6), 2.45 (s, 3H, NCH_3_), 2.40 (m, 2H, H-5). ^13^C NMR (100 MHz, CDCl_3_) δ 165.3 (C=O), 157.0 (Ph C-3), 138.2 (C-4), 137.2 (C-1), 129.7 (Ph C-5), 128.0 (C-3), 119.3 (Ph C-6), 115.6 (Ph C-4), 115.2 (Ph C-2), 66.2 (OCH_2_), 52.6 (C-2), 50.4 (C-6), 45.2 (NCH_3_), 25.8 (C-5). IR (film) *ν*_max_ 1707, 1589, 1458, 1398, 1261, 1127, 1089, 1026, 787, 719, 694. HRMS (ESI) (m/z) calcd for C_14_H_18_NO_3_ [M + H]^+^: 248.1281; found 248.1287.

2-hydroxy-1-phenylethyl 1-methyl-1,2,5,6-tetrahydropyridine-3-carboxylate (**22**). Following the general procedure on a 0.10 mmol scale, **21** was deprotected. Purification by flash column chromatography (0–10% MeOH in CH_2_Cl_2_) afforded the title compound **22** (17 mg, 65%) as a yellow oil. ^1^H NMR (400 MHz, CDCl_3_) δ 7.39 (m, 2H, Ph H-2,6), 7.36 (m, 2H, Ph H-3,5), 7.31 (m, 1H, Ph H-4), 7.04 (m, 1H, H-4), 4.98 (dd, *J* = 8.1, 3.5 Hz, 1H, CHCH_2_OH), 4.34 (dd, *J* = 11.5, 3.5 Hz, 1H, CHCH_2_OH), 4.24 (dd, *J* = 11.5, 8.1 Hz, 1H, CH_2_), 3.17 (m, 2H, H-2), 3.01 (br s, 1H, OH), 2.53 (m, 2H, H-6), 2.42 (s, 3H, NCH_3_), 2.40 (m, 2H, H-5). ^13^C NMR (100 MHz, CDCl_3_) δ 165.6 (C=O), 140.1 (Ph C-1), 138.3 (C-4), 128.5 (Ph C-3,5), 128.4 (C-3), 128.2 (Ph C-4), 126.1 (Ph C-2,6), 72.4 (CHCH_2_OH), 69.4 (CHCH_2_OH), 52.9 (C-2), 50.6 (C-6), 45.5 (NCH_3_), 26.4 (C-5). IR (film) *ν*_max_ 1706, 1656, 1453, 1398, 1261, 1192, 1138, 1089, 1026, 790, 755, 701. HRMS (ESI) (m/z) calcd for C_15_H_20_NO_3_ [M + H]^+^: 262.1438; found 363.1432.

### 3.2. High-Throughput HPLC-logD

High-throughput HPLC-logD values were determined as published previously using the HPLC system described above equipped with an apHERA C18 column (10 × 6 mm, 5 µm, Supelco, Bellefonte, PA, USA) [21,40]. Briefly, a mixture of toluene (≥98%, Sigma-Aldrich, St. Louis, MO, USA) and triphenylene (≥99.9%, Carl Roth, Karlsruhe, Germany) was used as internal standard. Samples were dissolved in the internal standard mixture. Using gradient elution, the injection volume was set to 7 µL, the flow rate was 1.5 mL/min, and the mobile phase consisted of a mixture of methanol and 0.01 M sodium phosphate buffer pH 7.4. The HPLC-logD values were derived from the measured retention times following the previously published equation [21,40].

### 3.3. Biological Evaluation

For the biological evaluation of the herein synthesized compounds, we essentially followed our previously described workflow and steps [25].

#### 3.3.1. Materials and Methods

Reagents and cell culture media were purchased from Sigma-Aldrich (St. Louis, MO, USA) and Life Technologies (Waltham, MA, USA) unless specified otherwise. Commercially obtained compounds had >98% purity. [*N*-methyl-^3^H]scopolamine methyl chloride ([^3^H]NMS) (specific activity 85.4 Ci/mmol) was purchased from PerkinElmer (Waltham, MA, USA). All analytical buffers were prepared in double distilled water (GFL water still 2004). The protease inhibitor cocktail powder (P2714-1BTL, Sigma-Aldrich, St. Louis, MO, USA) was dissolved in 10 mL of water and used as such. Stock solutions of all compounds were prepared in pure DMSO.

#### 3.3.2. Cell Culture

Chinese hamster ovary (CHO-K1) cells stably expressing the *h*M_1_-*h*M_5_ receptors were obtained from Missouri University of Science and Technology cDNA Resource Center (Cell Catalog#: CEM1000000, CEM2000000, CEM3000000, CEM4000000, CEM5000000) and cultivated in Gibco™ Ham’s F-12 Nutrient Mixture supplemented with 10% (*v*/*v*) Gibco™ FBS, 250 mg/mL Geneticin^®^ (G418, Thermo Fisher, Waltham, MA, USA), and L-glutamine (1%; 200 nM) at 37 °C in a 5% CO_2_ humidified atmosphere. No other antibiotics were applied. Gibco™ Trypsin-EDTA (0.05%) was used for passaging cells.

#### 3.3.3. Cell Viability (MTT Assay)

CHO-*h*M_1_ cells were harvested from culture flasks by trypsinization and seeded into 96-well microculture plates (Corning^®^, Corning, NY, USA) in densities of 4000 cells/well (100 µL/well). After a 24 h preincubation, cells were exposed in triplicates for each concentration level to dilutions of the test compounds in complete culture medium (100 µL/well) for 72 h. At the end of the exposure period, the compound solutions were replaced with 100 µL of nonsupplemented RPMI 1640 medium and 3-(4,5-dimethylthiazol-2-yl)-2,5-diphenyltetrazoliumbromid (MTT reagent in PBS, 5 mg/mL) mixed in a 6:1 ratio. After incubation for 4 h, the medium was removed, and the formazan product was solved in DMSO (100 µL/well). Optical densities at 490 nm were measured with a microplate reader (Tecan Infinite^®^ 200 PRO, Männedorf, Switzerland) using a reference wavelength of 690 nm to correct for unspecific absorption. The quantity of viable cells was normalized to untreated controls.

#### 3.3.4. Radioligand Binding Experiments

Cell membranes bearing *h*M_1_-*h*M_5_ receptors were prepared as described previously [10]. Briefly, stably transfected CHO-K1 cells were grown to at least 80% confluency in T175 flasks, washed with ice-cold DPBS, and scraped into a mixture of ice-cold 2 mL 10 mM Tris-HCl, 1 mM EDTA-buffer, pH 7.4 and 200 µL protease inhibitor. A cell homogenate was prepared by passing the cell suspension through a G29 needle. The cell homogenates corresponding to two T175 flasks were combined and subsequently centrifuged (10 min, 1000× *g*, 4 °C). Ultracentrifugation of the supernatant (1 h, 100,000× *g*, 4 °C) yielded a membrane pellet, which was suspended in 250 µL 50 mM Tris-HCl buffer, pH 7.4 and stored at −80 °C.

Inhibition constants (*K*_i_) were determined by means of a competitive radioligand binding assay using 50 mM Tris-HCl, 10 mM MgCl_2_, 1 mM EDTA, pH 7.4 as assay buffer as described previously [11]. Then, 5 µL of test compound in DMSO, 50 µL of [^3^H]NMS in assay buffer, and 445 µL of membrane suspension in assay buffer were incubated for 90 min at 23 °C in PP tubes. Maximum binding was measured by using 5 µL DMSO, and nonspecific binding was measured by using 5 µL of 1 µM scopolamine in DMSO. The effective concentration of [^3^H]NMS was 0.2 nM, 0.3 nM, 0.8 nM, 0.2 nM, and 1 nM for M_1_–M_5_ and 4–30 µg membrane were used per tube. The membrane-bound radioactivity was recovered by filtration through Whatman™ GF/B glass fiber filters presoaked in aqueous 0.1% PEI using an M-36 tygon tubed cell harvester (Brandel^®^, Gaithersburg, MD, USA). Membranes were washed three times with ice-cold washing buffer (50 mM Tris, pH 7.4) before being dried, transferred to 2 mL scintillation cocktail (UltimaGold™, high flashpoint LSC cocktail, PerkinElmer, Waltham, MA, USA) and counted in a β-counter (Hidex TDCR liquid scintillation counter in CPM mode). IC_50_ values were calculated by a variable slope logistic regression using at least five distinct concentrations of test compounds, pipetted in triplicates. *K*_i_ values were then calculated with the help of the Cheng–Prusoff equation using the following *K*_D_ values of [^3^H]NMS for *h*M_1_-*h*M_5_: 0.18, 0.24, 0.23, 0.10, and 0.35 nM.

#### 3.3.5. Fluo-4 Calcium Assay for Agonist-Antagonist Discrimination

For the Fluo-4 Direct™ assay kit (Invitrogen, Waltham, MA, USA), 100 µL of a 5 × 10^5^ cells/mL suspension of CHO-*h*M_1_ cells was seeded in black clear-bottom 96-well plates (Corning^®^, Corning, NY, USA). After settling of the cells for 24 h, the kit was used according to the manufacturer’s protocol. In detail, the medium was removed, and 50 µL of Hanks’ balanced salt solution (HBSS) was added, followed by 50 µL of the Fluo-4 buffer solution (including probenecid). The 96-well plates were incubated for 60 min at 37 °C in the dark. For the agonist assay, 100 µL of a double-concentrated dilution series of carbachol (positive control) and compounds was added with the end concentration of 100, 10, 1, 0.1, 0.01 and 0 µM. The relative fluorescence was measured with an excitation wavelength of 494 nm and an emission wavelength of 516 nm. For the antagonist assay, 50 µL of a 4-fold concentrated dilution series of scopolamine hydrochloride (positive control) and compounds was added. Subsequently an 80 µM stock solution of carbachol was added to all wells, and the relative fluorescence was measured with an excitation wavelength of 494 nm and an emission wavelength of 516 nm. Stock solutions of the compounds were in DMSO with a final concentration not exceeding 1% of DMSO.

#### 3.3.6. Data Analysis and Statistics

Data analysis in general was performed using Prism 9.00 (GraphPad Software, San Diego, CA, USA) or Microsoft Excel^®^ 365. Data are presented as means ± standard deviation (SD) for at least 3 independent experiments unless indicated otherwise.

### 3.4. Molecular Docking

All docking experiments were carried out using AutoDock Vina 1.1 with default settings [41]. The crystal structure of the M_1_ muscarinic acetylcholine receptor (PDB 5CXV) and M5 muscarinic acetylcholine receptor (PDB 6OL9) were used for docking [28,42]. The performance of the docking algorithm was validated in a redocking experiment, in which the cocrystallized ligand’s binding pose was reproduced with an acceptable RMSD of 0.252 Å in the binding pocket of M_1_ and 0.294 Å in the binding pocket of M_5_ [43]. Before docking, molecules were set to their energetically most favorable ionization state at pH 7.4 using the FixpKa program from OpenEye [44]. Docking results and the corresponding receptor–ligand interactions were analyzed with LigandScout 4.4.5 [45]. To visualize the spatial arrangement of such interactions, 3D and 2D pharmacophores were generated using the same software tool. Docking poses were additionally visualized using PyMOL [46]. The highest ranked pose of each docked compound exhibiting an ionic interaction with Asp105^3.32^ was selected as a representative.

## 4. Conclusions

In summary, we successfully synthesized a series of hydroxylated arecaidine esters displaying favorable physicochemical properties for potential BBB penetration. The most affine compounds of this series **10b**, **10c**, and **17b** were characterized by binding constants towards *h*M_1_ in the low double-digit and single-digit region—similar to DPMA, the parent compound of this series. While the challenge of prominent subtype selectivity for *h*M_1_ over the other muscarinic subtypes remains unsolved, this study significantly expanded our understanding towards the SAR of arecaidine esters. A noteworthy finding of this study was compound **8b**’s almost threefold preference for the subtype *h*M_5_. The presented docking studies were not able to explain this selectivity switch in comparison to DPMA; however, it serves as a starting point for more sophisticated computational studies and might guide the way towards M_5_-preferring ligands.

## Figures and Tables

**Table 1:** HPLC-logD and CNS MPO score of the synthesized hydroxylated arecaidine esters.

Cmpd.	HPLC-logD	CNS MPO Score ^1^
**8a**	0.79 ± 0.01	5.71
**8b**	0.87 ± 0.01	5.70
**8c**	1.03 ± 0.04	5.70
**10b**	2.6 ± 0.1	5.20
**10c**	2.8 ± 0.2	5.20
**17b**	2.69 ± 0.01	4.83
**18b**	1.57 ± 0.04	5.72
**22**	0.92 ± 0.08	5.75
**25**	2.69 ± 0.01	5.34
**27**	2.69 ± 0.01	5.03
DPMA	3.32 ± 0.04 ^2^	4.16

^1^ Calculated using ChemAxon’s CNS multiparameter optimization (MPO) score predictor [19]. ^2^ Value taken from Ozenil et al. [10].

**Table 2:** Inhibition of [^3^H]NMS binding in CHO-*h*M_1-5_ cell membrane preparations and subtype selectivity profiles.

	Affinity: *K*_i_ ± SD (nM)					*x*-Fold Selectivity for *h*M_1_ vs. *h*M_x_^1^			
Cmpd.	*h*M_1_	*h*M_2_	*h*M_3_	*h*M_4_	*h*M_5_	*h*M_2_	*h*M_3_	*h*M_4_	*h*M_5_
**8c**	525 ± 36	>1000 ^2^	663 ± 56	697 ± 132	417 ± 73	>1.9	1.3	1.3	0.8
**10b**	12.5 ± 0.8	198 ± 35	70.4 ± 1.8	32.3 ± 3.8	49.1 ± 8.5	15.8	5.6	2.6	3.9
**10c**	74.0 ± 3.2	980 ± 32	150 ± 26	93 ± 23	72 ± 13	13.2	2.0	1.3	1.0
**17b**	5.5 ± 0.5	172 ± 20	20.4 ± 1.2	18.4 ± 5.8	11.0 ± 1.5	31.3	3.7	3.3	2.0
**18b**	259 ± 75	>1000 ^3^	188 ± 18	634 ± 90	90 ± 27	>3.7	0.7	2.4	0.35
**25**	231 ± 60	>1000 ^3^	283 ± 20	217 ± 23	284 ± 62	>4.3	1.2	0.9	1.2
DPMA	3.1 ± 0.5 ^4^	110 ± 39 ^4^	56 ± 22 ^4^	16 ± 4 ^4^	14 ± 1 ^4^	35.5	18.1	5.2	4.5

^1^ The selectivity is calculated as the ratio of the *K*_i_ values, i.e., *h*M_x_/*h*M_1_. ^2^ Value derived from two independent experiments carried out in triplicate. ^3^ Value derived from two independent single-concentration experiments carried out in triplicate. ^4^ Value taken from Ozenil et al. [10].

## Data Availability

Data are contained within the article and Supplementary Material.

## Supplementary Materials

The following are available online at https://www.mdpi.com/article/10.3390/molecules27103173/s1, Figure S1: Concentration-dependent cell viability of the hydroxylated arecaidine esters assessed in CHO-*h*M_1_ cells using an MTT assay, Figure S2: Dose-dependent Ca^2+^ flux induced by a set of hydroxylated arecaidine esters in CHO-*h*M_1_ cells, Figure S3: Two-dimensional pharmacophores for hydroxylated arecaidine esters **10a**, **17a**, **17c**, **18a**, and **18c**, Figure S4: Two-dimensional pharmacophores for selected hydroxylated arecaidine esters in the orthosteric binding site of M_1_ (PDB 5CXV) with interacting amino acid residues and key interactions highlighted, Figure S5: Superimposed docking poses for the enantiomeric pairs of **10b**, **10c**, and **17b**, Figures S6–S15: Isocratic HPLC chromatograms of the hydroxylated arecaidine esters. Figures S16–S46: ^1^H and ^13^C spectra of the hydroxylated arecaidine esters, Scheme S1: Attempted syntheses of **40**–**43**, Table S1: Percent displacements of [^3^H]NMS binding on cell membranes derived from CHO-K1 cells expressing *h*M_x_ receptors at ligand concentrations corresponding to a *K*_i_ value of 1 µM according to the Cheng–Prusoff equation. Scheme S1. Reagents and conditions: (a) TESCl, imidazole, DMF, 0 °C, (b) PhMgBr, THF, 0 °C → rt, (c) arecaidine, EDC-HCl, 4-DMAP, CH_2_Cl_2_, rt, (d) arecaidine, SOCl_2_, Et_3_N, CH_2_Cl_2_, reflux, (e) PTSA, MeOH/DCM, 0 °C (f) AcCl, MeOH, 0 °C (g) MeSO_3_H, THF, 0 °C, (h) BBr3, CH_2_Cl_2_, −78 °C, (i), BCl_3_, CH_2_Cl_2_, −20 °C (j) Oxone, THF/H_2_O, rt [47,48,49].
