# Supplementary material for: Synthesis, Biological Evaluation, and Docking Studies of Antagonistic Hydroxylated Arecaidine Esters Targeting mAChRs
Source: Molecules. 2022 May 16;27(10):3173. doi: 10.3390/molecules27103173 (PMC9145622; doi:10.3390/molecules27103173)
Supplement: Supplementary file 1 [file molecules-27-03173-s001.zip › molecules-1703378-supplementary.pdf]

molecules

Supporting Information

Synthesis, Biological Evaluation, and Docking Studies of Antagonistic Hydroxylated Arecaidine Esters Targeting mAChRs

Jonas Kilian [1,2], Marlon Millard [2,3], Marius Ozenil [1], Dominik Krause [3], Khadija Ghaderi [3], Wolfgang Holzer [3], Ernst Urban [3], Helmut Spreitzer [3], Wolfgang Wadsak [1,4], Marcus Hacker [1], Thierry Langer [3] and Verena Pichler [3,*]

[1] Department of Biomedical Imaging and Image-guided Therapy, Division of Nuclear Medicine, Medical University of Vienna, 1090 Vienna, Austria; jonas.kilian@meduniwien.ac.at (J.K.); marius.ozenil@meduniwien.ac.at (M.O.); wolfgang.wadsak@meduniwien.ac.at (W.W.); marcus.hacker@meduniwien.ac.at (M.H.)
[2] Vienna Doctoral School of Pharmaceutical, Nutritional and Sport Sciences, University of Vienna, 1090 Vienna, Austria; marlon.millard@univie.ac.at (M.M.)
[3] Department of Pharmaceutical Sciences, Division of Pharmaceutical Chemistry, Faculty of Life Sciences, University of Vienna, 1090 Vienna, Austria; a01449286@unet.univie.ac.at (D.K.); a01448605@unet.univie.ac.at (K.G.); wolfgang.holzer@univie.ac.at (W.H.); ernst.urban@univie.ac.at (E.U.) thierry.langer@univie.ac.at (T.L.)
[4] CBmed GmbH—Center for Biomarker Research in Medicine, 8036 Graz, Austria
* Correspondence: verena.pichler@univie.ac.at (V.P.); Tel.: +43-1-4277-55624

Table of Contents

MTT Assay **3**

Calcium mobilization in CHO-*h*M_1 cells **3**

Attempted Syntheses of 40–43 **4**

2D Pharmacophores and Docking Pose Overlays **6**

Single-concentration Radioligand Binding Assay **8**

Purity measured by HPLC **9**

NMR Spectra **14**

References Error! Bookmark not defined.

MTT Assay

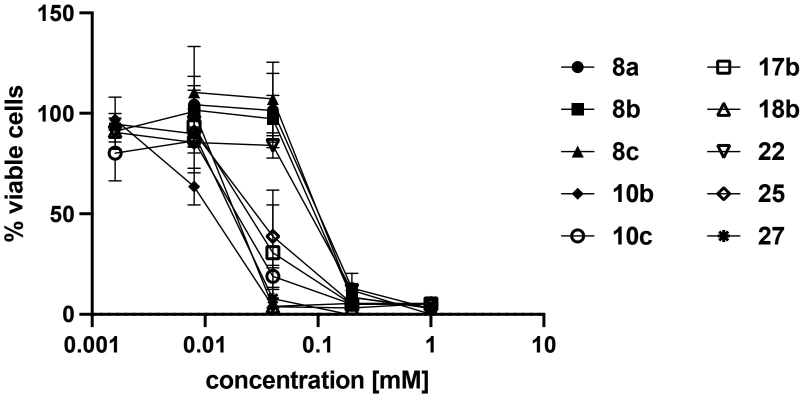

Figure S1. Concentration-dependent cell viability of the hydroxylated arecaidine esters assessed in CHO-*h*M1 cells using an MTT assay. Error bars represent the standard deviation.

Calcium mobilization in CHO-*h*M1 cells

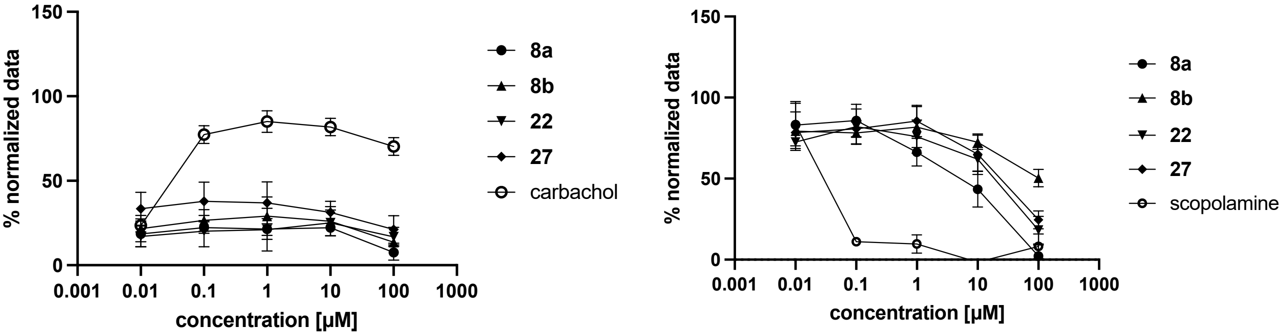

(**a**) (**c**)

Figure S2. Dose-dependent Ca^{2+} flux induced by a set of hydroxylated arecaidine esters in CHO-*h*M1 cells. (a) Agonist dose-response experiment; (b) Antagonist dose-response experiment with the reference agonist carbachol added at a final concentration of 20 µM.

Attempted Syntheses of 40–43

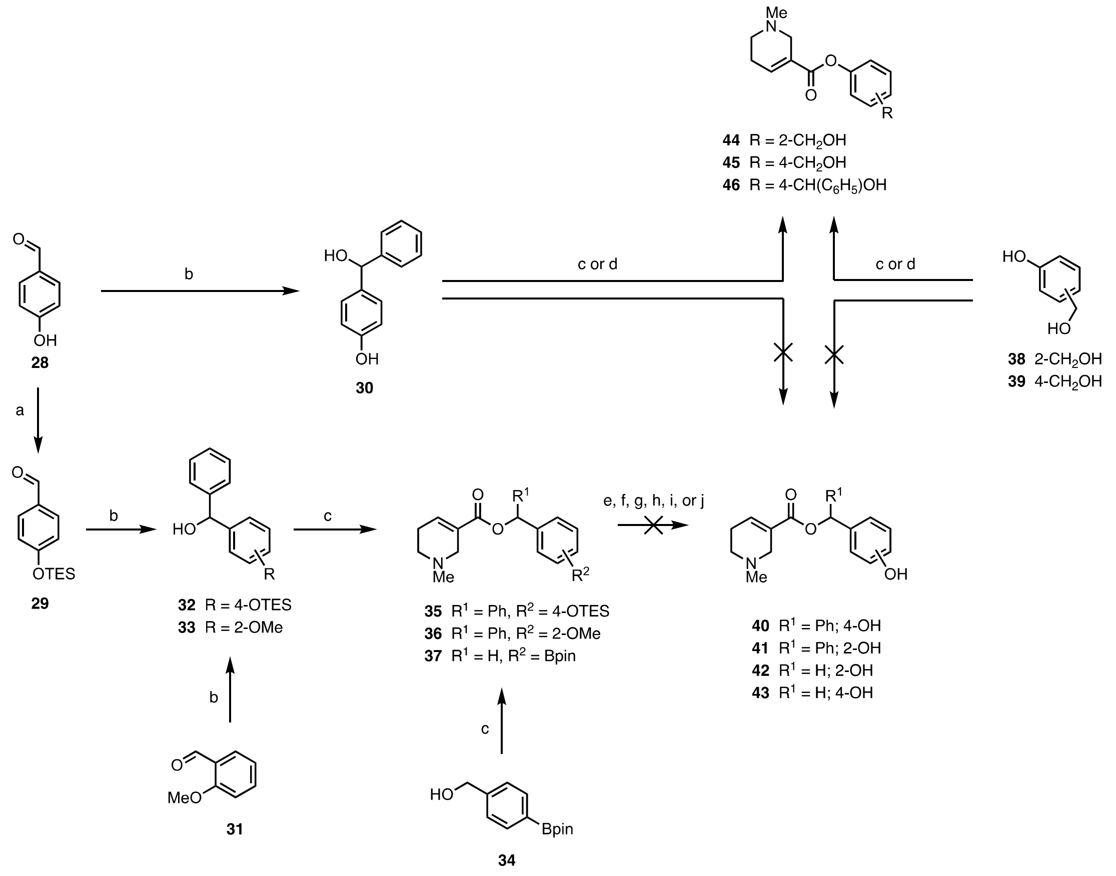

Scheme S1. Reagents and conditions: (a) TESCl, imidazole, DMF, 0 °C, (b) PhMgBr, THF, 0 °C → rt, (c) arecaidine, EDC-HCl, 4-DMAP, CH_2Cl_2, rt, (d) arecaidine, $SOCl_2$, Et_3N, CH_2Cl_2, reflux, (e) PTSA, MeOH/DCM, 0 °C (f) AcCl, MeOH, 0 °C (g) $MeSO_3H$, THF, 0 °C, (h) BBr3, CH_2Cl_2, −78 °C, (i), BCl_3, CH_2Cl_2, −20 °C (j) Oxone, THF/H_2O, rt.

Intermediates **29** [47], **30** [48], and **33** [49] have been described previously and were synthesized following the steps and conditions as outlined in Scheme S1.

Phenyl(4-((triethylsilyl)oxy)phenyl)methanol (**32**). To an ice-cooled solution of aldehyde **29** (236 mg, 1.00 mmol, 1.0 equiv) in anhydrous THF (5 mL) was added dropwise a solution of PhMgBr (1.0 M in THF, 1.1 mL, 1.1 equiv) over a period of 10 min. It was allowed to warm slowly to ambient temperature and was stirred overnight. Then, the mixture was quenched by the addition of water and was extracted with EtOAc (3×). The combined organic layers were dried (Na_2SO_4) and concentrated under reduced pressure. Purification by flash column chromatography (0–20% EtOAc in *n*-heptane) afforded the title compound **32** (280 mg, 89%) as a colorless oil. ^{1}H NMR (500 MHz, $CDCl_3$) δ 7.38 (m, 2H, Ph H-2',6'), 7.34 (m, 2H, Ph H-3',5'), 7.27 (m, 1H, Ph H-4'), 7.21 (m, 2H, Ph H-2,6), 6.81 (m, 2H, Ph H-3,5), 5.79 (s, 1H, $CHPh_2$), 2.19 (br s, 1H, OH), 0.99 (m, 9H, CH_3), 0.73 (m, 6H, CH_2Si). ^{13}C NMR (125 MHz, $CDCl_3$) δ 155.1 (Ph C-4), 143.9 (Ph C-1), 136.6 (Ph C-1), 128.4 (Ph C-3',5'), 127.9 (Ph C-2,6), 127.4 (Ph C-4'), 126.4 (Ph C-2',6'), 119.8 (Ph C-3,5), 75.9 ($CHPh_2$), 6.6 (CH_3), 4.9 (CH_2Si). IR (film) ν_{max} 2956, 2877, 1607, 1508, 1260, 1168, 1008, 908, 730, 699. HRMS (ESI) (m/z) calcd. for $C_{19}H_{26}NaO_2Si$ $[M + Na]^+$: 337.1594; found 337.1595.

The synthesis of arecaidine esters **35**, **36**, **37**, **44**, **45**, and **46** followed the general procedure outlined in the experimental part of this study's manuscript.

Phenyl(4-((triethylsilyl)oxy)phenyl)methyl 1-methyl-1,2,5,6-tetrahydropyridine-3-carboxylate (**35**). Following the general procedure on a 0.25 mmol scale, arecaidine was esterificated using alcohol **32**. Purification by flash column chromatography (0–6% MeOH in CH_2Cl_2) afforded the title compound **35** (56 mg, 73%) as a yellow oil. ^{1}H NMR (500 MHz, $CDCl_3$) δ 7.33 (m, 4H, Ph H-2',3',5',6'), 7.28 (m, 1H, Ph H-4'), 7.18 (m, 2H, Ph H-2,6), 7.13 (m, 1H, H-4), 6.91 (s, 1H,

$CHPh_2$) 6.79 (m, 2H, Ph H-3,5), 3.20 (m, 2H, H-2), 2.51 (m, 2H, H-6), 2.42 (s, 3H, NCH_3), 2.40 (m, 2H, H-5), 0.98 (m, 9H, $CH_2C\underline{H}_3$), 0.73 (m, 6H, $C\underline{H}_2CH_3$). ^{13}C NMR (125 MHz, $CDCl_3$) δ 164.7 (C=O), 155.3 (Ph C-4), 140.6 (Ph C-1'), 138.1 (C-4), 132.9 (Ph C-1), 129.0 (C-3), 128.5 (Ph C-2,6), 128.4 (Ph C-3',5'), 127.7 (Ph C-4'), 126.9 (Ph-C-2',6'), 119.7 (Ph C-3,5), 76.4 ($CHPh_2$), 53.1 (C-2), 50.8 (C-6), 45.7 (NCH_3), 26.6 (C-5), 6.6 ($CH_2\underline{C}H_3$), 4.9 ($\underline{C}H_2CH_3$). IR (film) ν_{max} 2955, 1710, 1509, 1288, 1253, 1239, 1189, 1170, 1140, 1083, 1023, 1000, 971, 906, 746, 722, 698. HRMS (ESI) (m/z) calcd. for $C_{26}H_{38}NO_3Si$ $[M + H]^+$: 438.2459; found 438.2462.

(2-methoxyphenyl)(phenyl)methyl 1-methyl-1,2,5,6-tetrahydropyridine-3-carboxylate (**36**). Following the general procedure on a 0.25 mmol scale, arecaidine was esterificated using alcohol **33**. Purification by flash column chromatography (0–6% MeOH in CH_2Cl_2) afforded the title compound **35** (66 mg, 79%) as a yellow oil. ^1H NMR (400 MHz, $CDCl_3$) δ 7.36 (m, 3H, Ph H-6, Ph H-2',6'), 7.30 (m, 2H, Ph H-3',5'), 7.29 (s, 1H, $CHPh_2$), 7.26 (m, 1H, Ph H-4), 7.24 (m, 1H, Ph H'-4) 7.15 (m, 1H, H-4), 6.95 (m, 1H, Ph H-5), 6.87 (m, 1H, Ph H-3), 3.81 (s, 3H, OCH_3), 3.29 (m, 2H, H-2), 2.59 (m, 2H, H-6), 2.47 (s, 3H, NCH_3), 2.45 (m, 2H, H-5). ^{13}C NMR (100 MHz, $CDCl_3$) δ 164.3 (C=O), 156.4 (Ph C-2), 140.2 (Ph C-1'), 137.7 (C-4), 129.0 (Ph C-4), 128.9 (Ph C-1), 128.5 (Ph C-3), 128.2 (Ph C-3',5'), 127.6 (Ph C-4'), 127.1 (Ph C-2',6'), 126.9 (Ph C-6), 120.6 (Ph C-5), 110.8 (Ph C-3), 71.6 ($CHPh_2$), 55.5 (OCH_3), 52.9 (C-2), 50.6 (C-6), 45.4 (NCH_3), 26.2 (C-5). IR (film) ν_{max} 1708, 1491, 1462, 1288, 1239, 1188, 1139, 1111, 1084, 1046, 1023, 998, 971, 754, 734, 717, 698. HRMS (ESI) (m/z) calcd. for $C_{21}H_{24}NO_3$ $[M + H]^+$: 338.1751; found 338.1753.

Phenyl(4-(4,4,5,5-tetramethyl-1,3,2-dioxaborolan-2-yl)phenyl)methyl 1-methyl-1,2,5,6-tetrahydropyridine-3-carboxylate (**37**). Following the general procedure on a 0.25 mmol scale, arecaidine was esterificated using alcohol **31**. Purification by flash column chromatography (0–6% MeOH in CH_2Cl_2) afforded the title compound **37** (51 mg, 78%) as a colorless oil. ^1H NMR (400 MHz, $CDCl_3$) δ 7.80 (m, 2H, Ph H-3,5), 7.35 (m, 2H, Ph H-2,6), 7.06 (m, 1H, H-4), 5.19 (s, 2H, OCH_2), 3.18 (m, 2H, H-2), 2.51 (m, 2H, H-6), 2.41 (s, 3H, NCH_3), 2.38 (m, 2H, H-5), 1.34 (s, 12H, $C(CH_3)_2$). ^{13}C NMR (125 MHz, $CDCl_3$) δ 165.4 (C=O), 139.1 (Ph C-1), 138.1 (C-4), 135.0 (Ph C-3,5), 128.72 (C-3, Ph C-4), 127.2 (Ph C-2,6), 83.8 ($\underline{C}(CH_3)_2$), 66.0 (OCH_2), 53.1 (C-2), 50.7 (C-6), 45.6 (NCH_3), 26.5 (C-5), 24.8 ($C(\underline{C}H_3)_2$). IR (film) ν_{max} 2977, 1712, 1398, 1359, 1322, 1289, 1262, 1141, 1088, 1046, 1022, 962, 859, 658. HRMS (ESI) (m/z) calcd. for $C_{20}H_{29}BNO_4$ $[M + H]^+$: 358.2184; found 358.2180.

2-(hydroxymethyl)phenyl 1-methyl-1,2,5,6-tetrahydropyridine-3-carboxylate (**44**). Following the general procedure on a 0.25 mmol scale, arecaidine was esterificated using alcohol **38**. Purification by flash column chromatography (0–12% MeOH in CH_2Cl_2) afforded the title compound **44** (36 mg, 58%) as a pale yellow oil. ^1H NMR (500 MHz, $CDCl_3$) δ 7.28 (m, 1H, Ph H-3), 7.26 (m, 1H, Ph H-5), 7.11 (m, 1H, H -4), 6.93 (m, 1H, Ph H-6), 6.90 (m, 1H, Ph H-4) 5.20 (s, 2H, OCH_2), 3.22 (m, 2H, H-2), 2.57 (m, 2H, H-6), 2.45 (s, 3H, NCH_3), 2.42 (m, 2H, H-5). ^{13}C NMR (125 MHz, $CDCl_3$) δ 167.4 (C=O), 155.6 (Ph C-1), 139.6 (C-4), 132.0 (Ph C-3), 131.0 (Ph C-5), 121.7 (Ph C-2), 120.3 (Ph C-4), 63.3 (OCH_2), 52.7 (C-2), 50.4 (C-6), 45.3 (NCH_3), 26.3 (C-5). IR (film) ν_{max} 1721, 1506, 1290, 1235, 1189, 1163, 1134, 1125, 1045, 1011, 993, 954, 877, 850, 814, 787, 733, 712. HRMS (ESI) (m/z) calcd. for $C_{14}H_{18}NO_3$ $[M + H]^+$: 248.1281; found 248.1283.

4-(hydroxymethyl)phenyl 1-methyl-1,2,5,6-tetrahydropyridine-3-carboxylate (**45**). Following the general procedure on a 0.25 mmol scale, arecaidine was esterificated using alcohol **39**. Purification by flash column chromatography (0–12% MeOH in CH_2Cl_2) afforded the title compound **45** (32 mg, 52%) as a pale yellow oil. ^1H NMR (500 MHz, $CDCl_3$) δ 7.39 (m, 2H, Ph H-3,5), 7.28 (m, 1H, H-4), 7.09 (m, 2H, Ph H-2,6), 4.69 (s, 2H, CH_2O), 3.40 (m, 1H, H-2), 2.71 (m, 2H, H-6), 2.57 (H-5), 2.55 (s, 3H, NCH_3). ^{13}C NMR (125 MHz, $CDCl_3$) δ 163.9 (C=O), 149.9 (Ph C-1), 139.6 (C-4), 138.8 (Ph C-4), 127.92 (C-3, Ph C-3,5), 121.6 (Ph C-2,6), 64.5 (CH_2O), 52.8 (C-2), 50.5 (C-6), 45.4 (NCH_3), 26.4 (C-5). IR (film) ν_{max} 1706, 1457, 1368, 1262, 1188, 1136, 1102, 1023, 757, 720. HRMS (ESI) (m/z) calcd. for $C_{14}H_{18}NO_3$ $[M + H]^+$: 248.1281; found 262.1287.

4-(hydroxy(phenyl)methyl)phenyl 1-methyl-1,2,5,6-tetrahydropyridine-3-carboxylate (**46**). Following the general procedure on a 0.25 mmol scale, arecaidine was esterificated using alcohol **30**. Purification by flash column chromatography (0–12% MeOH in CH_2Cl_2) afforded the title compound **46** (50 mg, 62%) as a pale yellow oil. ^1H NMR (400 MHz, $CDCl_3$) δ 7.37 (m, 2H, OPh H-3,5), 7.36 (m, 2H, H_5Ph H-2,6), 7.32 (m, 2H, H_5Ph H-3,5), 7.25 (m, 1H, H_5Ph H-4), 7.18 (m, 1H, H-4) 7.05 (m, 2H, OPh H-2,6), 5.77 (s, 1H, $C\underline{H}OH$), 3.89 (br s, 1H, $CHO\underline{H}$), 3.12 (m, 2H, H-2), 2.52 (m, 2H, H-6), 2.38 (s, 3H, NCH_3), 2.42 (m, 2H, H-5). ^{13}C NMR (100 MHz, $CDCl_3$) δ 163.8 (C=O), 149.8 (OPh C-1), 143.8 (H_5Ph C-1), 141.7 (OPh C-4), 139.6 (C-4), 128.4 (H_5Ph C-3,5), 128.2 (C-3), 127.5 (OPh C-3,5), 126.4 (H_5Ph C-2,6), 121.4 (OPh C-2,6), 75.4

(CHOH), 52.8 (C-2), 50.5 (C-6), 45.5 (NCH_3), 26.5 (C-5). IR (film) ν_{max} 1723, 1504, 1452, 1291, 1263, 1200, 1164, 1135, 1125, 1076, 1066, 1045, 1013, 736, 699. HRMS (ESI) (m/z) calcd. for $C_{20}H_{22}NO_3$ $[M + H]^+$: 324.1594; found 324.11602.

2D Pharmacophores and Docking Pose Overlays

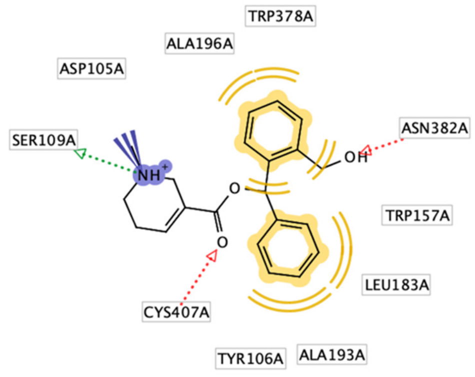

(**a**)

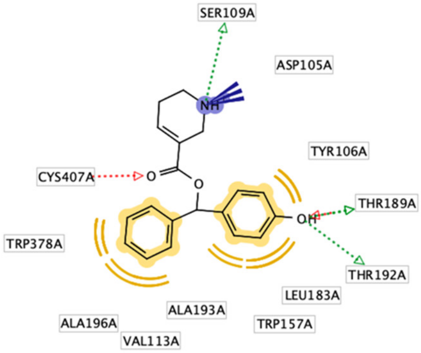

(**b**)

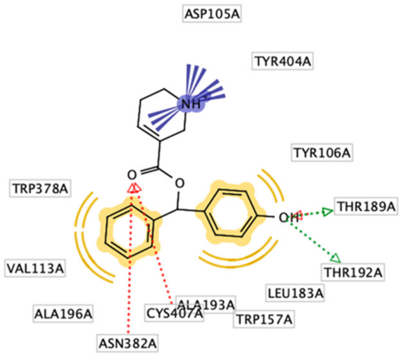

(**c**)

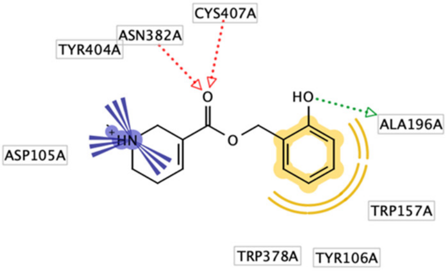

(**d**)

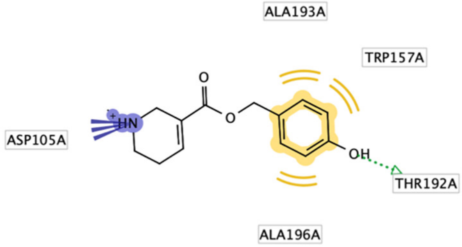

(**e**)

Figure S3. 2D pharmacophores for hydroxylated arecaidine esters, which could not be synthesized, in the orthosteric binding site of M_1 (PDB 5CXV) with interacting amino acid residues and key interactions highlighted. In case of chiral compounds only one enantiomer is shown: (**a**) 2D pharmacophore of (*S*)-**10a**; (**b**) 2D pharmacophore of (*S*)-**17a**; (**c**) 2D pharmacophore of (*S*)-**17c**; (**d**) 2D pharmacophore of **18a**; (**e**) 2D pharmacophore of **18c**.

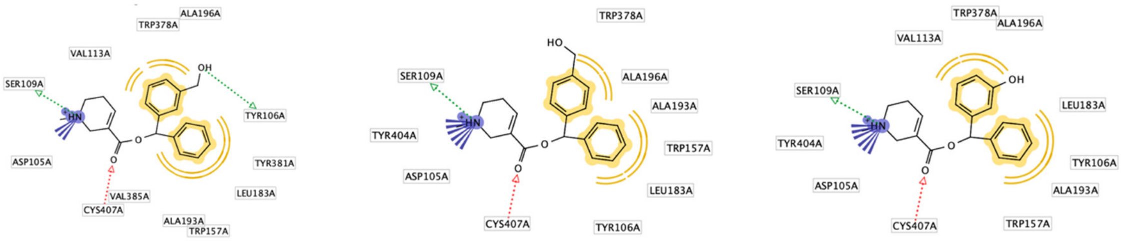

(**a**) (**b**) (**c**)

Figure S4. 2D pharmacophores for selected hydroxylated arecaidine esters in the orthosteric binding site of M_1 (PDB 5CXV) with interacting amino acid residues and key interactions highlighted: (**a**) 2D pharmacophore of (*S*)-**10b**; (**b**) 2D pharmacophore of (*S*)-**10c**; (**c**) 2D pharmacophore of (*S*)-**17b**.

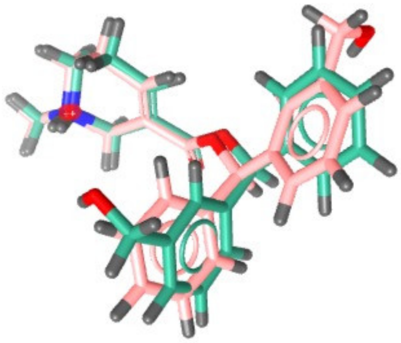

(**a**)

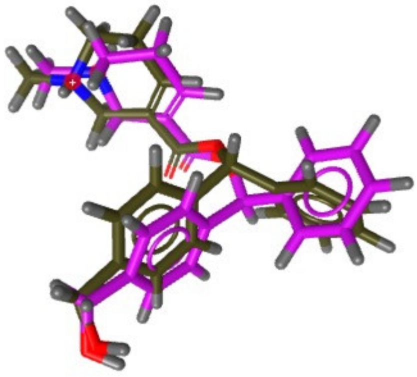

(**b**)

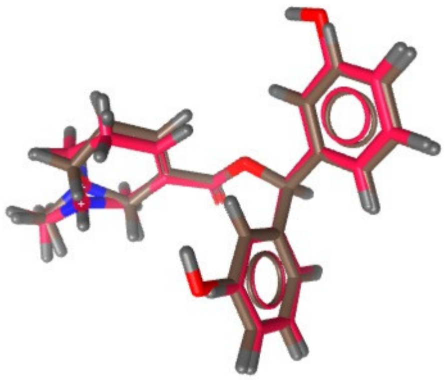

(**c**)

Figure S5. Superimposed docking poses for the enantiomeric pairs of **10b**, **10c**, and **17b**. (**a**) docking poses of (*R*)- and (*S*)-**10b**; (**b**) docking poses of (*R*)- and (*S*)-**10c**; (**c**) docking poses of (*R*)- and (*S*)-**17b.**

Single-concentration Radioligand Binding Assay

Percent displacements of compounds that were excluded from full-range concentration dependent radioligand binding assays to determine their affinities are displayed in Table S1.

Table S1. Percent displacements of [^{3}H]NMS binding on cell membranes derived from CHO-K1 cells expressing *h*Mx receptors at ligand concentrations corresponding to a K_i value of 1 μM according to the Cheng-Prusoff Equation.

	Displacement[1] ± SD (%)				
**Cmpd.**	***h*M$_1$**	***h*M$_2$**	***h*M$_3$**	***h*M$_4$**	***h*M$_5$**
**8a**	66 ± 5	43 ± 2	72 ± 4	56 ± 9	72 ± 3
**8b**	31 ± 3	23 ± 4	34 ± 4	22 ± 1	29 ± 6
**22**	61 ± 2	28 ± 5	33 ± 8	51 ± 5	72 ± 5
**27**	37 ± 6	34 ± 7	51 ± 9	46 ± 3	65 ± 11

[1] Each value is the mean of two independent experiments carried out in triplicate.

Purity measured by HPLC

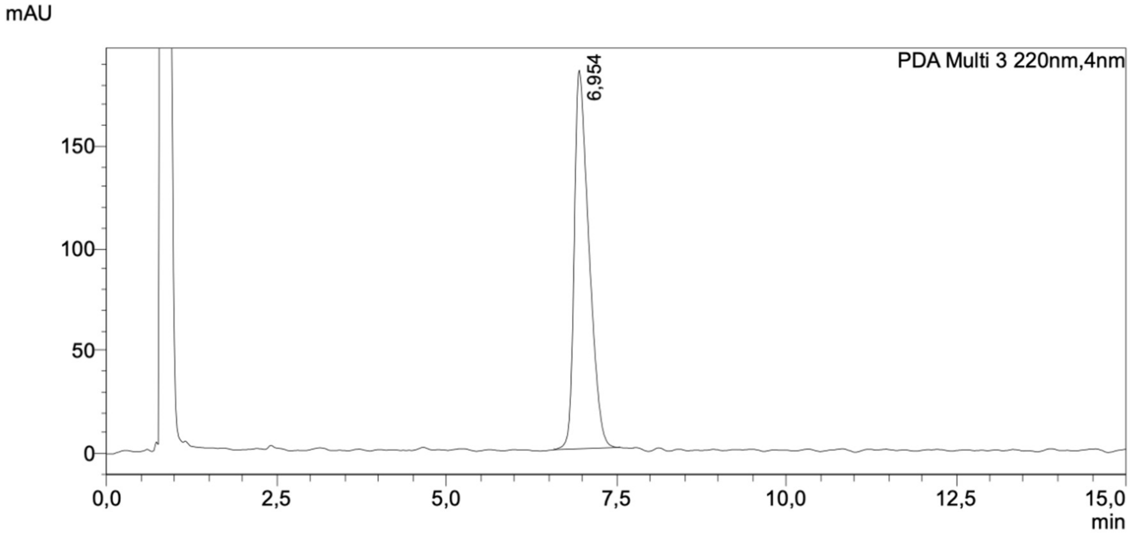

<Peak Table>

PDA Ch3 220nm

Peak#	Ret. Time	Area	Height	Area%	Conc.	Mark
1	6,954	2780029	184587	100,000	0,000	M
Total		2780029	184587	100,000		

Figure S6. Isocratic HPLC chromatogram of **8a**. 30% ACN in H_2O, 0.1% TFA.

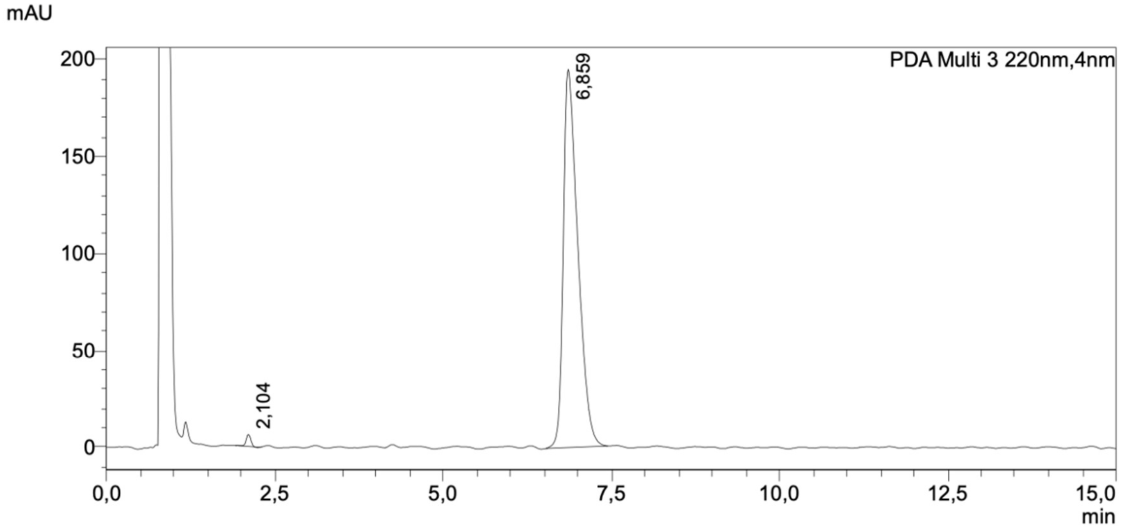

<Peak Table>

PDA Ch3 220nm

Peak#	Ret. Time	Area	Height	Area%	Conc.	Mark
1	2,104	27271	6157	0,922	0,000	M
2	6,859	2929135	194832	99,078	0,000	M
Total		2956406	200989	100,000		

Figure S7. Isocratic HPLC chromatogram of **8b**. 30% ACN in H_2O, 0.1% TFA.

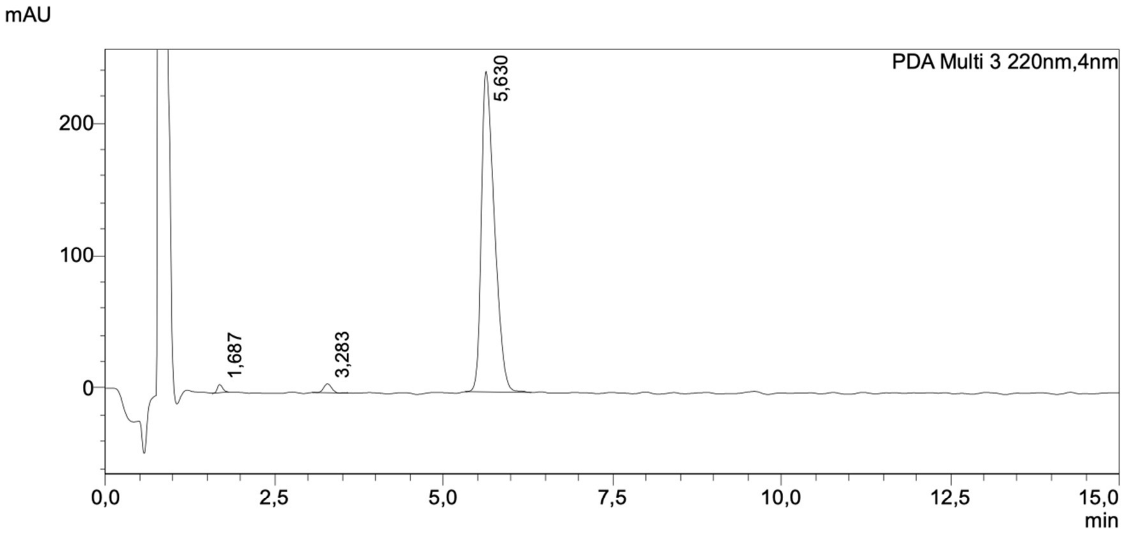

<Peak Table>

PDA Ch3 220nm

Peak#	Ret. Time	Area	Height	Area%	Conc.	Mark
1	1,687	32861	6112	0,988	0,000	M
2	3,283	52592	6629	1,581	0,000	M
3	5,630	3240620	241559	97,431	0,000	M
Total		3326072	254300	100,000		

Figure S8. Isocratic HPLC chromatogram of **8c**. 30% ACN in H_2O, 0.1% TFA.

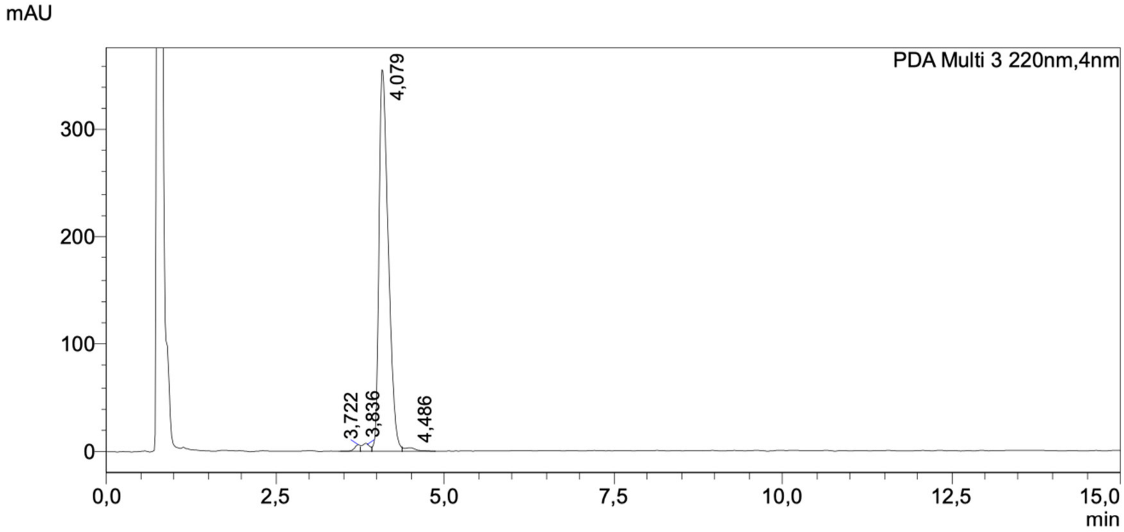

<Peak Table>

PDA Ch3 220nm

Peak#	Ret. Time	Area	Height	Area%	Conc.	Mark
1	3,722	36495	5892	1,030	0,000	M
2	3,836	51184	7302	1,445	0,000	V M
3	4,079	3415842	354996	96,440	0,000	V M
4	4,486	38430	3157	1,085	0,000	V M
Total		3541951	371347	100,000		

Figure S9. Isocratic HPLC chromatogram of **10b**. 30% ACN in H_2O, 0.1% TFA.

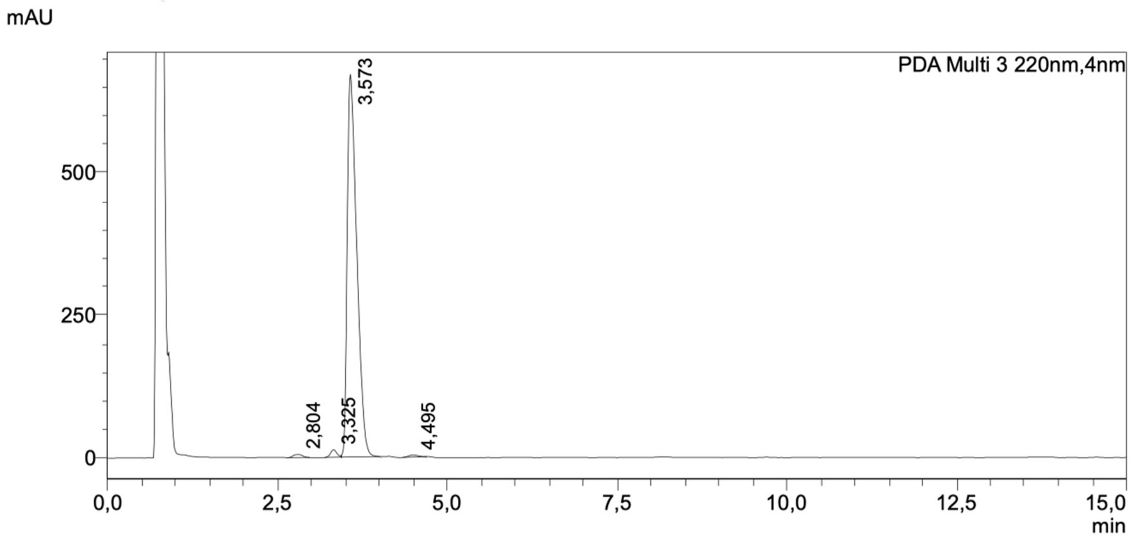

<Peak Table>

PDA Ch3 220nm

Peak#	Ret. Time	Area	Height	Area%	Conc.	Mark
1	2,804	50880	5233	0,758	0,000	M
2	3,325	86345	13190	1,287	0,000	M
3	3,573	6540911	670623	97,463	0,000	V M
4	4,495	33022	3532	0,492	0,000	M
Total		6711158	692577	100,000		

Figure S10. Isocratic HPLC chromatogram of **10c**. 30% ACN in H_2O, 0.1% TFA.

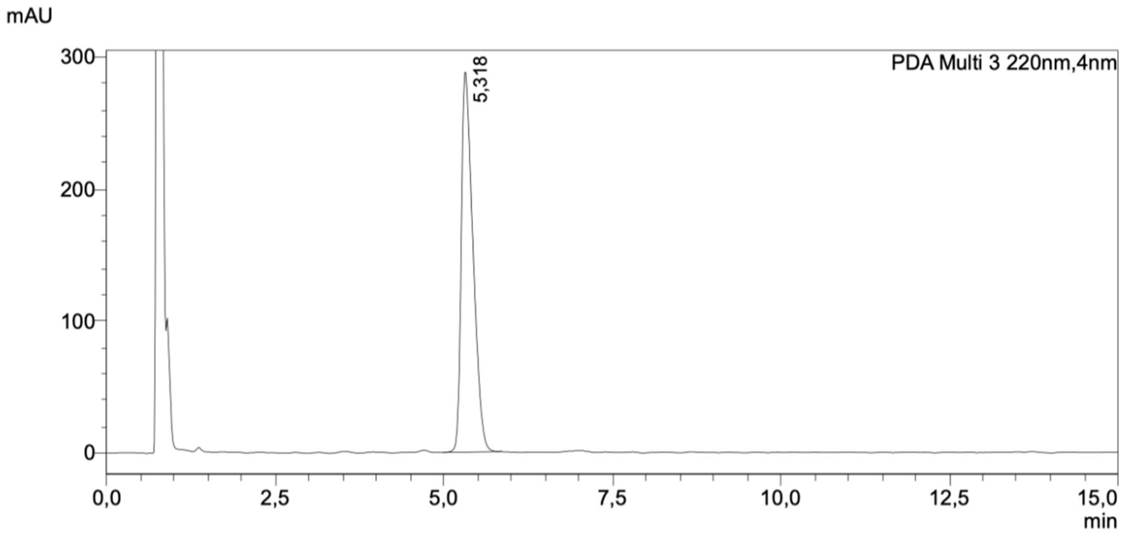

<Peak Table>

PDA Ch3 220nm

Peak#	Ret. Time	Area	Height	Area%	Conc.	Mark
1	5,318	3470597	287795	100,000	0,000	M
Total		3470597	287795	100,000		

Figure S11. Isocratic HPLC chromatogram of **17b**. 30% ACN in H_2O, 0.1% TFA.

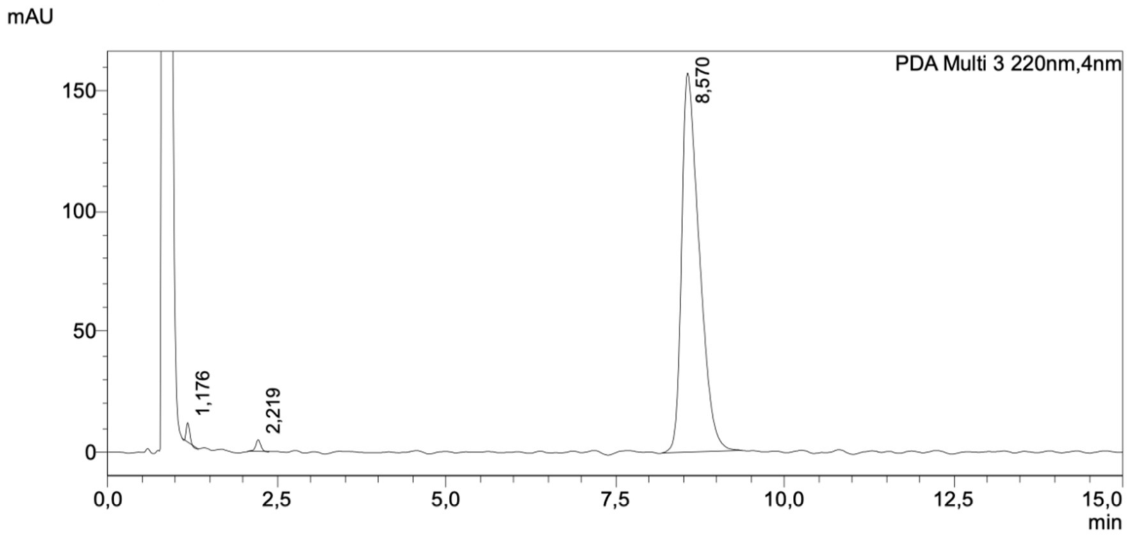

<Peak Table>

PDA Ch3 220nm

Peak#	Ret. Time	Area	Height	Area%	Conc.	Mark
1	1,176	24134	8058	0,829	0,000	M
2	2,219	25492	4845	0,876	0,000	M
3	8,570	2861319	157385	98,295	0,000	M
Total		2910945	170289	100,000		

Figure S12. Isocratic HPLC chromatogram of **18b**. 30% ACN in H_2O, 0.1% TFA.

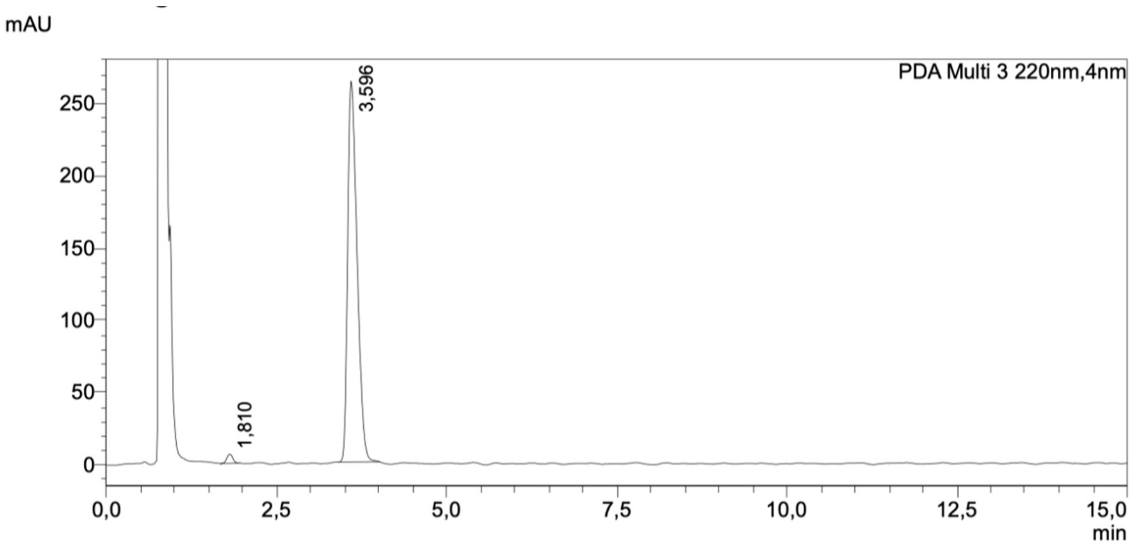

<Peak Table>

PDA Ch3 220nm

Peak#	Ret. Time	Area	Height	Area%	Conc.	Mark
1	1,810	37936	6329	1,476	0,000	M
2	3,596	2532246	263217	98,524	0,000	M
Total		2570182	269547	100,000		

Figure S13. Isocratic HPLC chromatogram of **22**. 30% ACN in H_2O, 0.1% TFA.

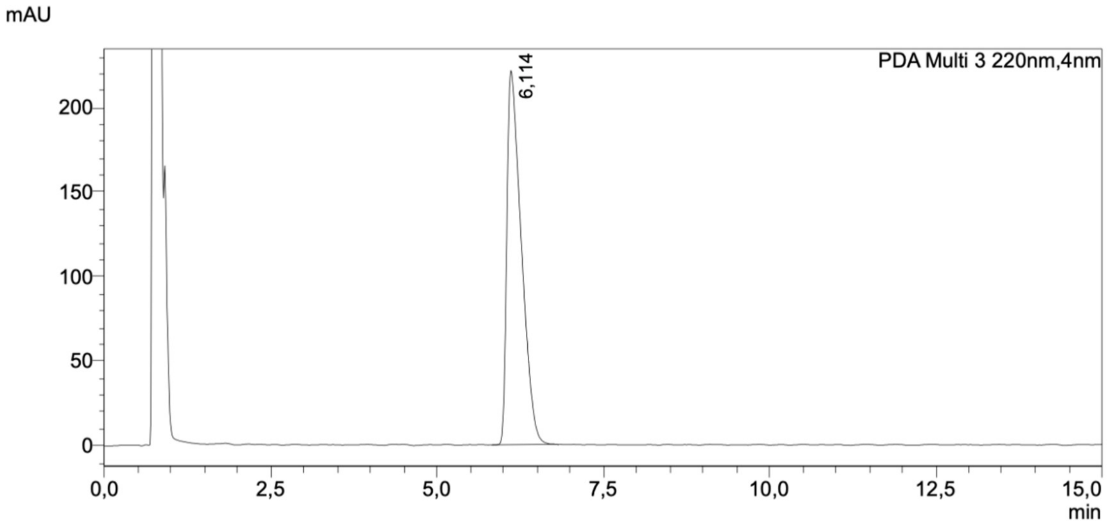

<Peak Table>

PDA Ch3 220nm

Peak#	Ret. Time	Area	Height	Area%	Conc.	Mark
1	6,114	3349684	221253	100,000	0,000	M
Total		3349684	221253	100,000		

Figure S14. Isocratic HPLC chromatogram of **25**. 30% ACN in H_2O, 0.1% TFA.

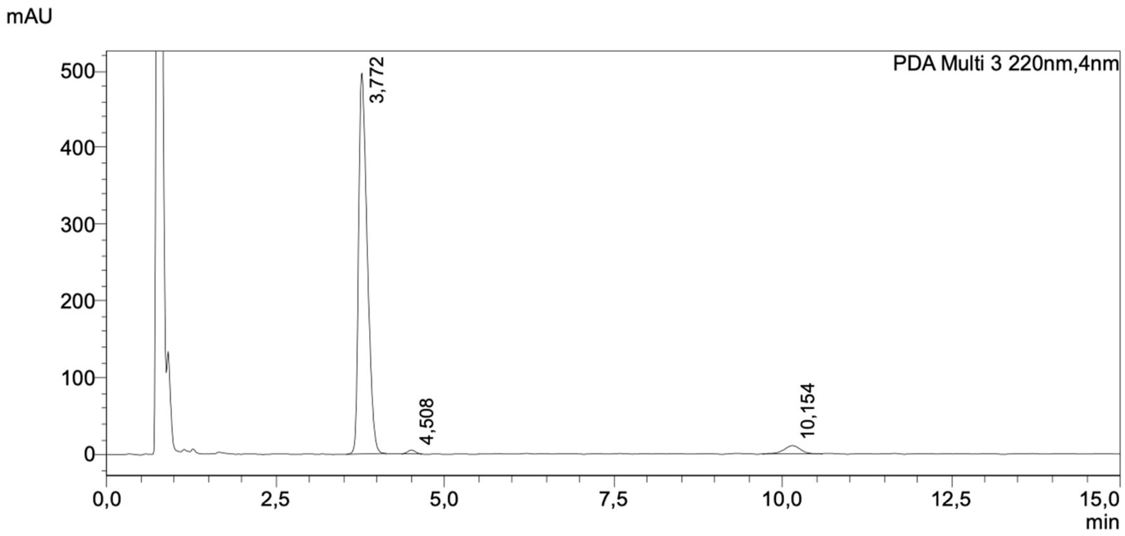

<Peak Table>

PDA Ch3 220nm

Peak#	Ret. Time	Area	Height	Area%	Conc.	Mark
1	3,772	4438263	496541	95,374	0,000	M
2	4,508	40438	4967	0,869	0,000	M
3	10,154	174840	10517	3,757	0,000	M
Total		4653542	512025	100,000		

Figure S15. Isocratic HPLC chromatogram of **27**. 30% ACN in H_2O, 0.1% TFA.

NMR Spectra

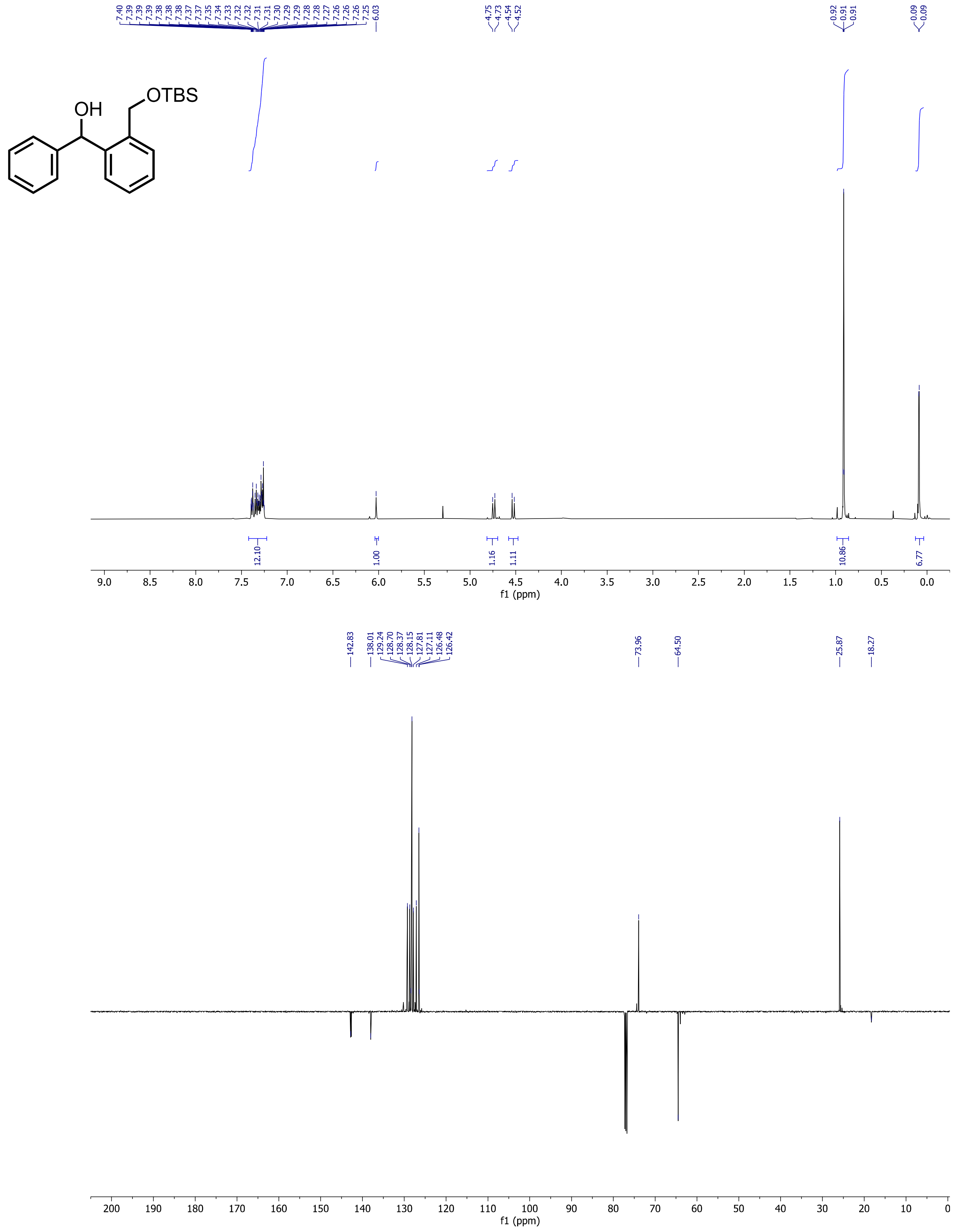

Figure S16. ^{1}H and ^{13}C NMR spectra of **6a**.

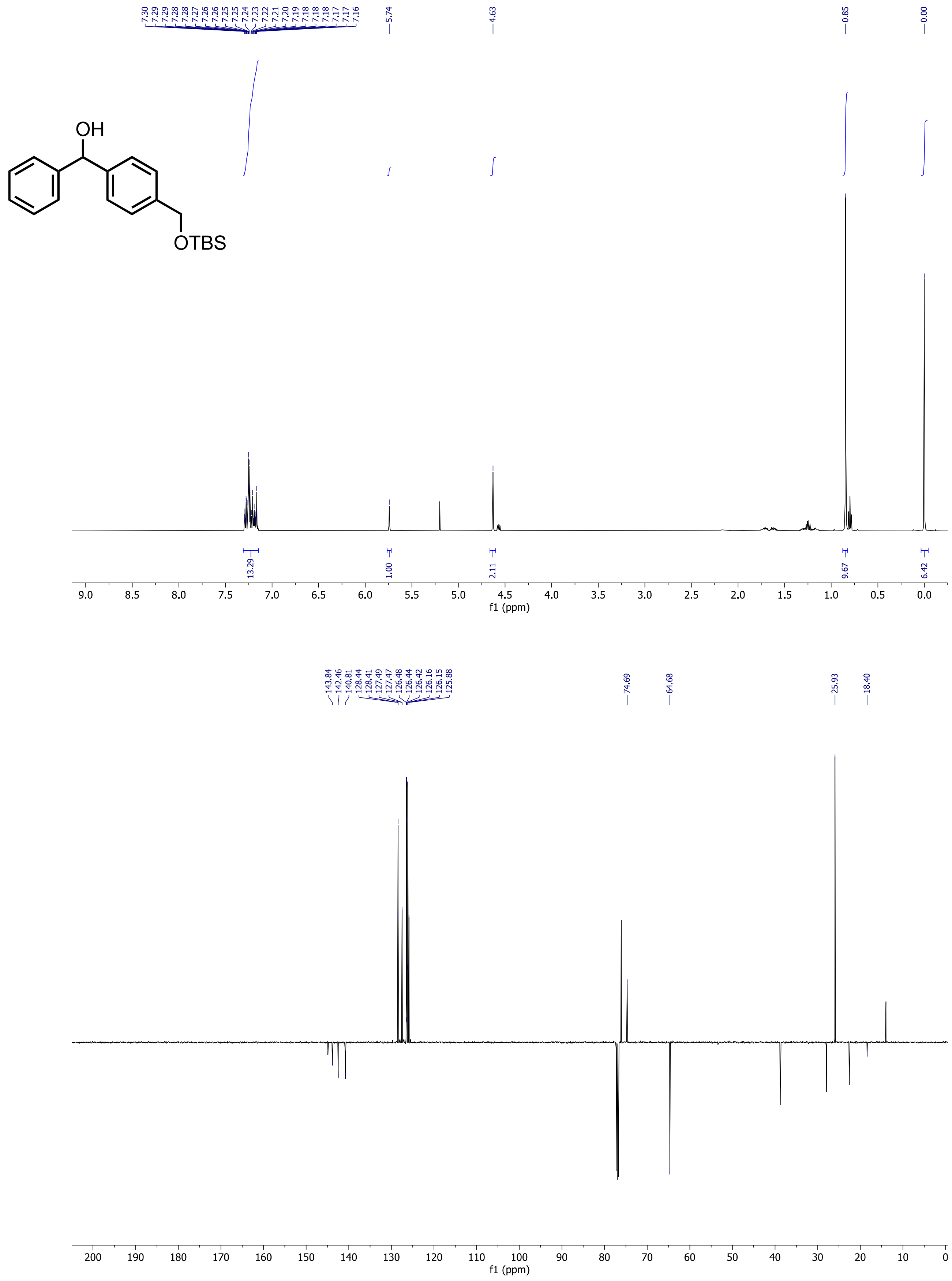

Figure S17. ^{1}H and ^{13}C NMR spectra of **6c**.

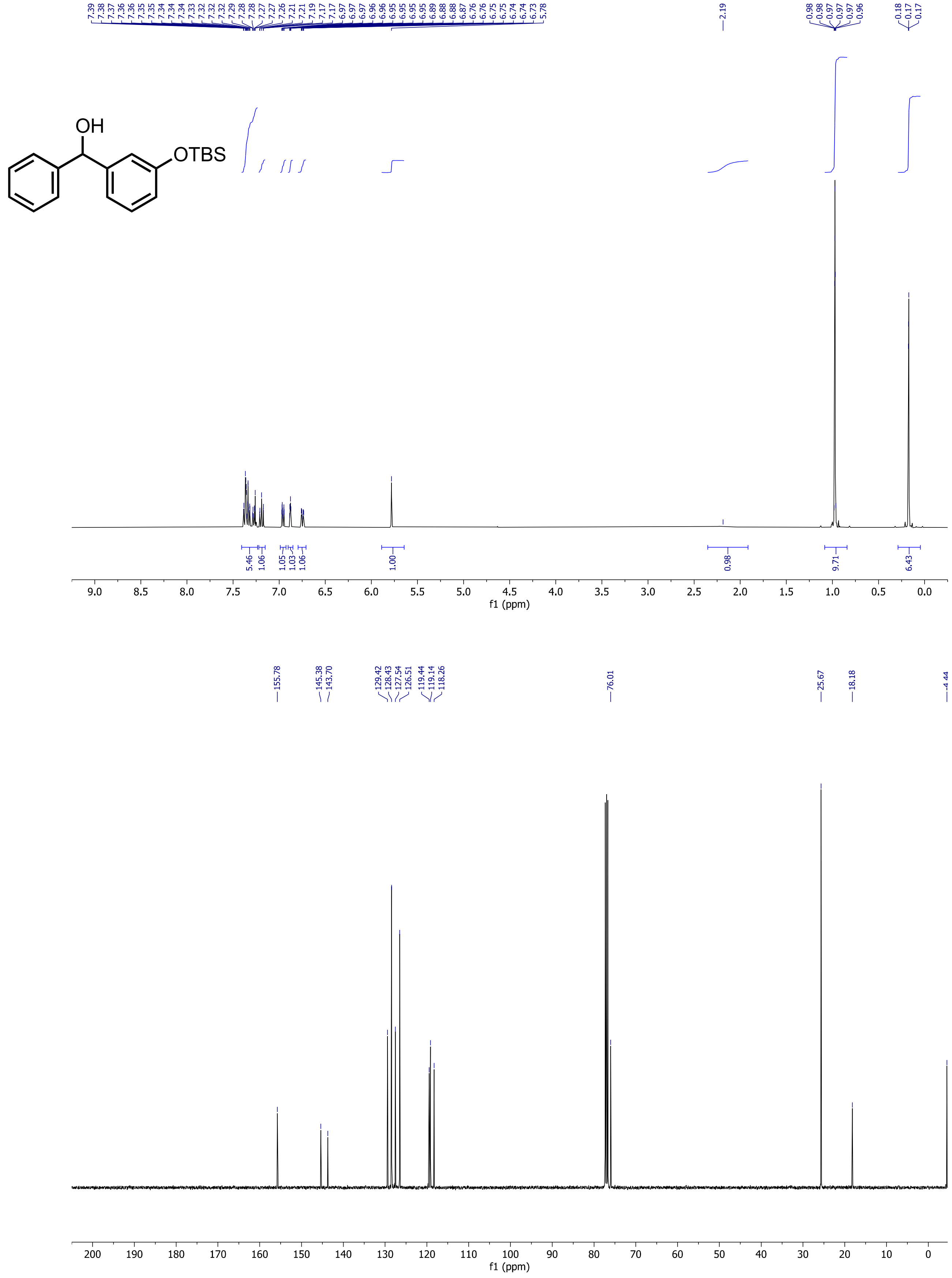

Figure S18. ^1H and ^{13}C NMR spectra of **13b**.

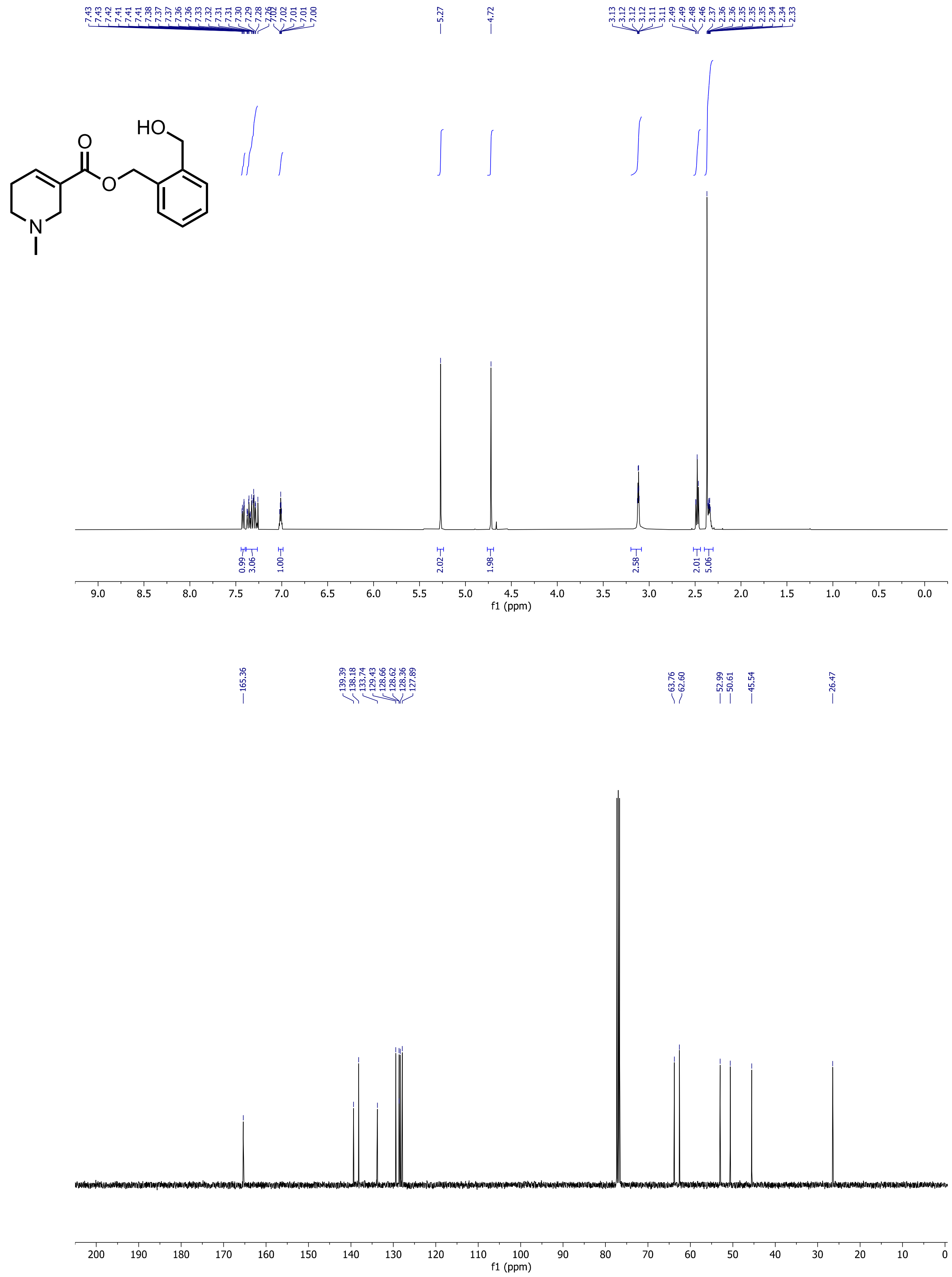

Figure S19. ^{1}H and ^{13}C NMR spectra of **8a**.

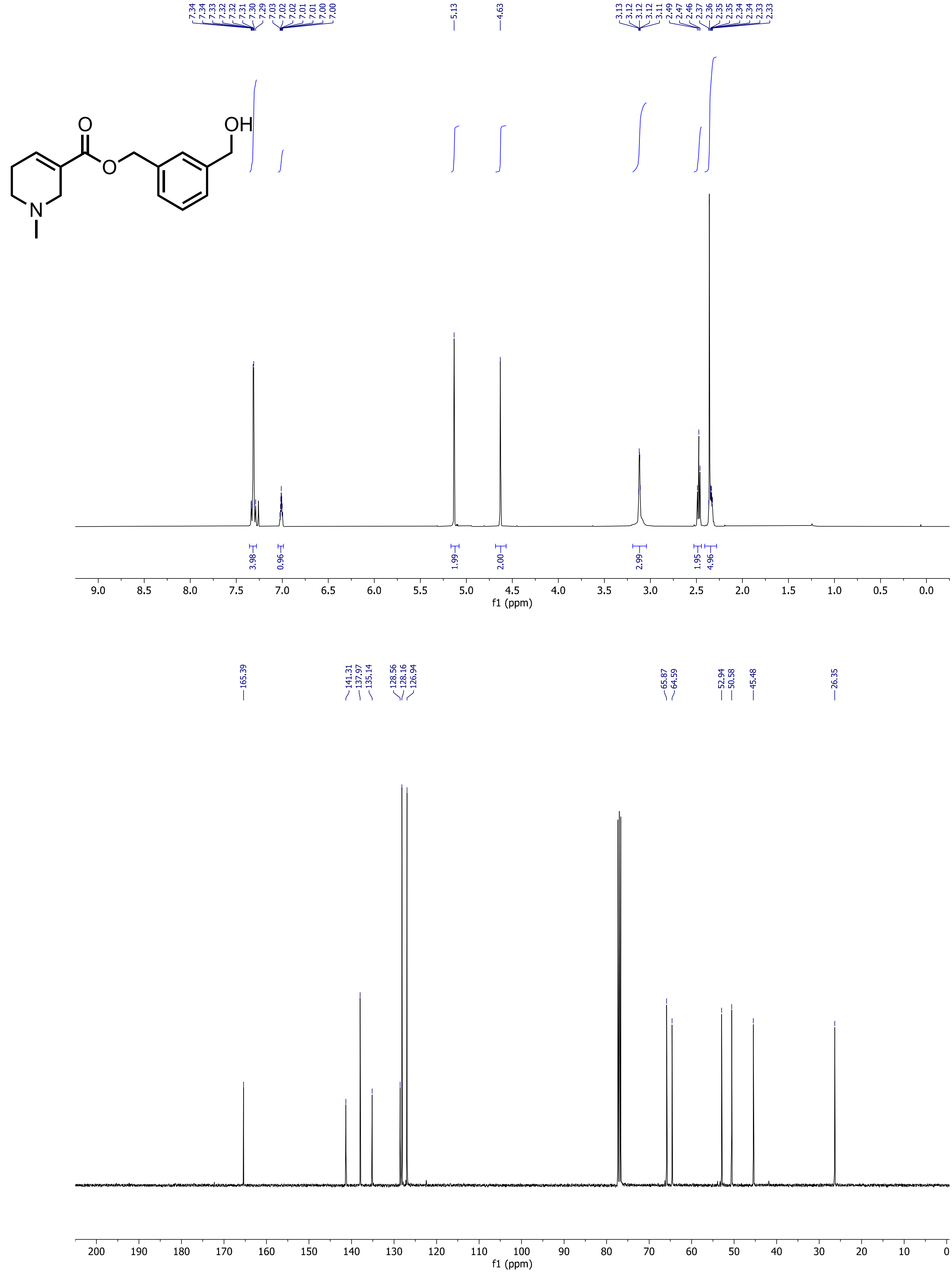

Figure S20. ^{1}H and ^{13}C NMR spectra of **8b**.

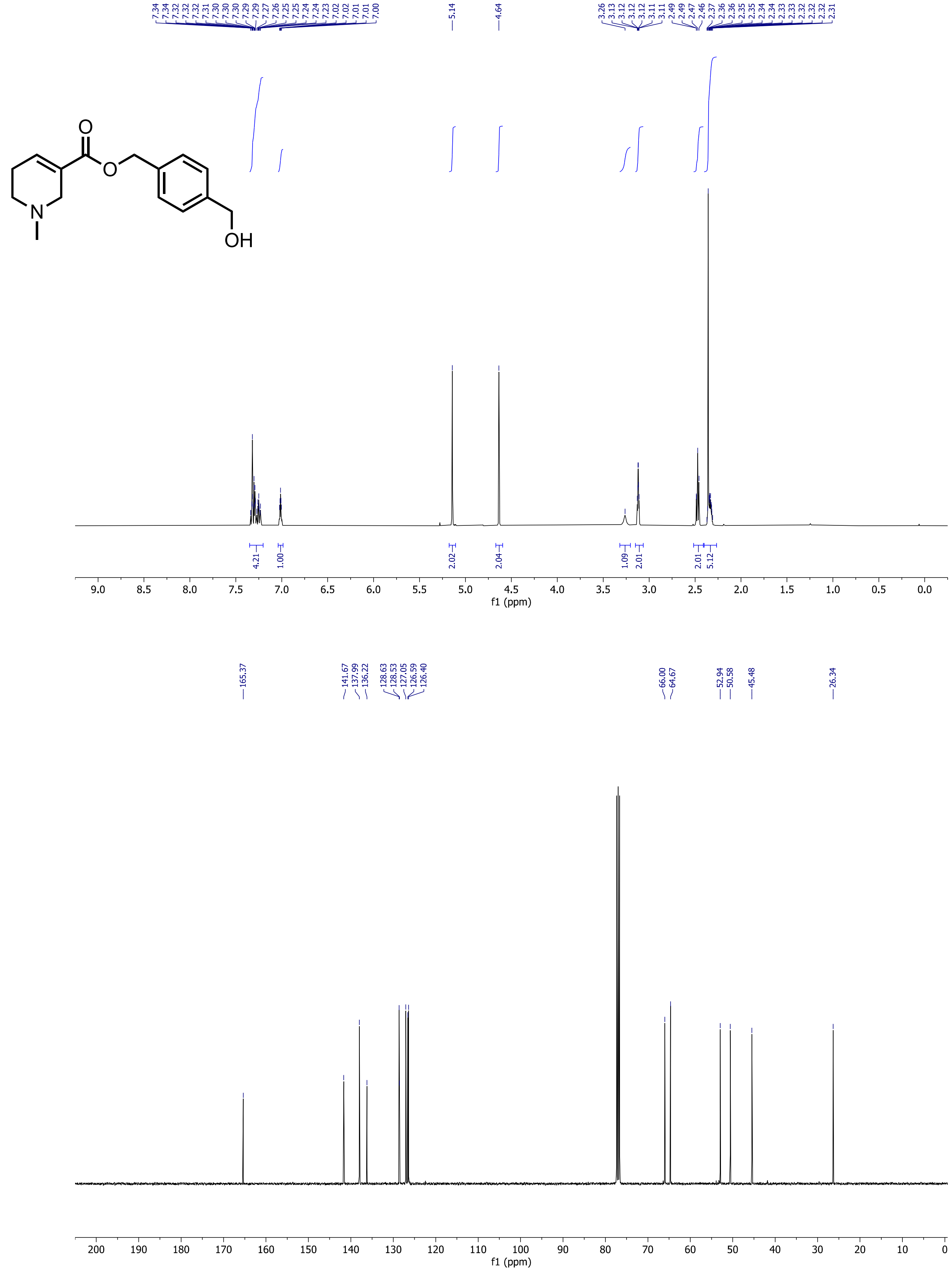

Figure S21. ^{1}H and ^{13}C NMR spectra of **8c**.

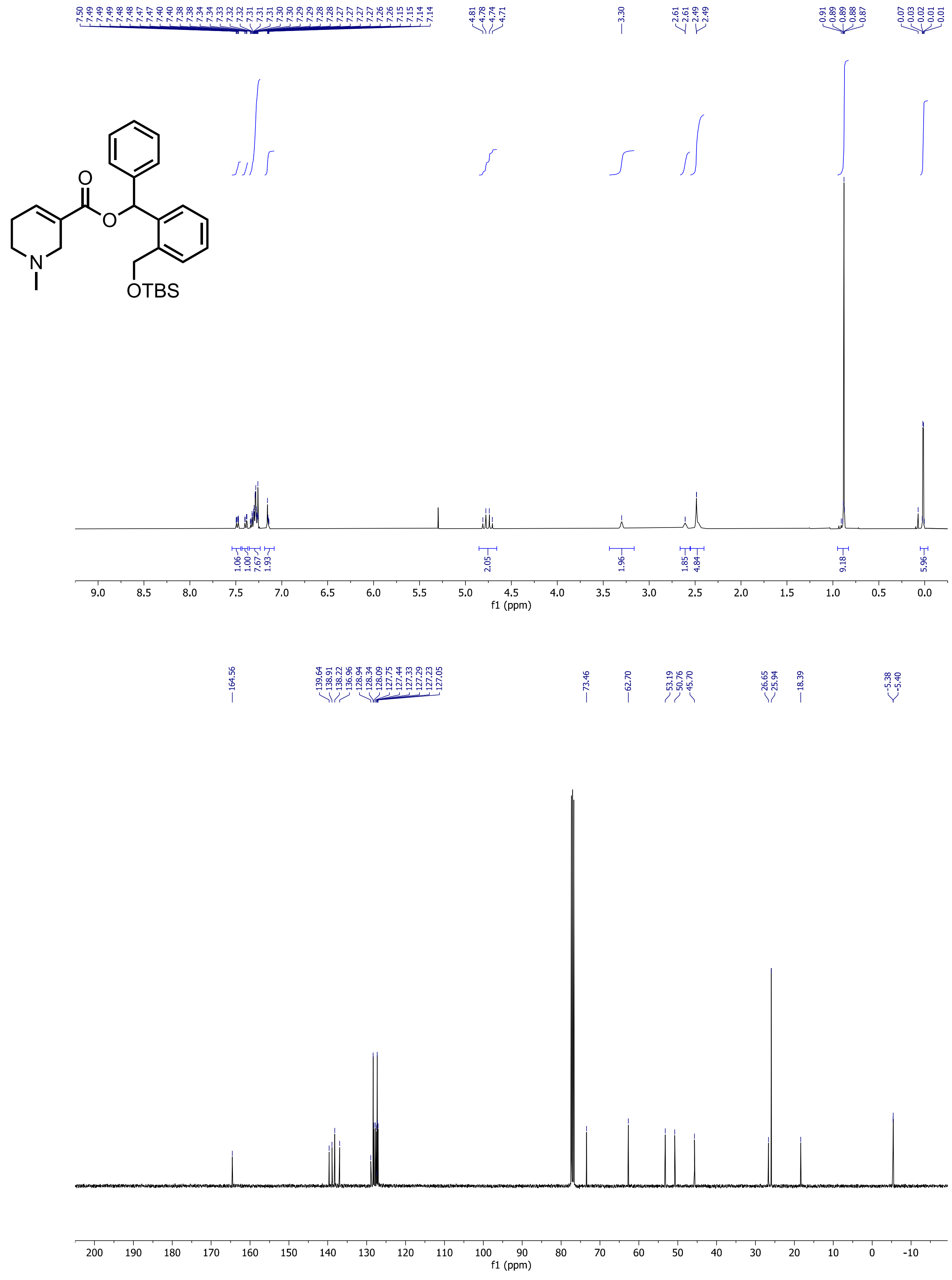

Figure S22. ^{1}H and ^{13}C NMR spectra of **9a**.

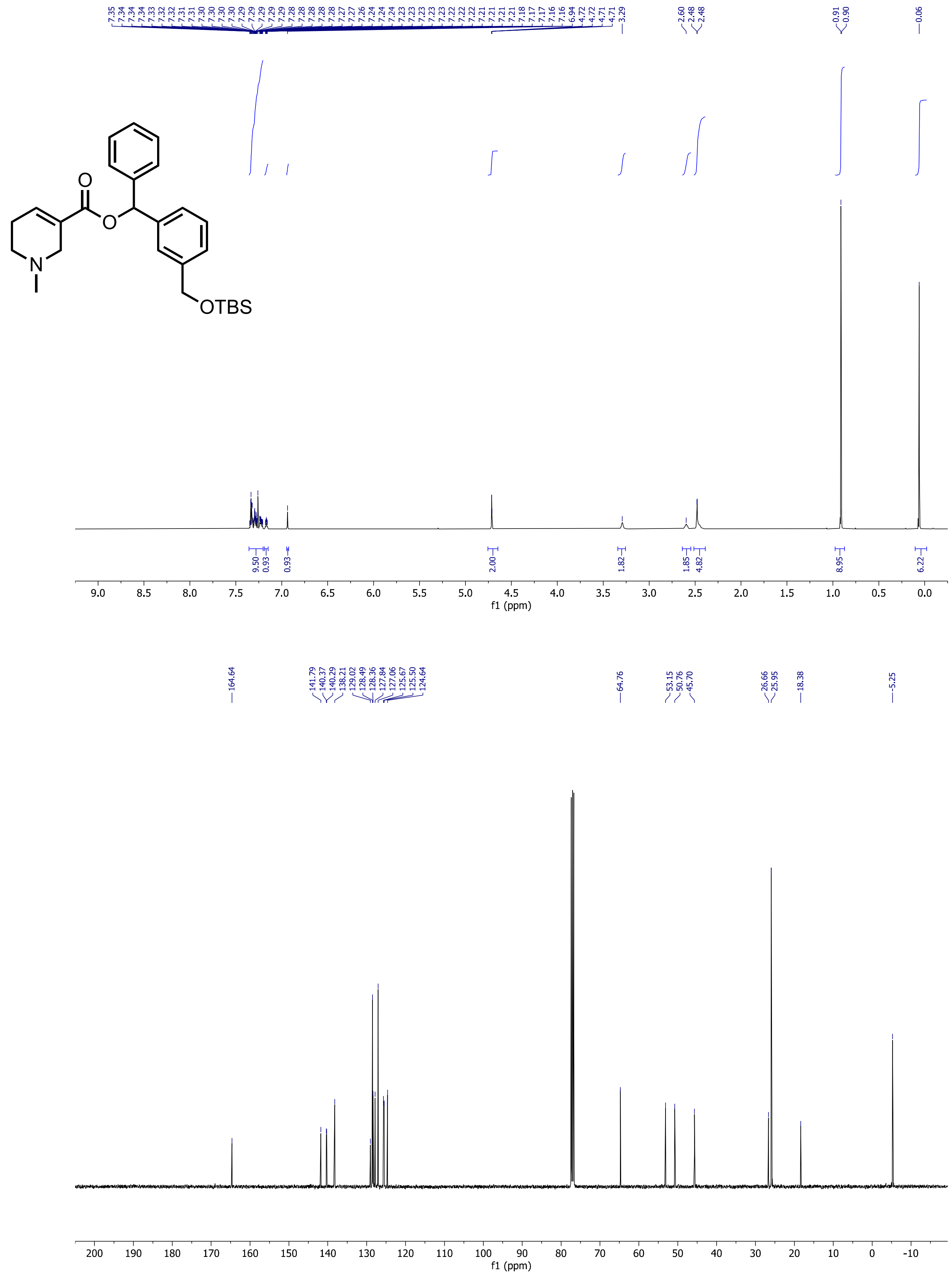

Figure S23. ^{1}H and ^{13}C NMR spectra of **9b**.

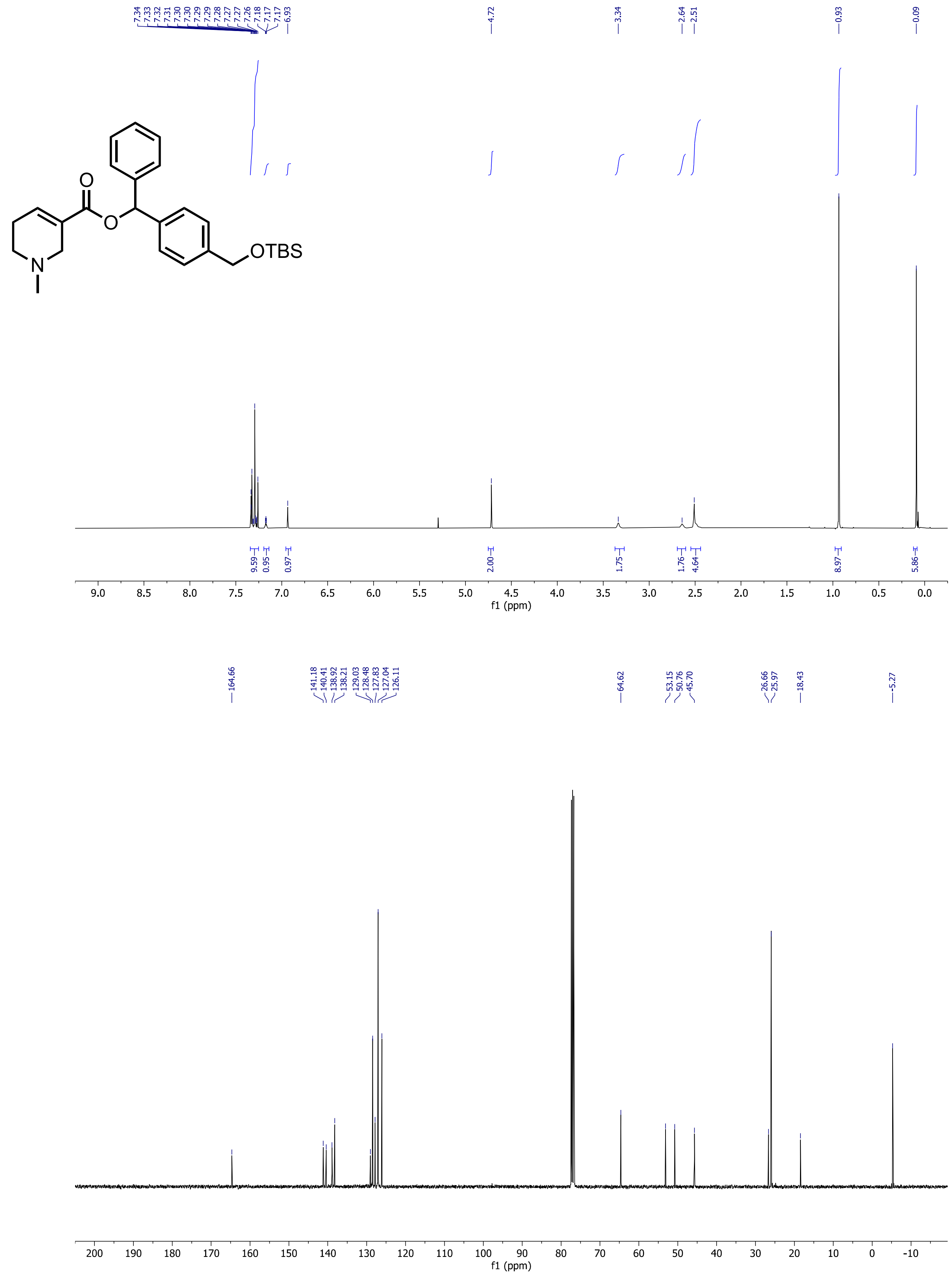

Figure S24. ^{1}H and ^{13}C NMR spectra of **9c**.

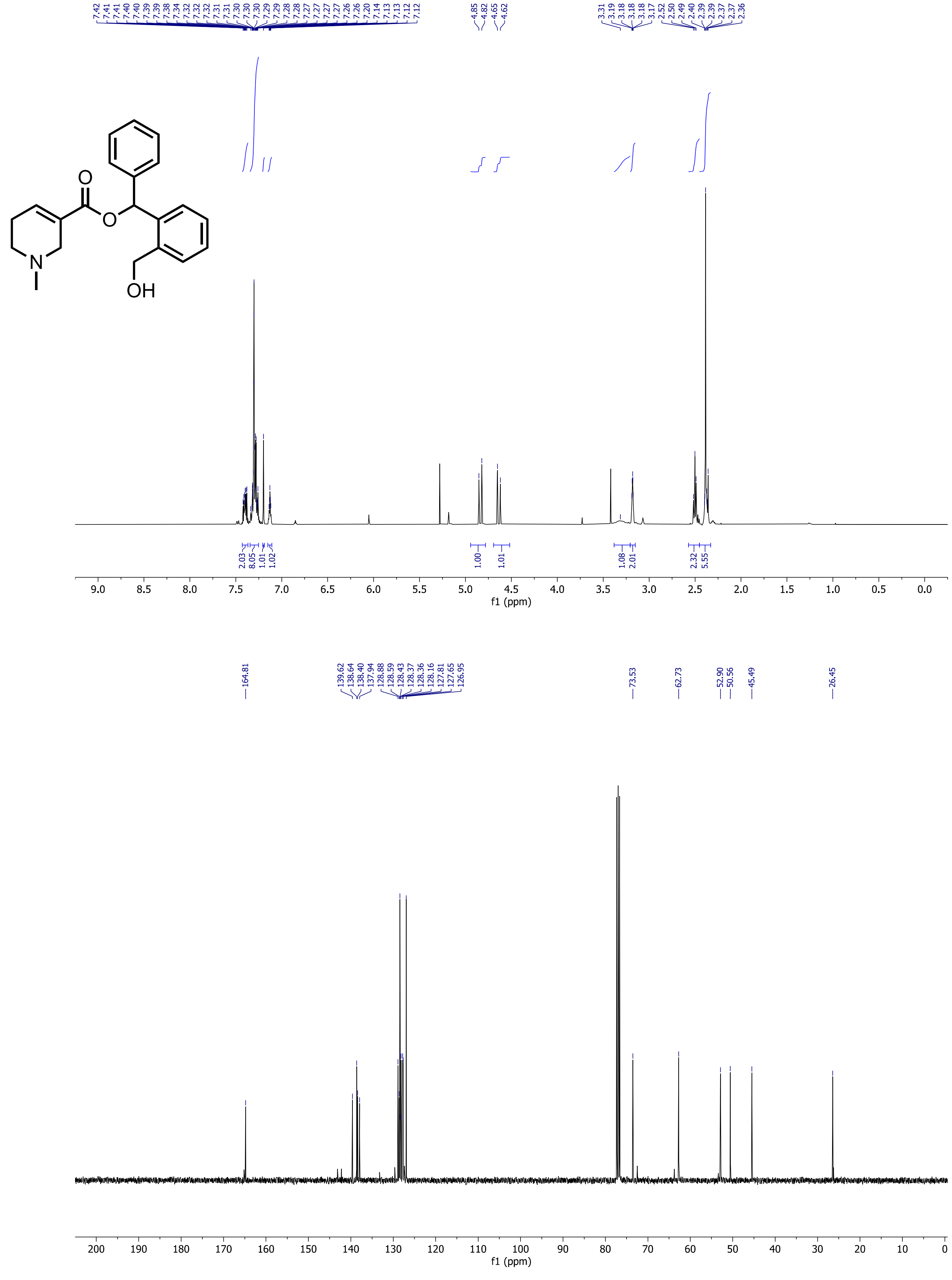

Figure S25. ^{1}H and ^{13}C NMR spectra of **10a**.

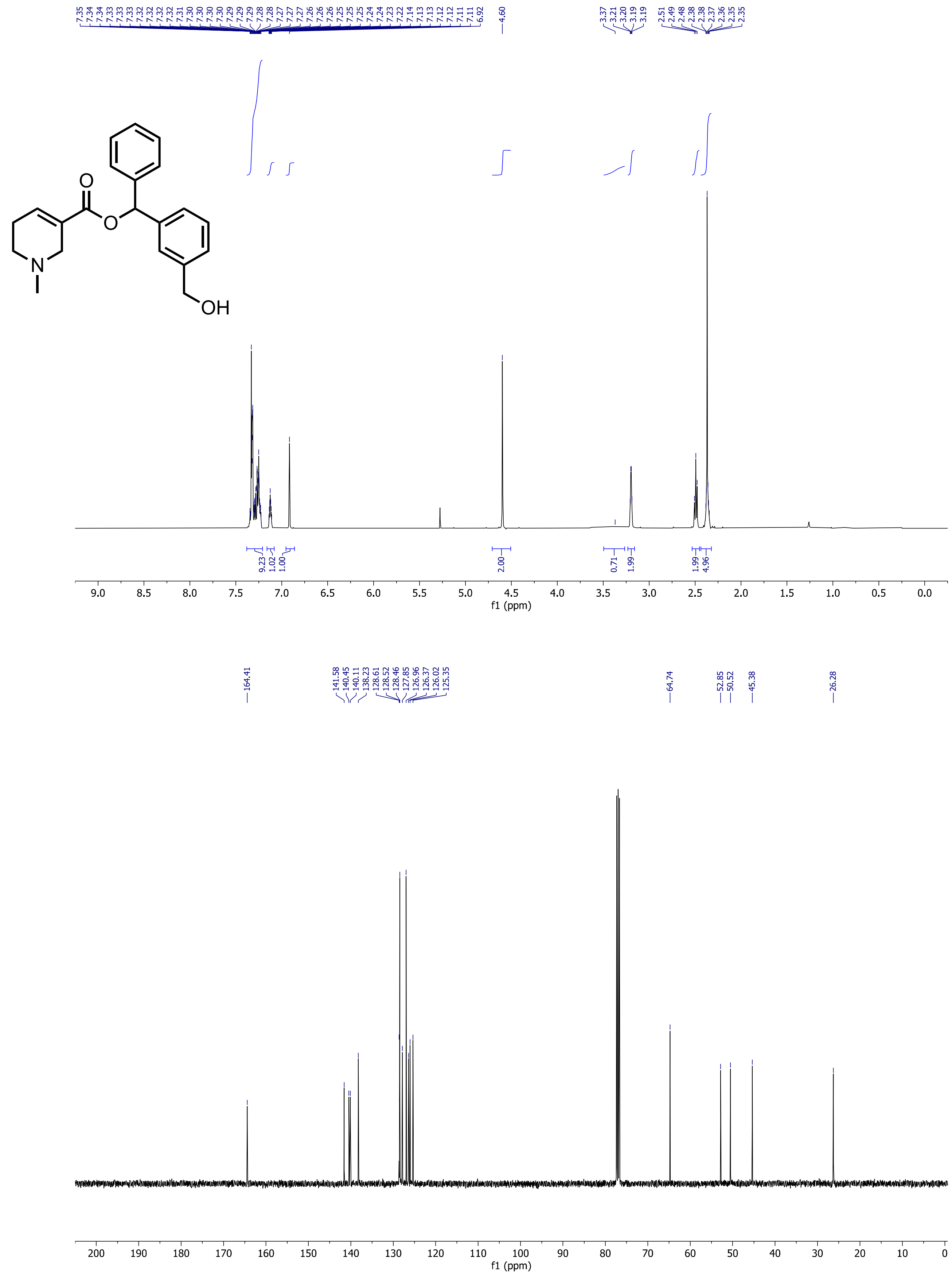

Figure S26. ^{1}H and ^{13}C NMR spectra of **10b**.

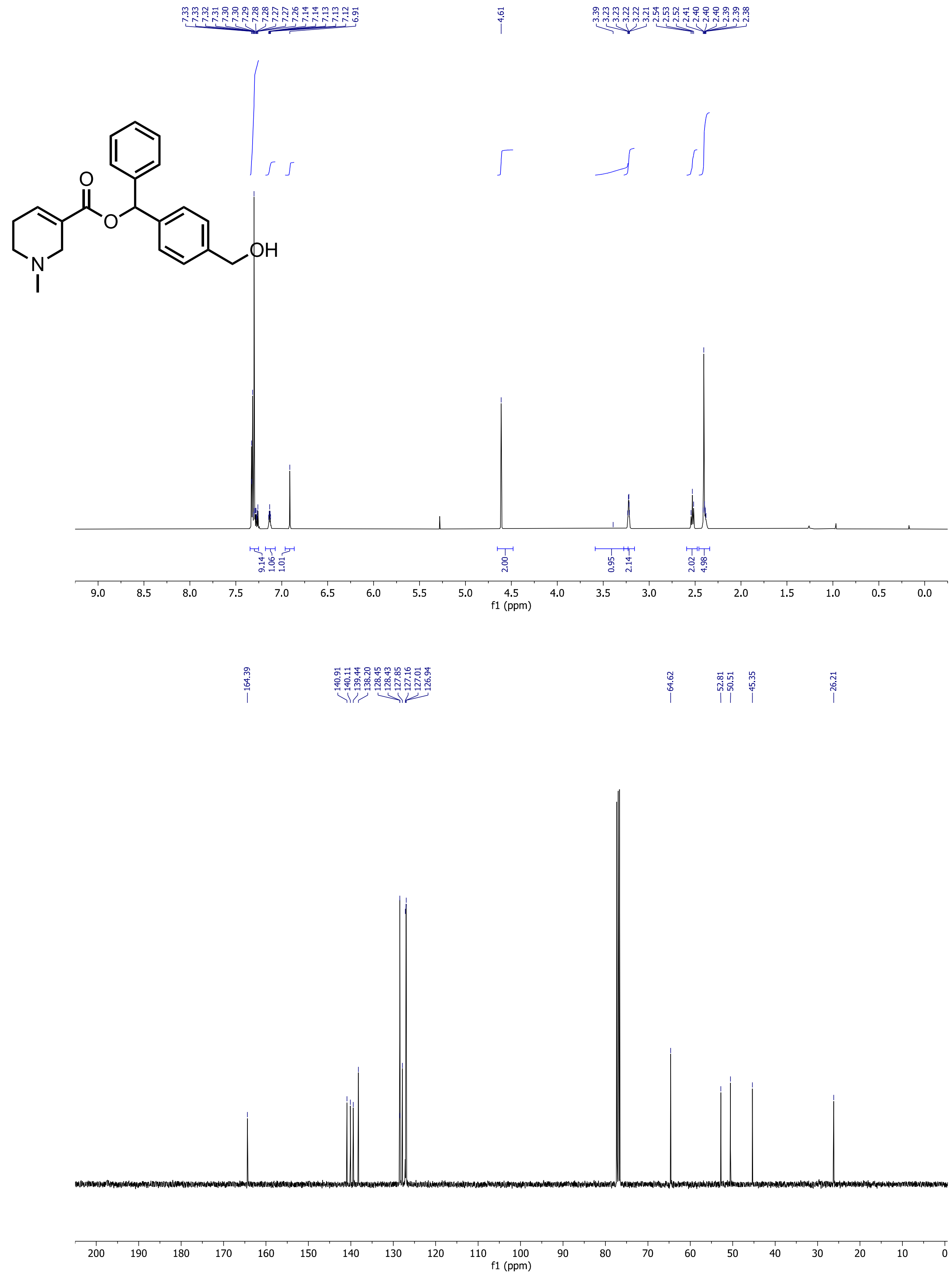

Figure S27. ^{1}H and ^{13}C NMR spectra of **10c**.

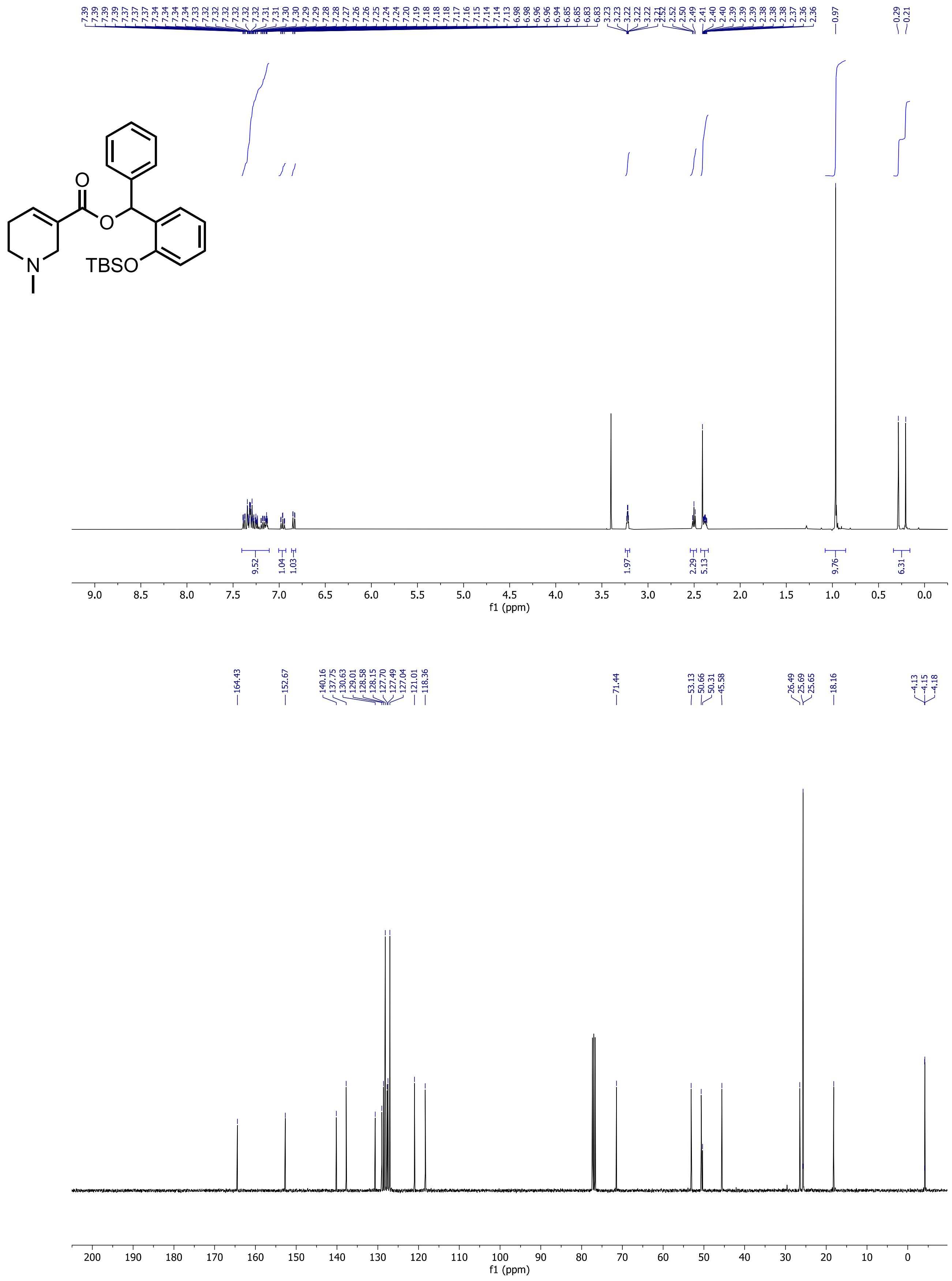

Figure S28. ^{1}H and ^{13}C NMR spectra of **15a**.

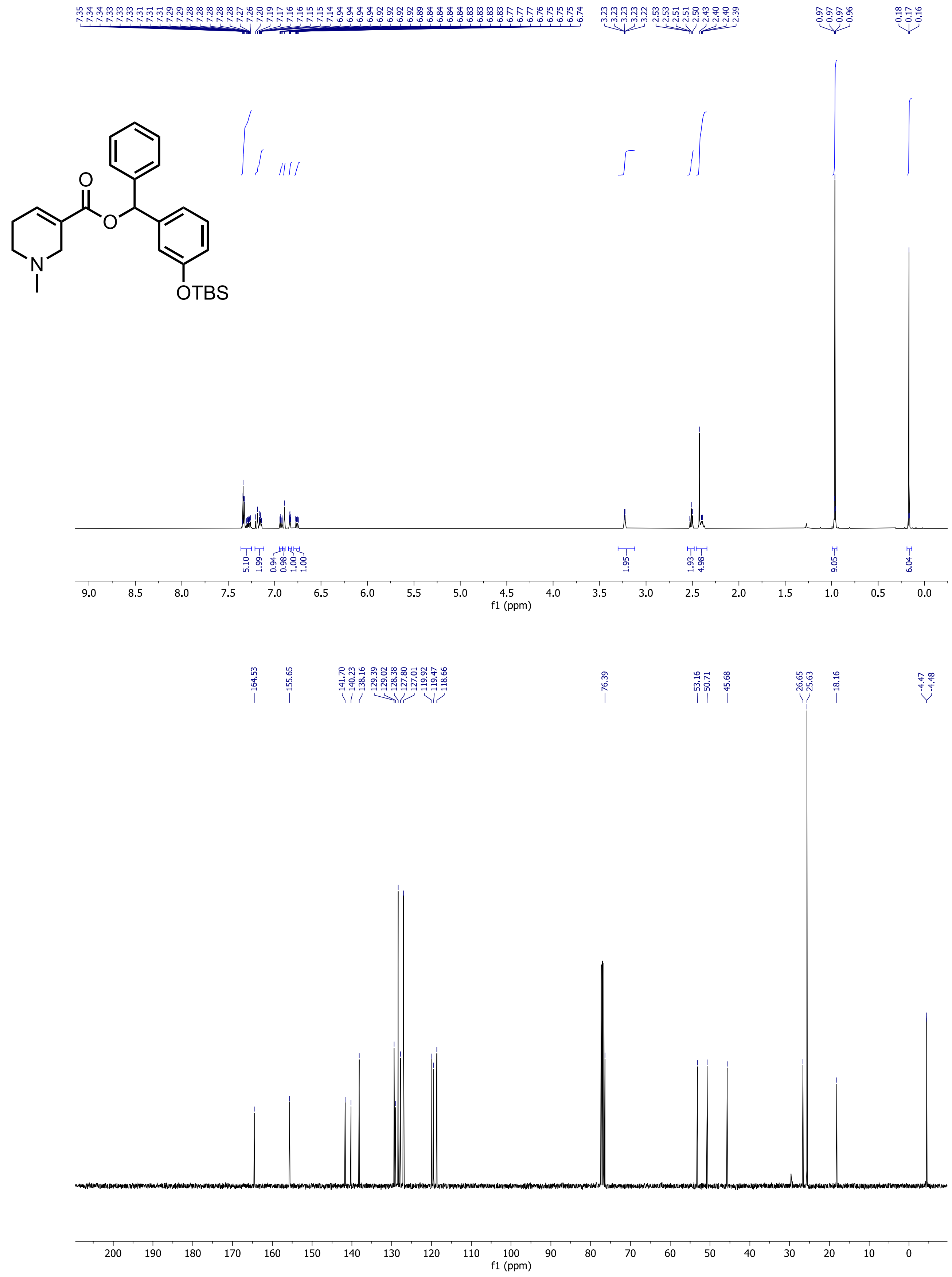

Figure S29. ^{1}H and ^{13}C NMR spectra of **15b**.

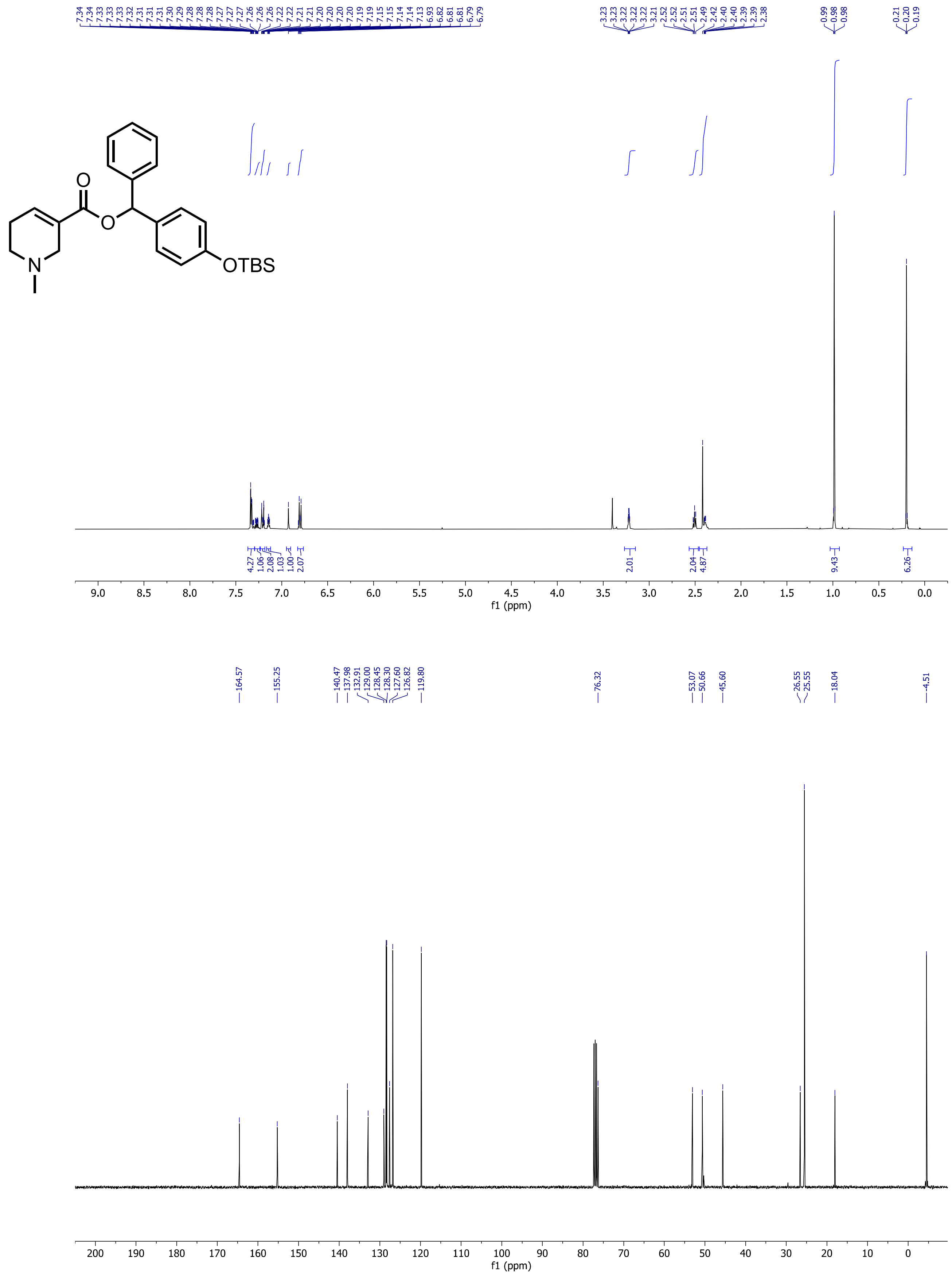

Figure S30. ^{1}H and ^{13}C NMR spectra of **15c**.

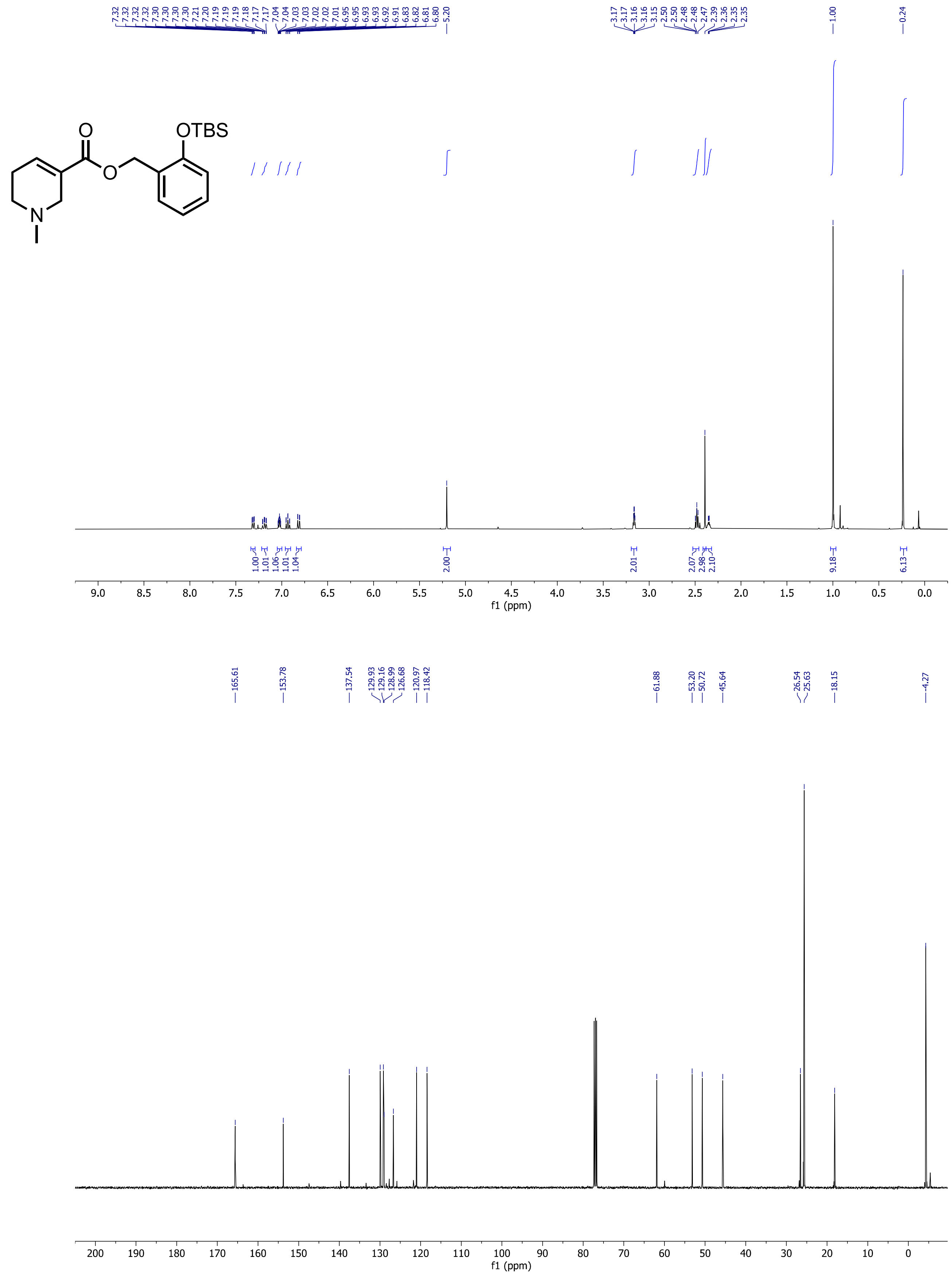

Figure S31. ^{1}H and ^{13}C NMR spectra of **16a**.

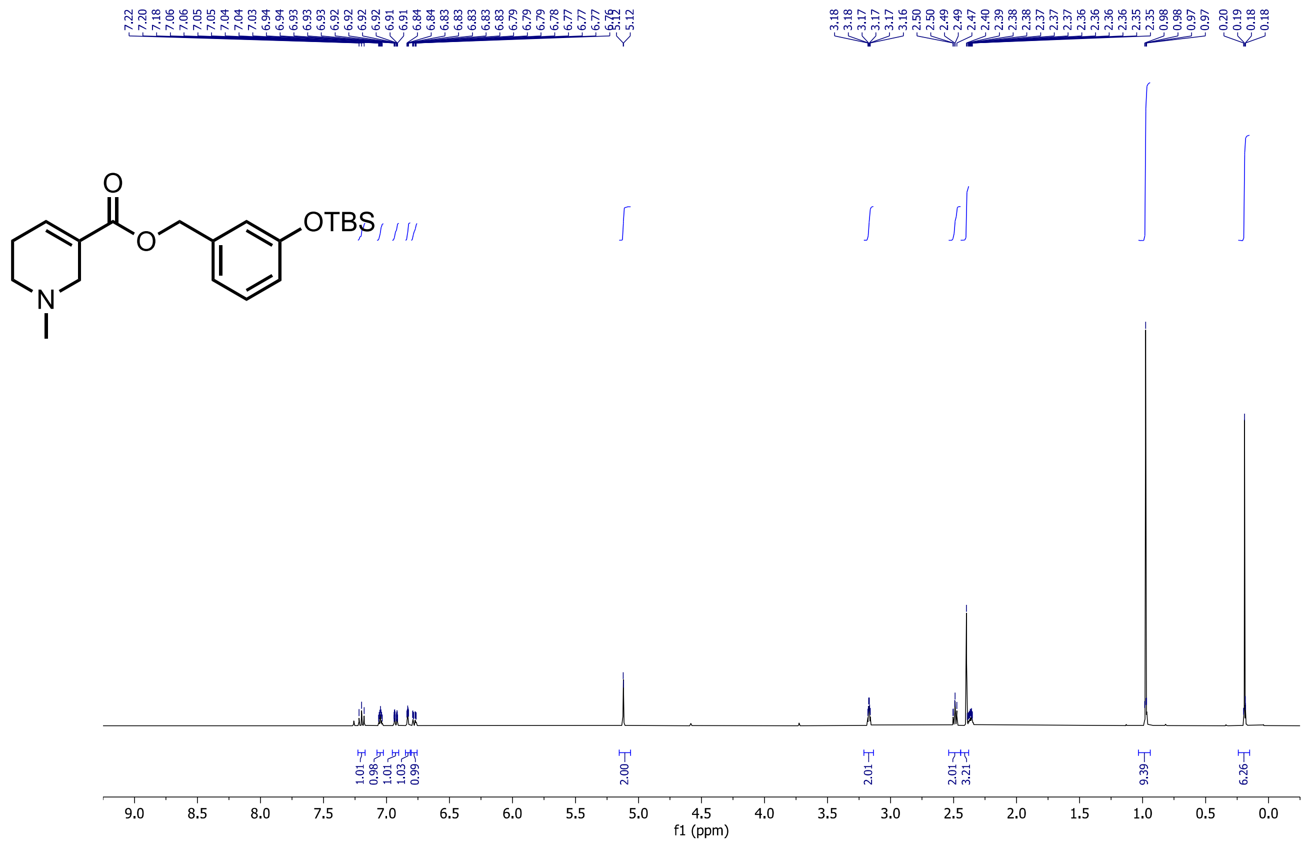

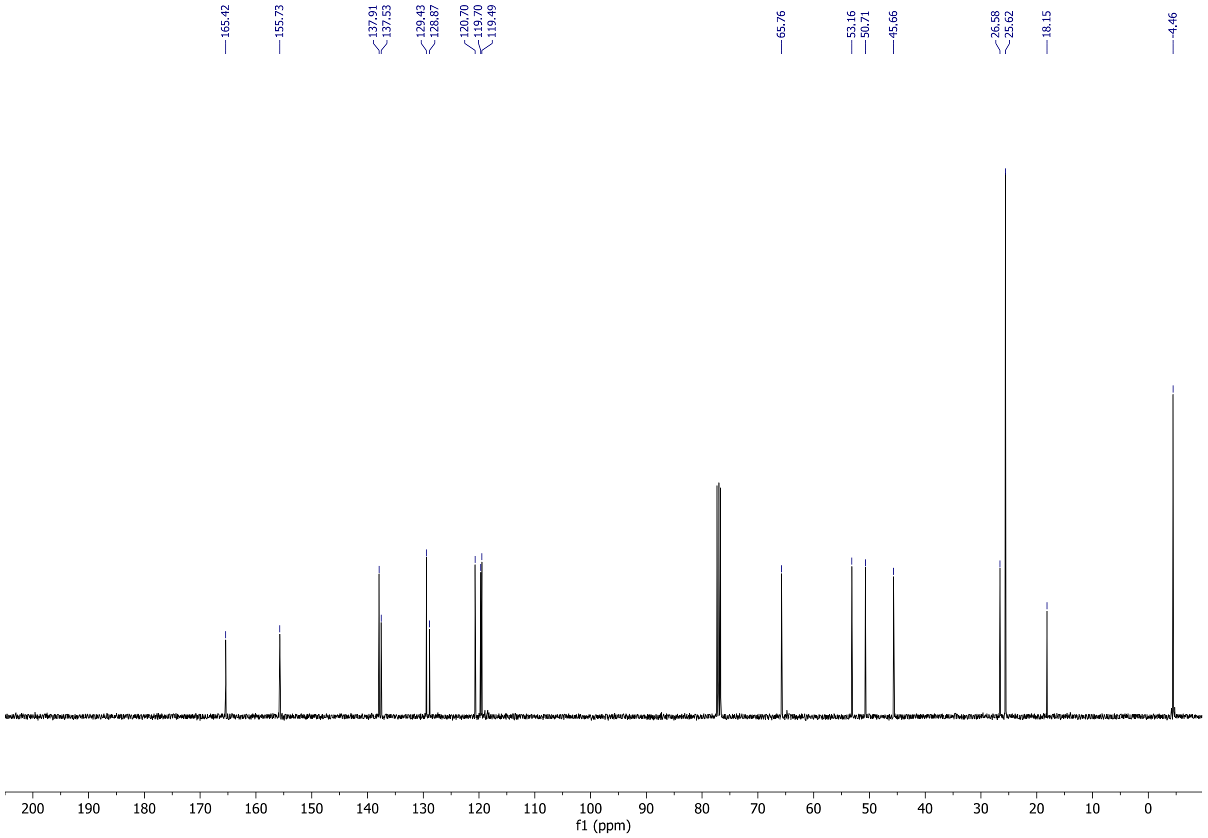

Figure S32. ^{1}H and ^{13}C NMR spectra of **16b**.

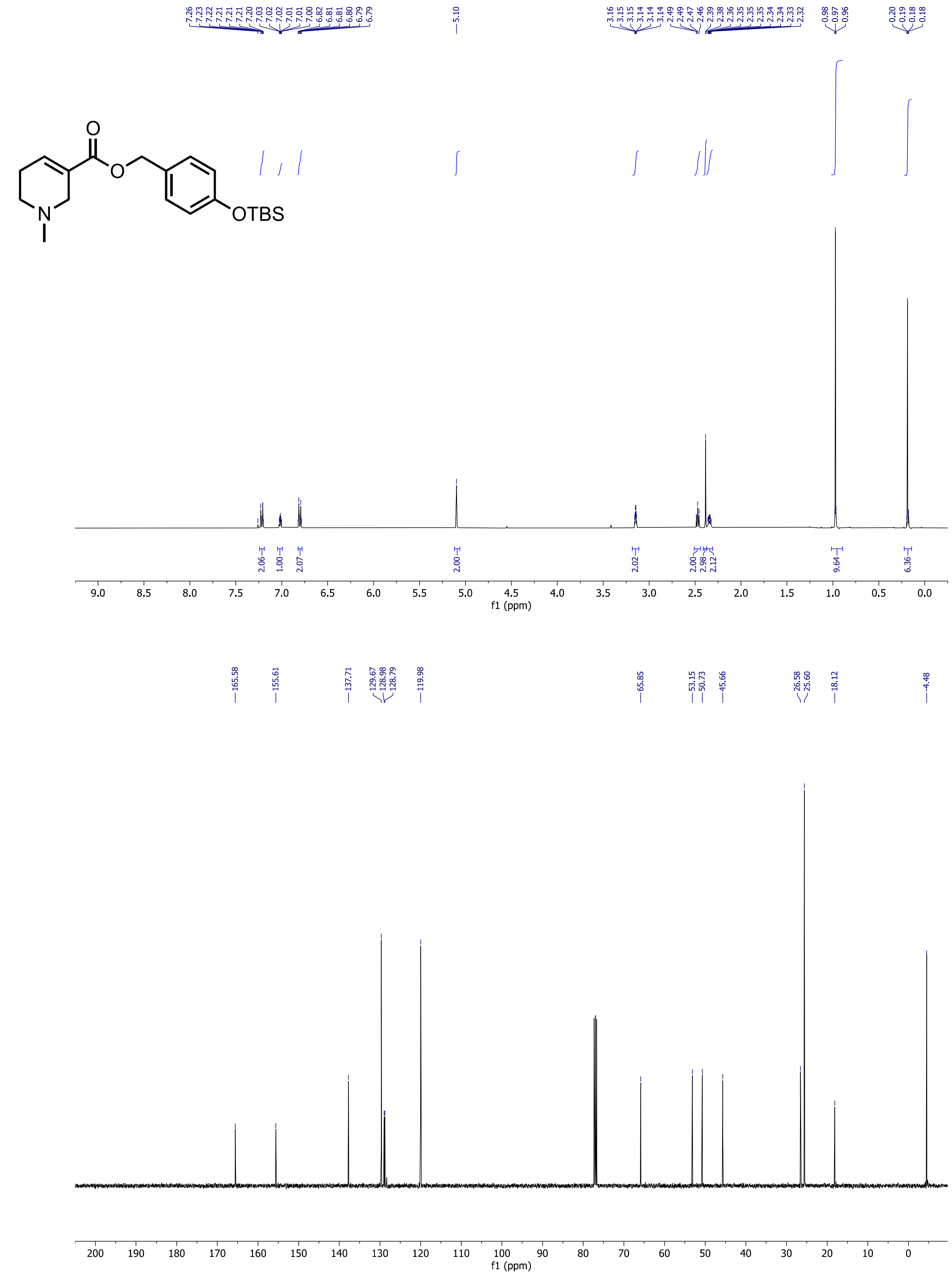

Figure 33. ^{1}H and ^{13}C NMR spectra of **16c**.

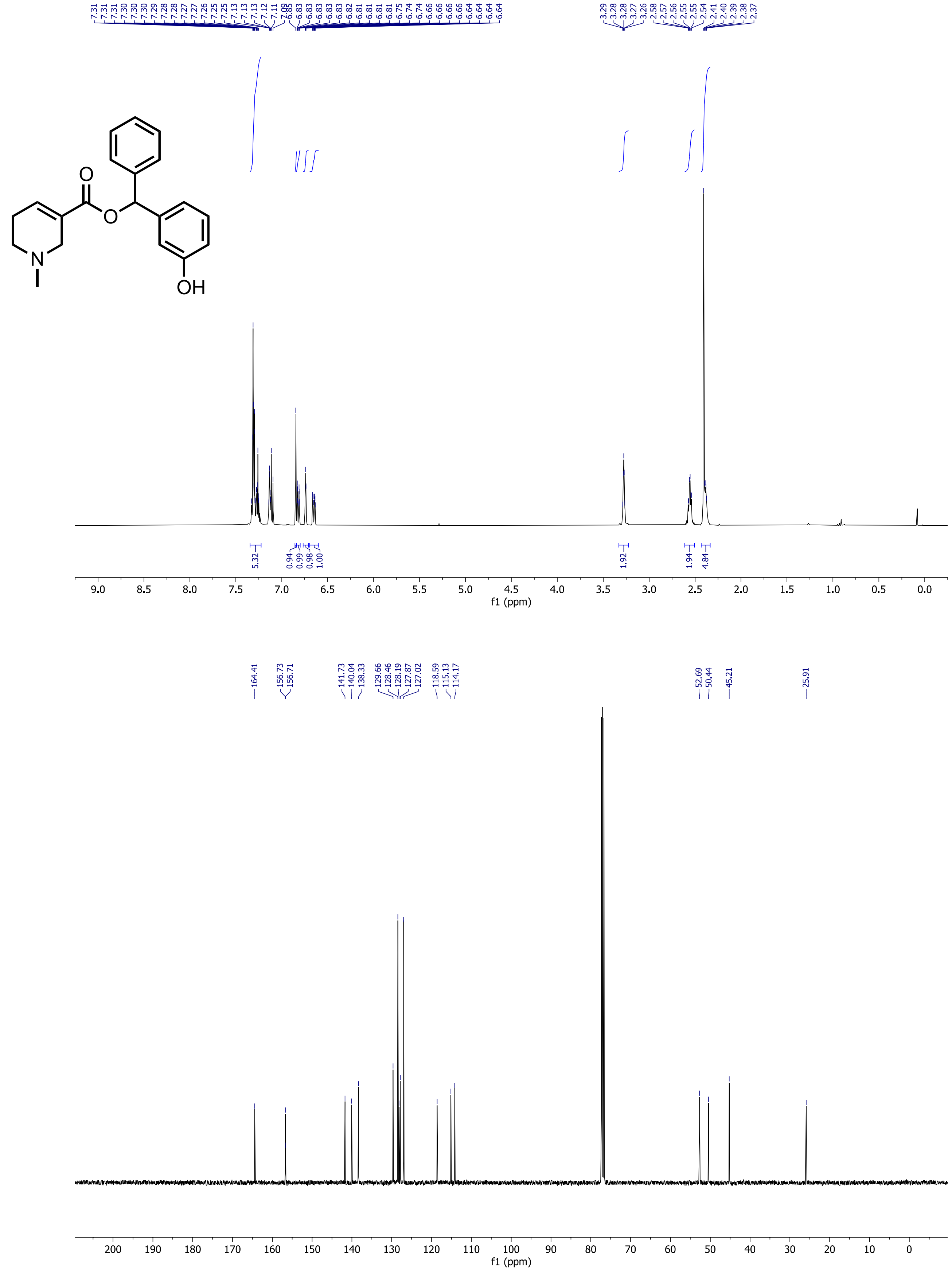

Figure S34. ^{1}H and ^{13}C NMR spectra of **17b**.

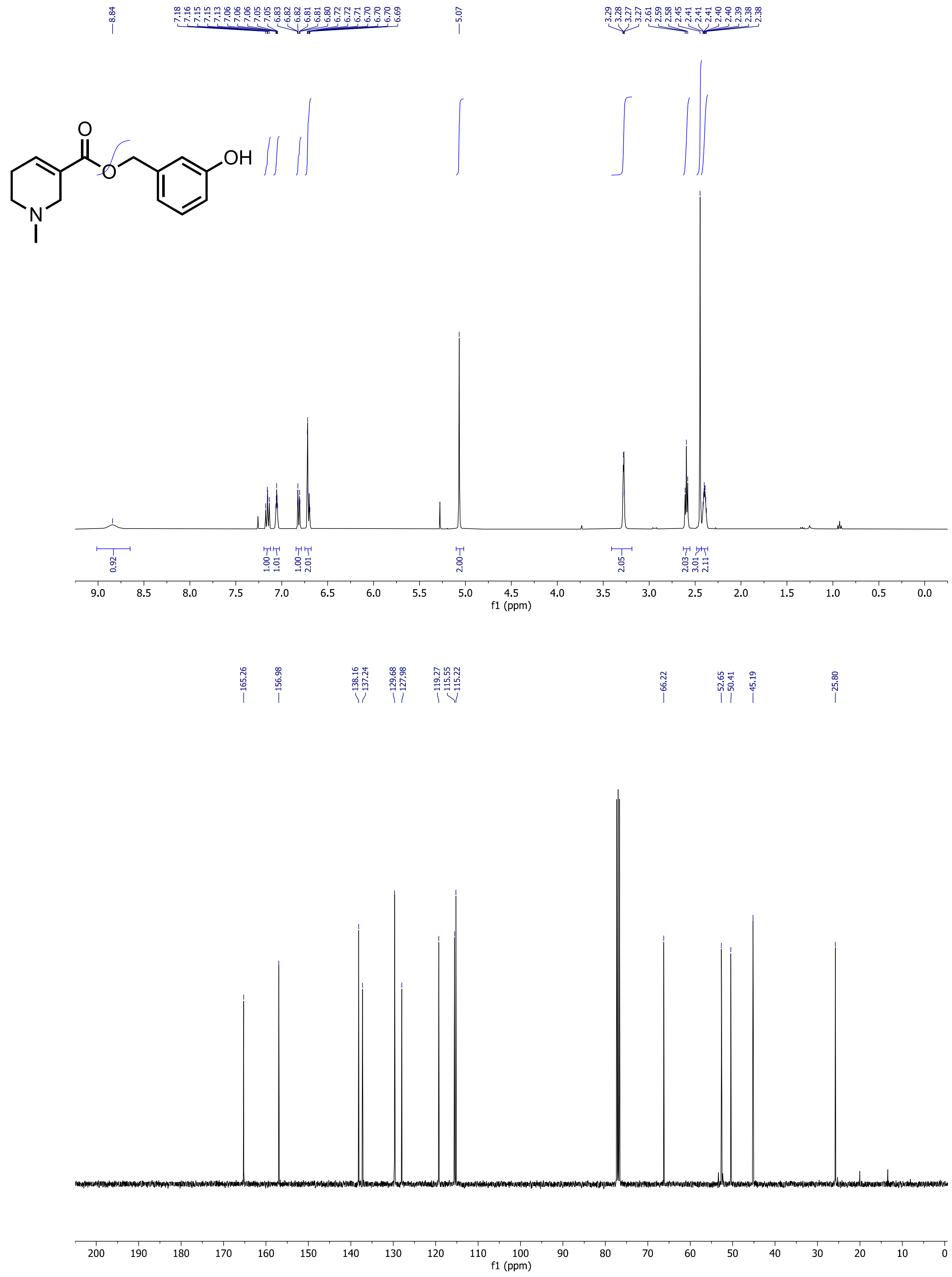

Figure S35. ^{1}H and ^{13}C NMR spectra of **18b**.

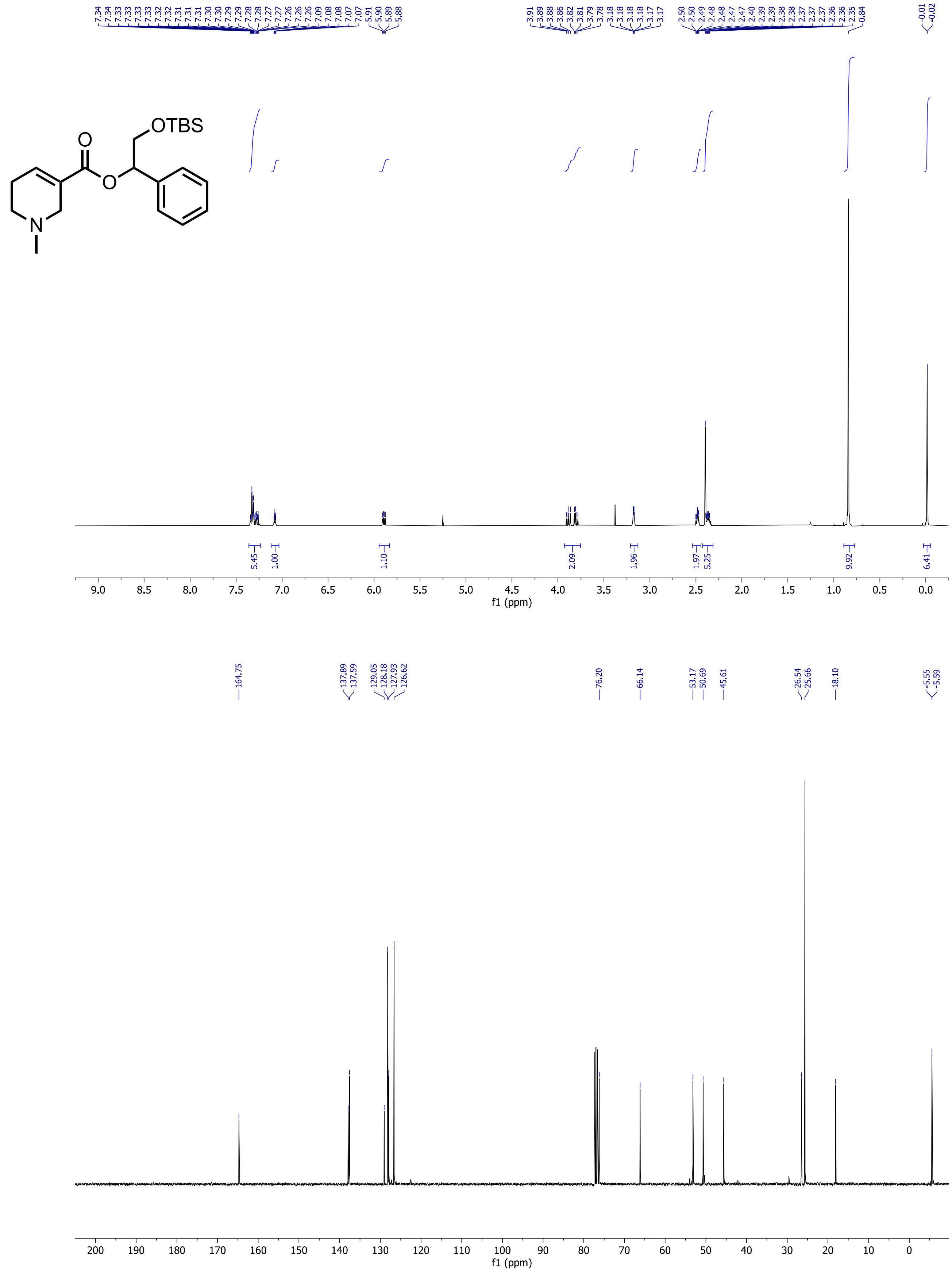

Figure S36. ^{1}H and ^{13}C NMR spectra of **21**.

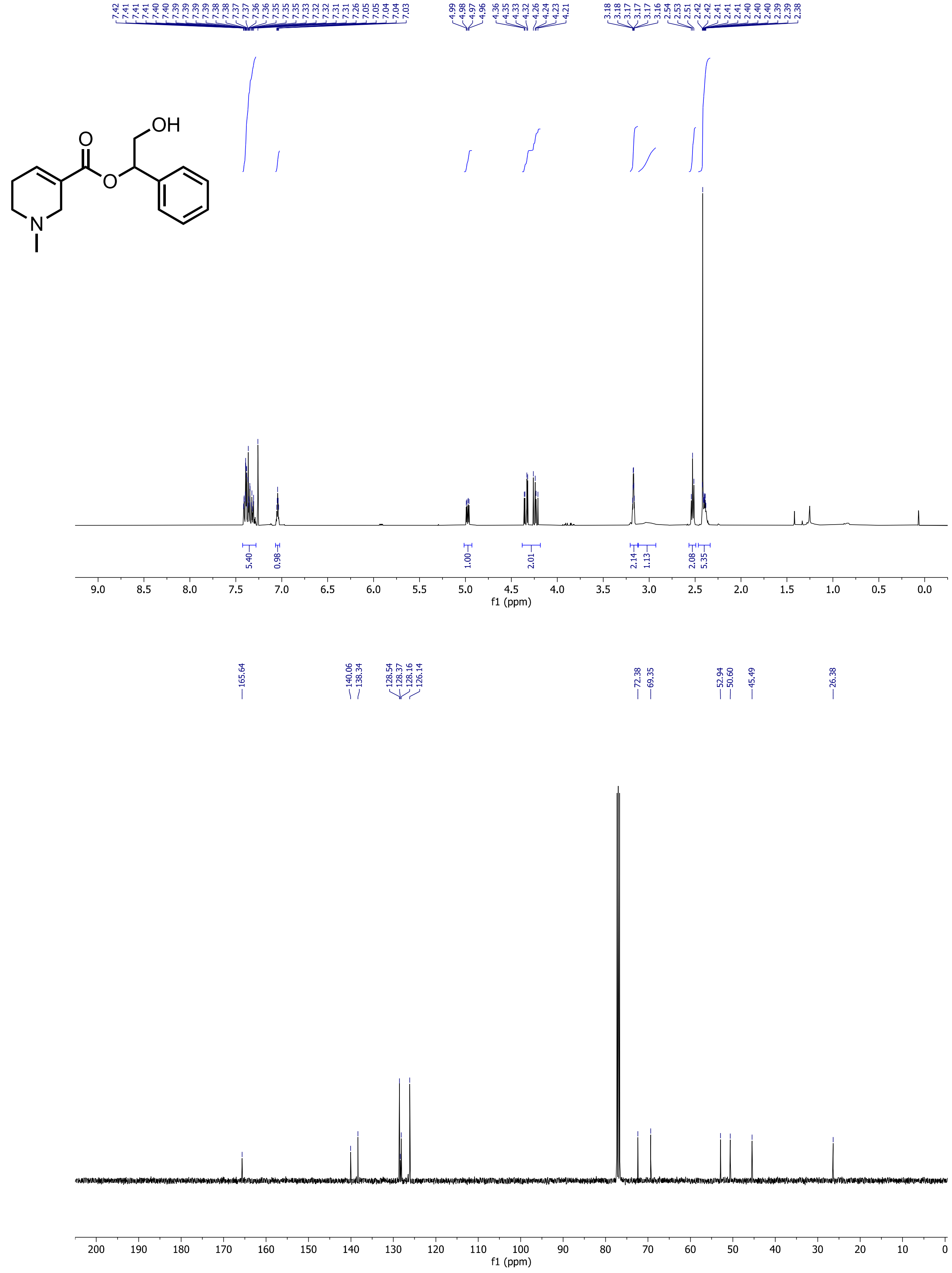

Figure S37. ^{1}H and ^{13}C NMR spectra of **22**

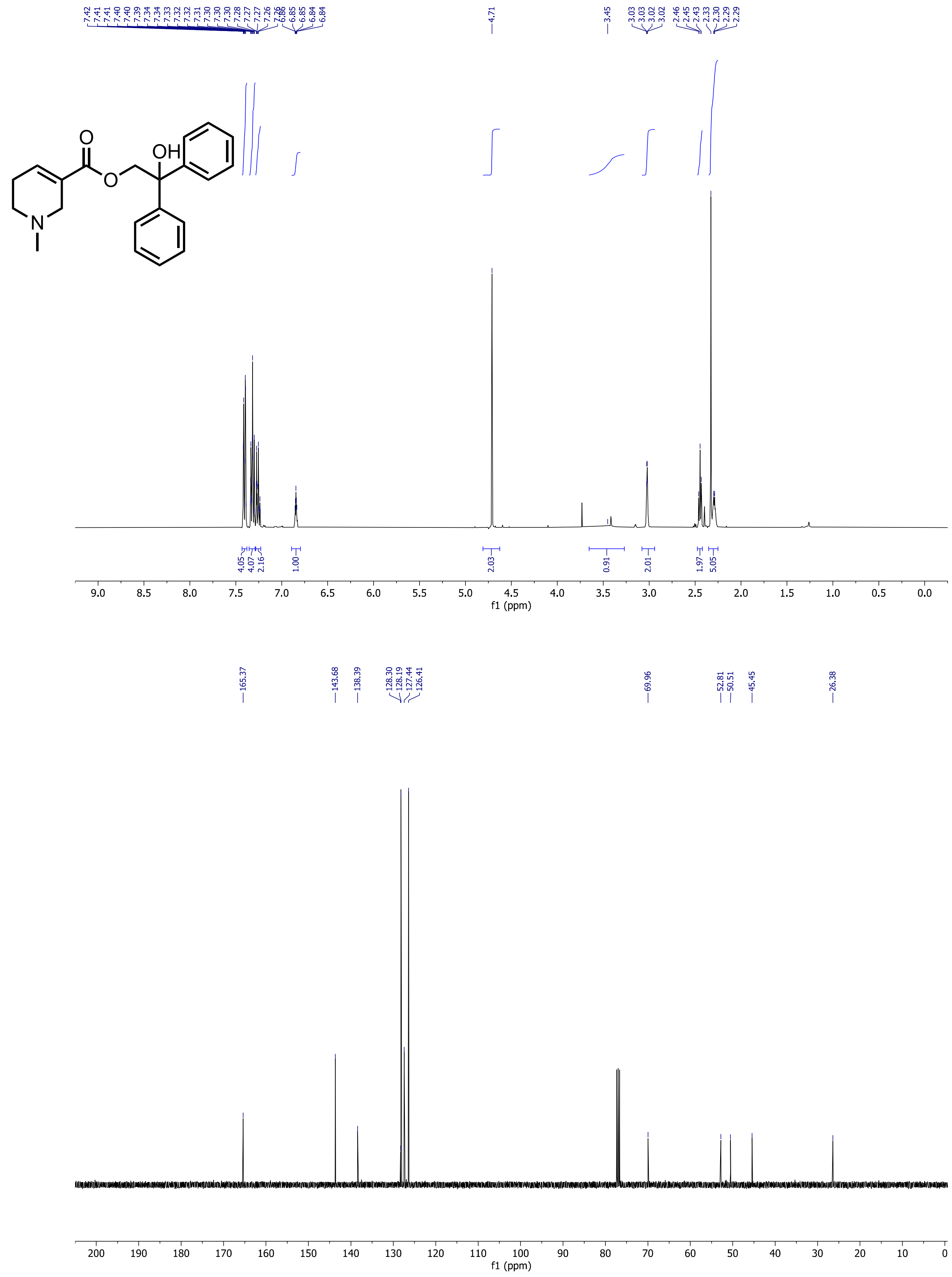

Figure S38. ^{1}H and ^{13}C NMR spectra of **25**.

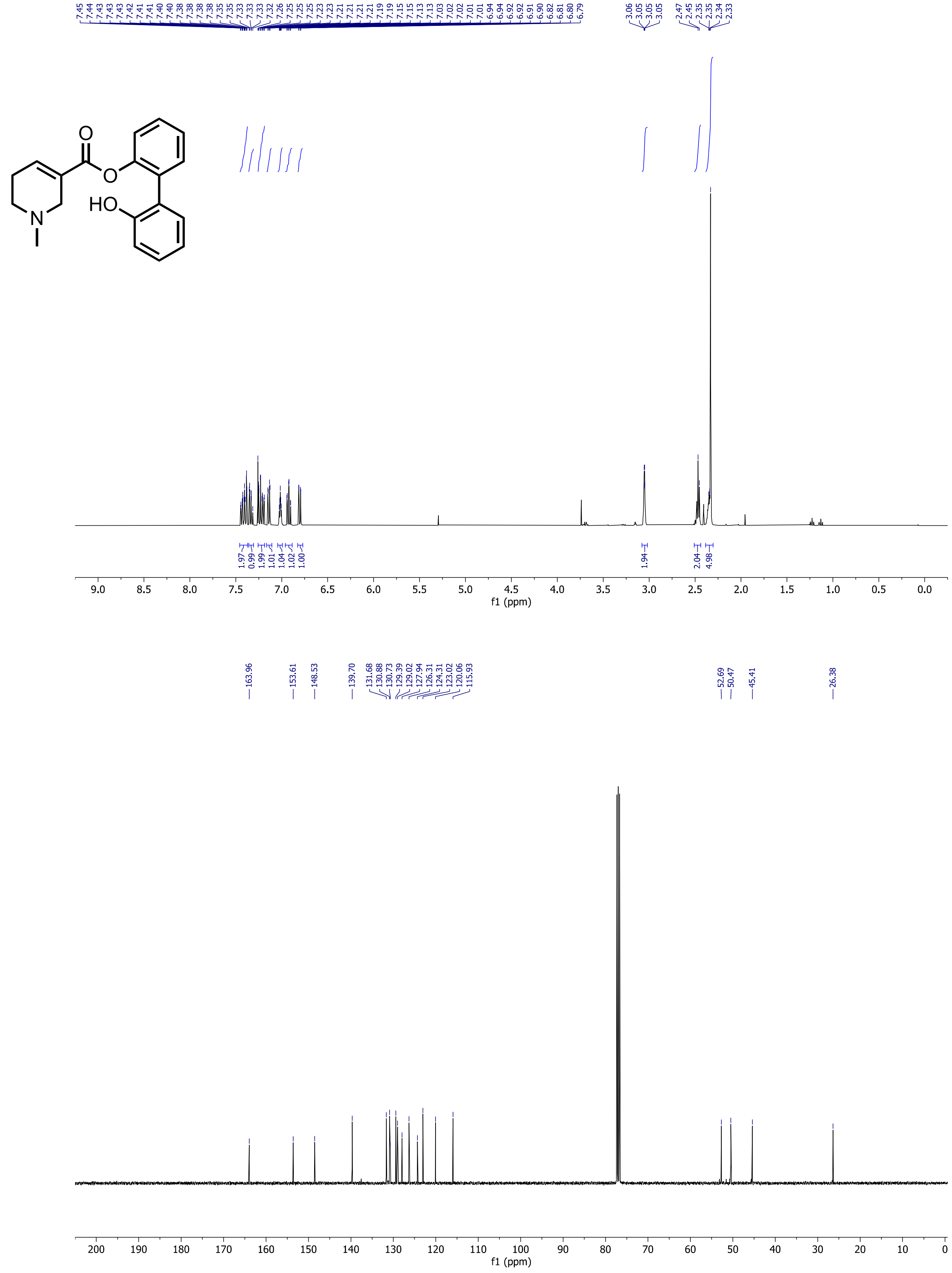

Figure S39. ^{1}H and ^{13}C NMR spectra of **27**.

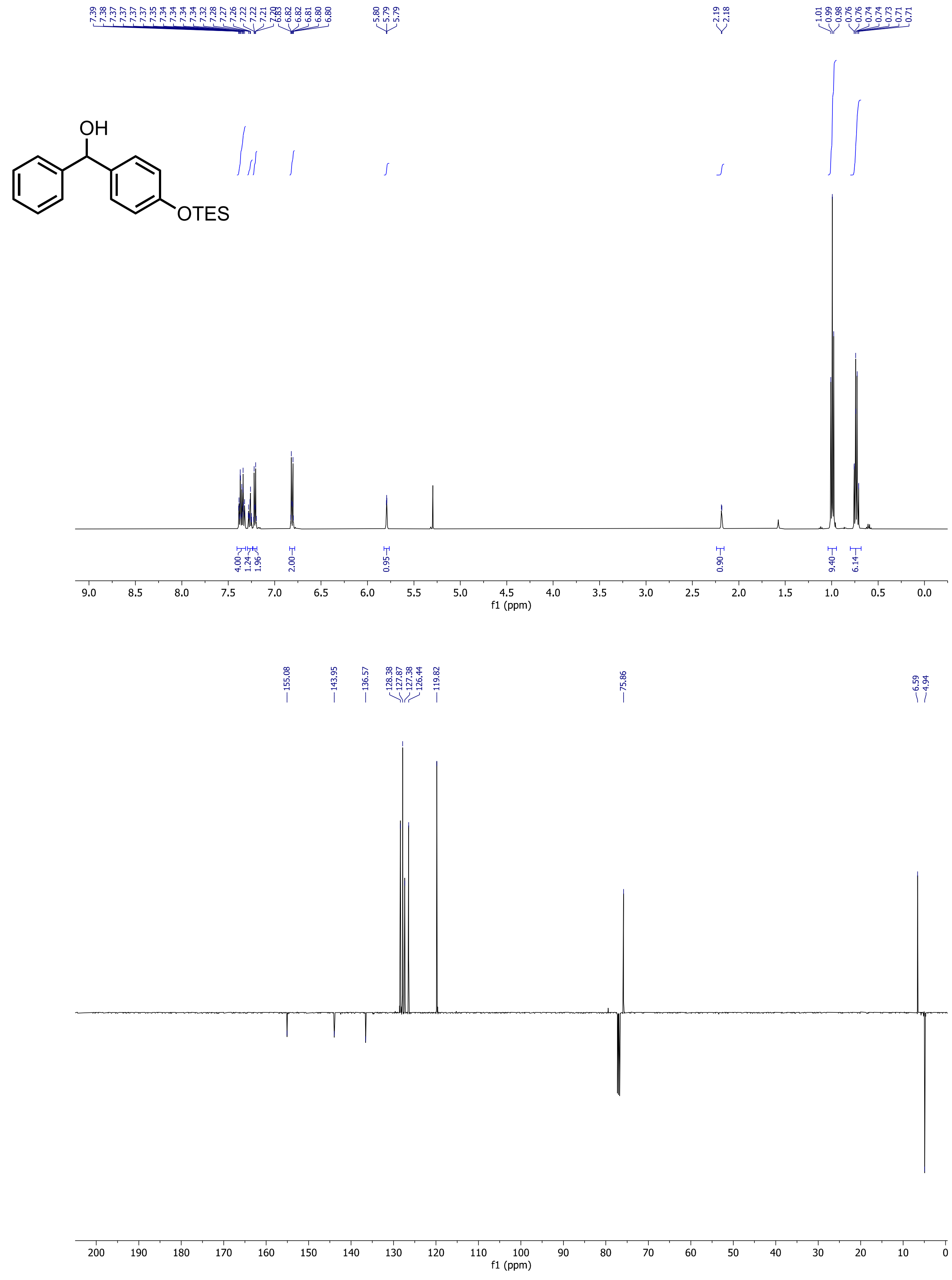

Figure S40. ^{1}H and ^{13}C NMR spectra of **32**.

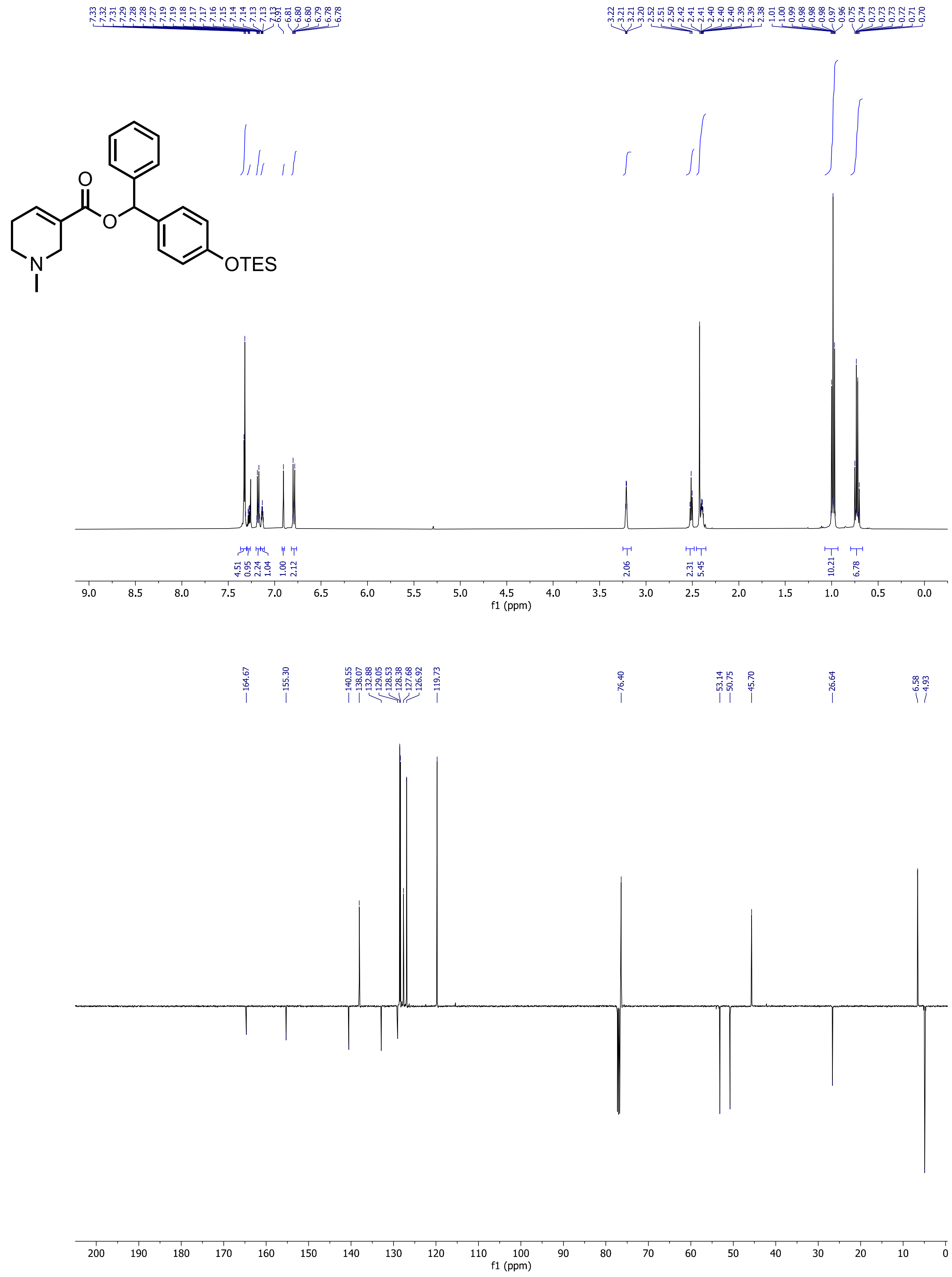

Figure S41. ^{1}H and ^{13}C NMR spectra of **35**.

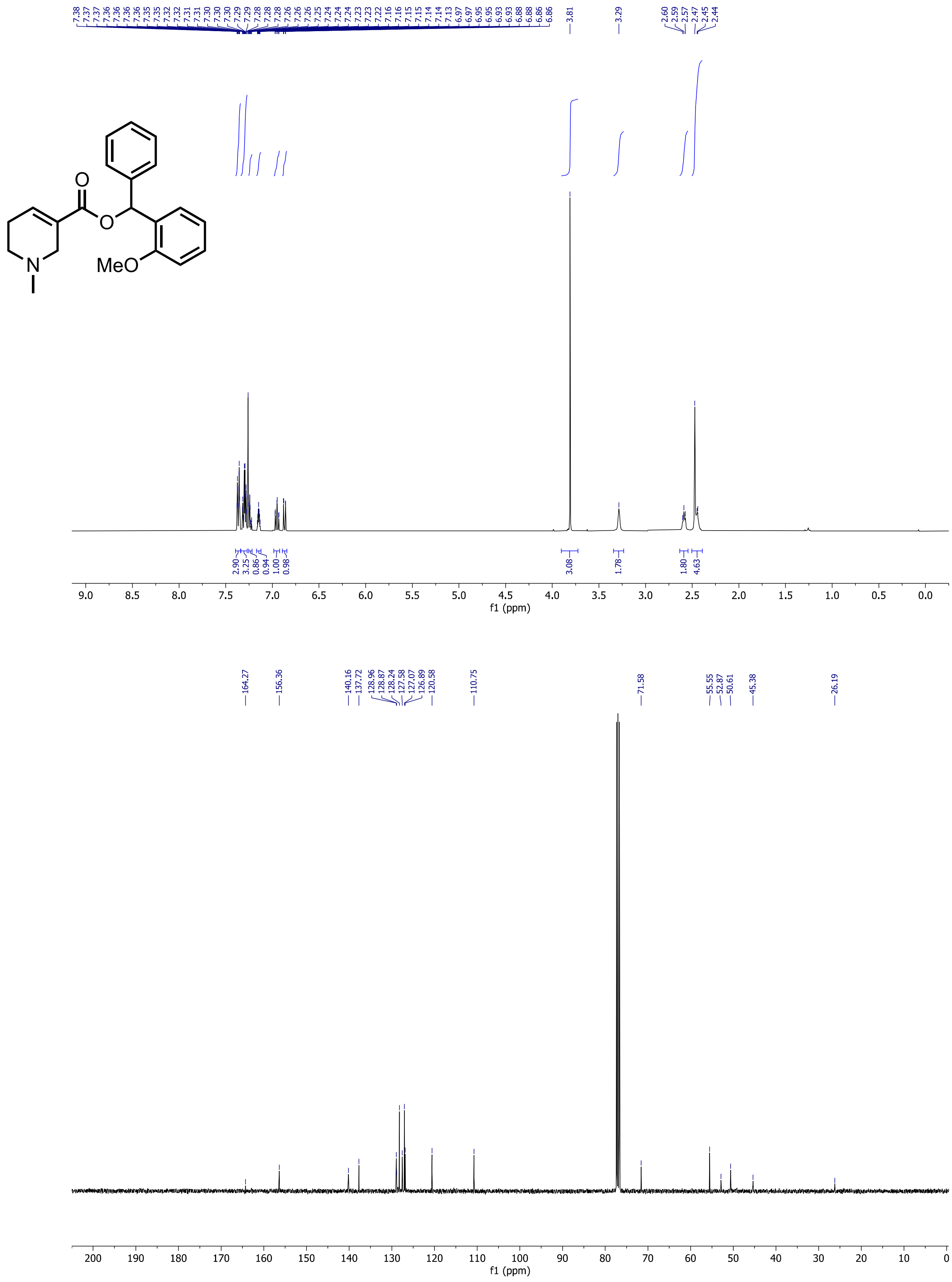

Figure S42. ^{1}H and ^{13}C NMR spectra of **36**.

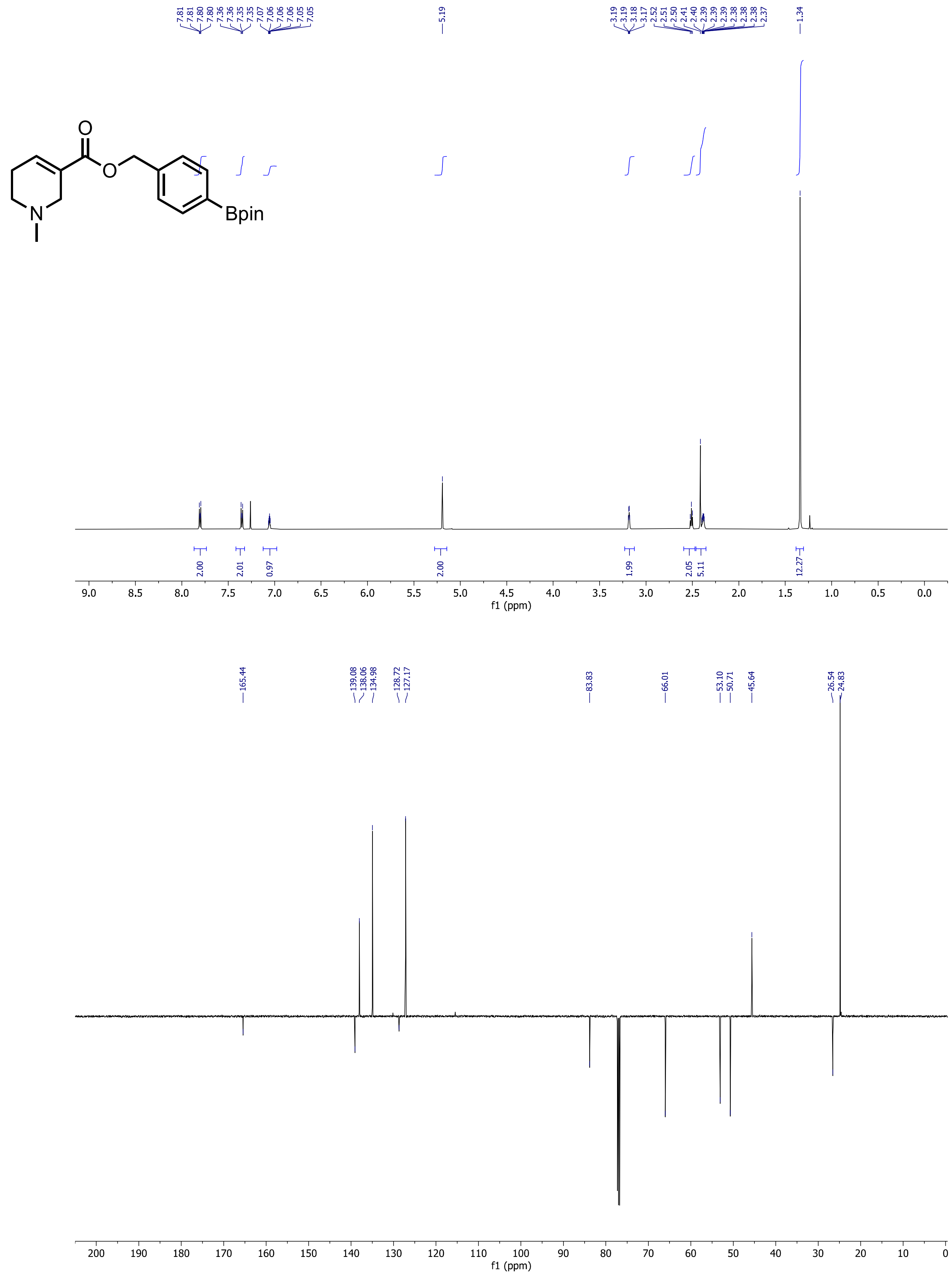

Figure S43. ^{1}H and ^{13}C NMR spectra of **37**.

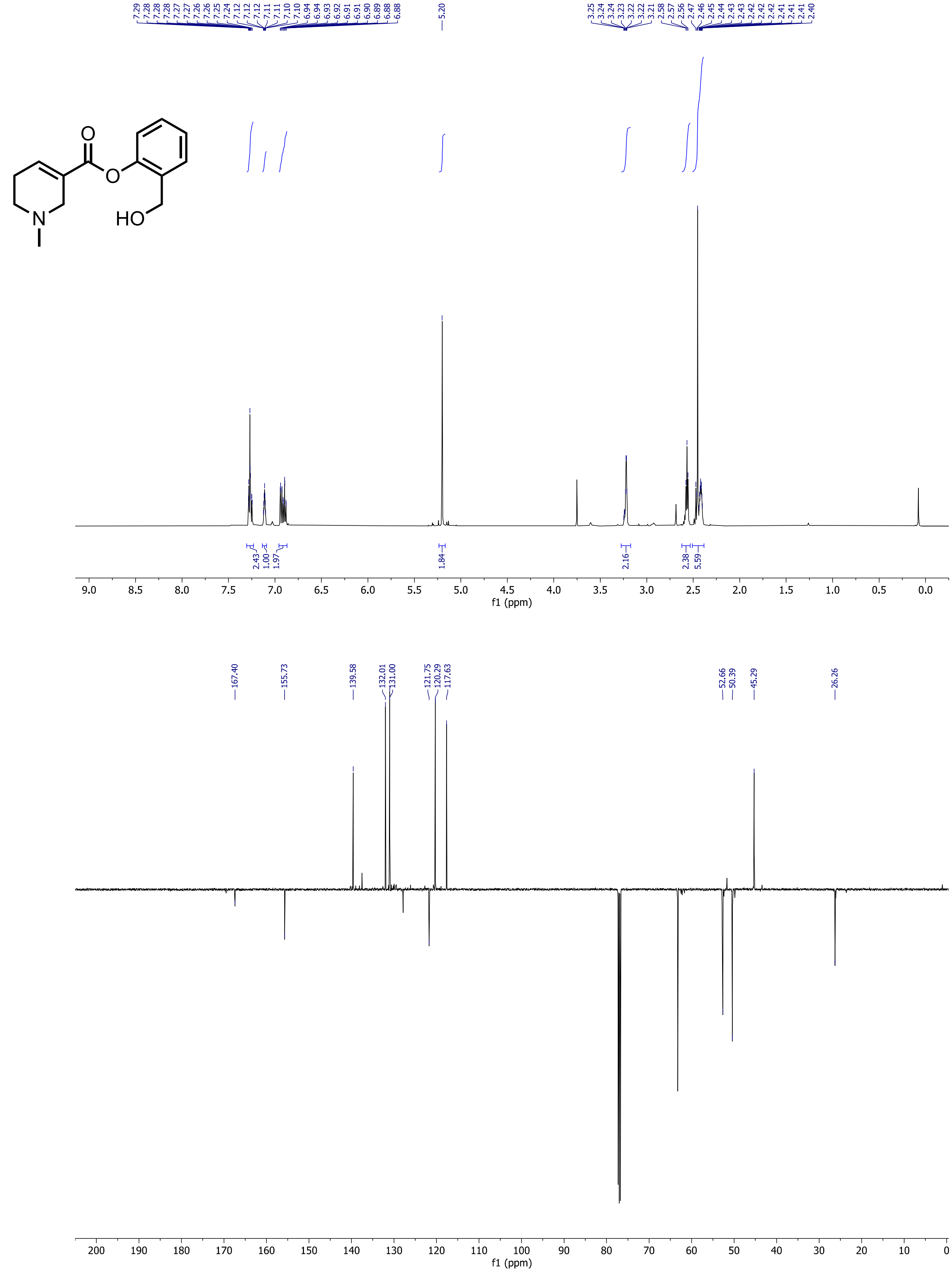

Figure S44. ^{1}H and ^{13}C NMR spectra of **44**.

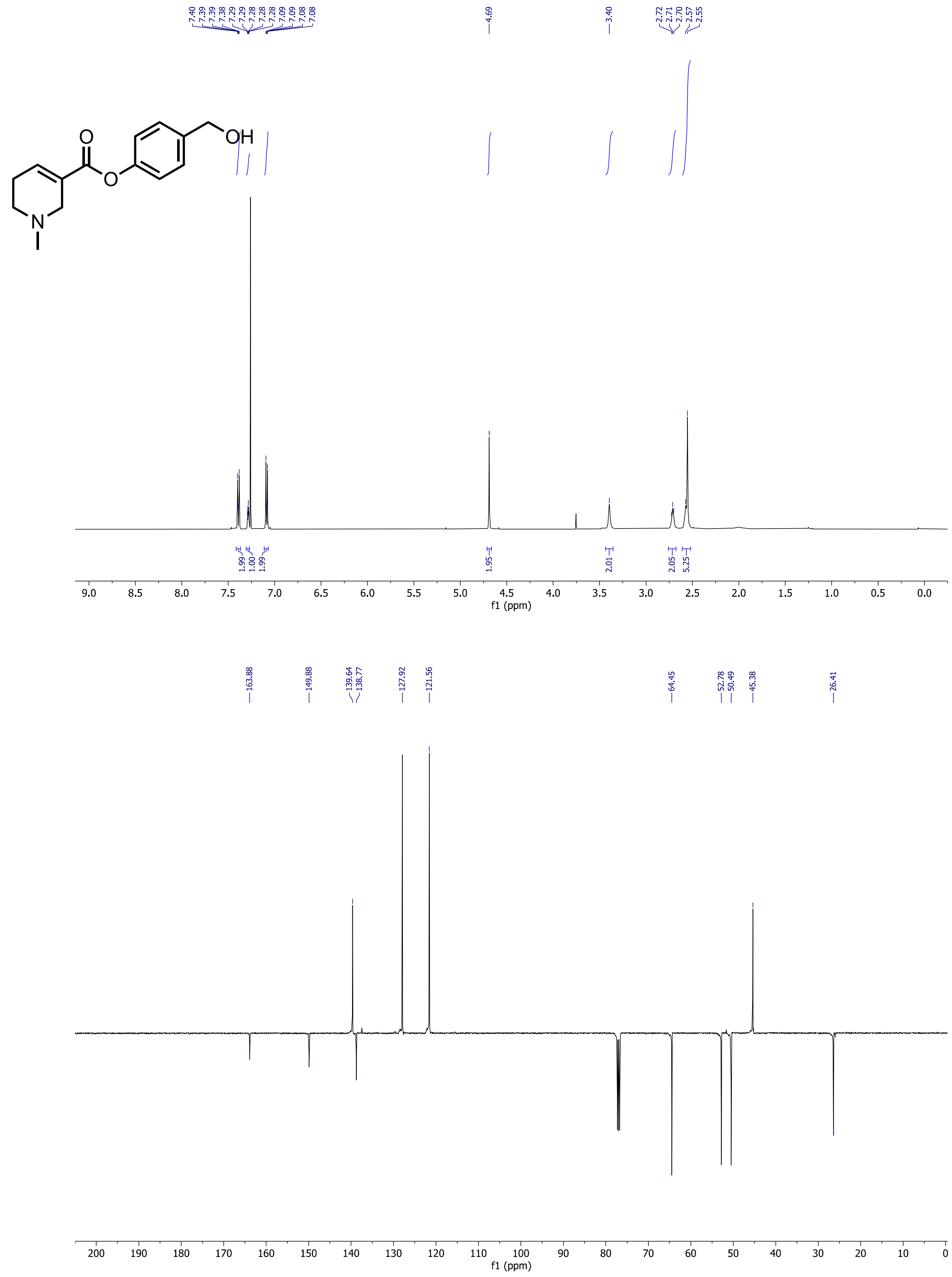

Figure S45. ^{1}H and ^{13}C NMR spectra of **45**.

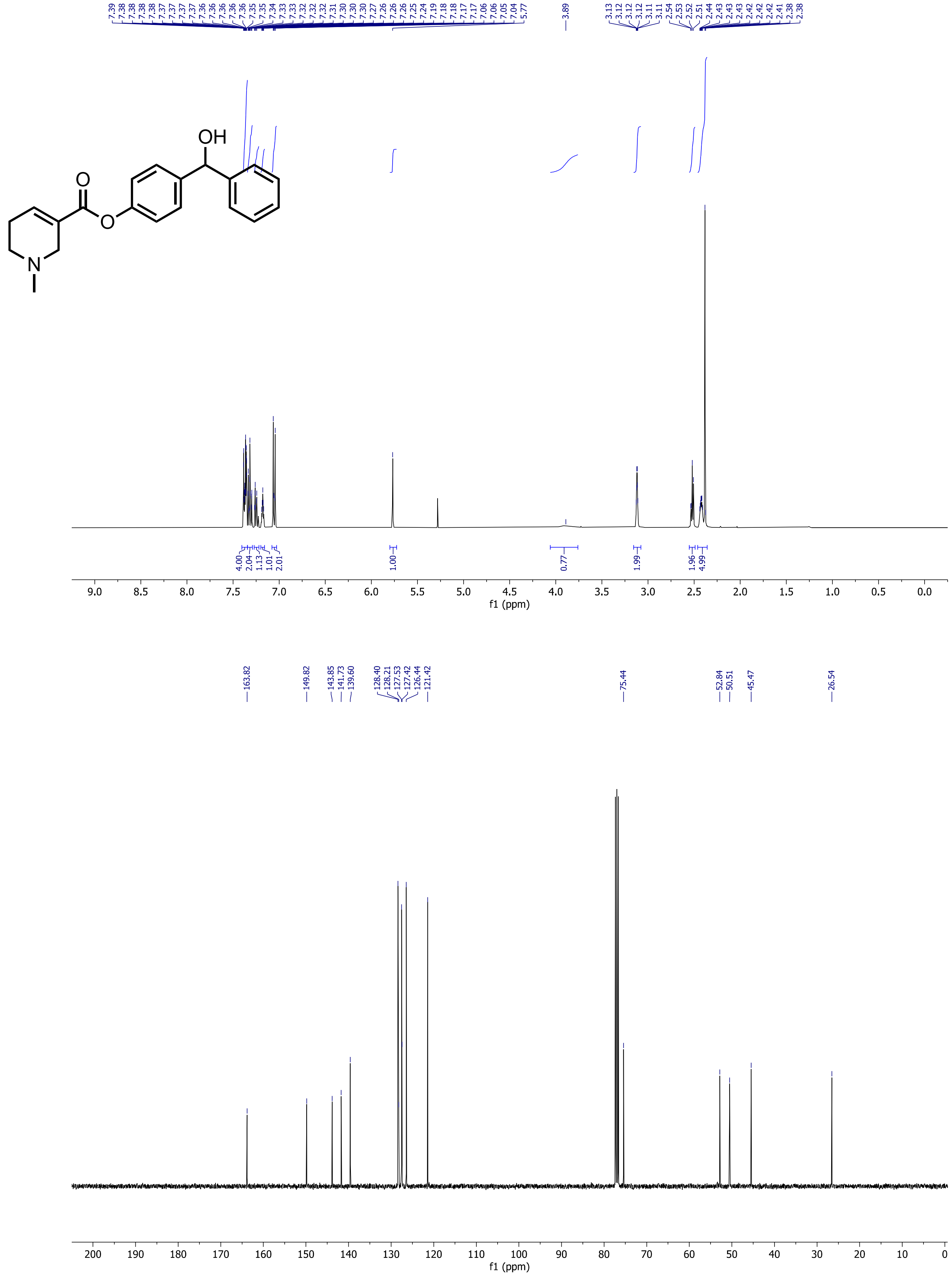

Figure S46. ^{1}H and ^{13}C NMR spectra of **46**.
